# Structural investigation of interactions between halogenated flavonoids and the lipid membrane along with their role as cytotoxic agents

**DOI:** 10.1038/s41598-024-61037-y

**Published:** 2024-05-08

**Authors:** Anita Dudek, Natalia Szulc, Aleksandra Pawlak, Paulina Strugała-Danak, Agnieszka Krawczyk-Łebek, Martyna Perz, Edyta Kostrzewa-Susłow, Hanna Pruchnik

**Affiliations:** 1 Department of Physics and Biophysics, Faculty of Biotechnology and Food Sciences, Wrocław University of Environmental and Life Sciences, Norwida 25, 50-375 Wrocław, Poland; 2 Department of Pharmacology and Toxicology, Faculty of Veterinary Medicine, Wrocław University of Environmental and Life Sciences, Norwida 31, 50-375 Wrocław, Poland; 3 Department of Food Chemistry and Biocatalysis, Faculty of Biotechnology and Food Sciences, Wrocław University of Environmental and Life Sciences, Norwida 25, 50-375 Wrocław, Poland

**Keywords:** Halogenation, Flavonoids derivatives, Liposomes, ATR-FTIR, Cell viability, Fluorescent probes, Biochemistry, Biophysics, Molecular biology

## Abstract

This study focuses on understanding the structural and molecular changes in lipid membranes under the influence of six halogenated flavonoid derivatives differing in the number and position of substitution of chlorine and bromine atoms (D1–D6). Utilizing various analytical techniques, including fluorometric methods, dynamic light scattering (DLS), attenuated Fourier transform infrared spectroscopy (ATR- FTIR), and FT-Raman spectroscopy, the research aims to elucidate the mechanisms underlying the interaction of flavonoids with cell membranes. Additionally, the study includes in silico analyses to explore the physicochemical properties of these compounds and their potential pharmaceutical applications, along with toxicity studies to assess their effects on cancer, normal, and red blood cells. Our study showed the ability of halogenated derivatives to interact mostly with the outer part of the membrane, especially in the lipid heads region however, some of them were able to penetrate deeper into the membrane and affect the fluidity of hydrocarbon chains. The potential to reduce cancer cell viability, the lack of toxicity towards erythrocytes, and the favourable physicochemical and pharmacokinetic properties suggest these halogenated flavonoids potential candidates for exploring their potential for medical use.

## Introduction

**Figure 1:**
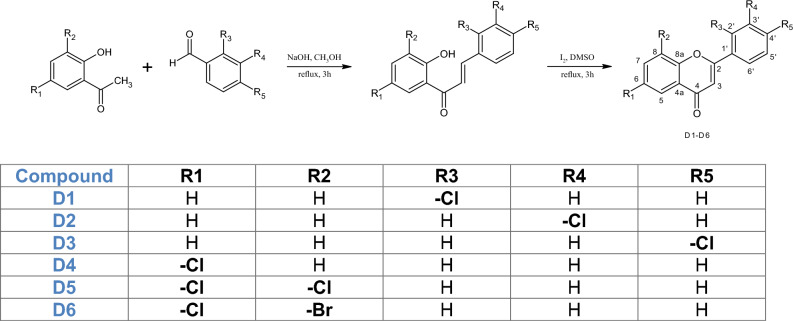
Synthesis of 2′-chloroflavone (D1), 3′-chloroflavone (D2), 4′-chloroflavone (D3), 6- chloroflavone (D4), 6,8-dichloroflavone (D5), and 8-bromo-6-chloroflavone (D6).

Flavonoids have garnered considerable attention due to their wide-ranging therapeutic benefits. This is attributed to their prevalence in the natural world and their diverse pharmacological properties including anticancer^1,2^, anti-inflammatory^3^, neuroprotective^4^, cardioprotective, and antioxidant properties^5^. Due to their poor solubility, low bioactivity, and poor pharmacokinetic parameters, the use of flavonoids as drugs is significantly limited^6^. The incorporation of halogens into the composition of natural substances or artificially created compounds frequently enhances both their biological effectiveness and physical and chemical characteristics^7^. Halogenated flavonoid derivatives have demonstrated significant anticancer, anti-proliferative, and growth-inhibitory effects against various cancer cell lines^8,9^. Additionally, they exhibit antiangiogenic properties and can inhibit tube formation in endothelial cells^10^. Halogenation of natural flavonoids can contribute to the design of new ligands for tumour-associated human kinase CK2 and the observed effect is even more potent than for the reference CK2 inhibitors^11^. Other research groups have also revealed the important role of halogenated flavonoid-based compounds for their capacity to inhibit the activity of α-amylase inhibitors and highlighted its role as a potential candidate for the therapeutic approach to obesity and diabetes^12^. Flavonoid interactions with cell membranes represent a significant target and potentially can shed light on their mechanisms of action. The primary suggested biochemical mechanisms underlying flavonoid activity involve scavenging free radicals, modifying membrane structure and stability, and influencing membrane permeability^13^. Various elements impact how flavonoids interact with membranes, including their hydrophobicity, planarity and polarity. The structural modifications of the flavonoids such as halogenation, hydroxylation, or glycosylation are linked to an affinity for lipid membranes, impacting on electrostatic interactions with polar regions as well as alterations in the degree of fluidity of the membrane^14^. Due to the limited literature on halogenated flavonoids and their interaction with macromolecules, we decided to evaluate the interaction of six promising bromo- and chloroflavone derivatives^15^ (Fig. 1) with cell membrane models. Two liposome systems: MODEL consisting of DPPC and 20% cholesterol and MIMIC that mimicked the lipid composition of tumour cells were used as models of artificial lipid membranes. Utilizing fluorometric techniques, dynamic light scattering (DLS), attenuated Fourier transform infrared spectroscopy (ATR-FTIR) and FT-Raman spectroscopy we were able to assess the structural and molecular changes in lipid membranes under the influence of the tested flavonoid derivatives. Our research has been enriched by in silico analyses indicating basic information on the physicochemical properties of the compounds and their potential in the pharmaceutical industry. Additionally, we conducted toxicity studies consisting of viability analysis of canine tumour cells and normal cells as well as haemolytic studies focusing on the degradation of red blood cells under the influence of the tested compounds.

## Results

### Physicochemical properties prediction based on SwissADME tool

All derivatives (D1–D6) have similar molecular weights, ranging from 256.68 to 335.58 g/mol, indicating a relatively consistent structural size across the compounds (Table 1). The number of rotatable bonds (RB) is 1 for all compounds, suggesting a degree of structural rigidity, which can impact drug-receptor interactions. Each compound has 2 hydrogen bond acceptors (HBA) and no hydrogen bond donors (HBD), reflecting certain aspects of their potential interactions with biological targets. Moreover, the topological polar surface area (TPSA) is consistently 30.21 Å^2^ for all compounds, providing insight into their potential to cross biological membranes. Lipophilicity was assessed based on the logarithm of the n-octanol/water partition coefficient, which was calculated using the Consensus LogP_o/w_. The logP values range from 3.70 to 4.22, all above the ideal range for good oral bioavailability^16^.

**Table 1:** The calculated physicochemical parameters of all halogenated derivatives.

Compound	MW (g/mol)	RB	HBA	HBD	TPSA (Å^2^)	LogP_o/w_	WS
D1	256.68	1	2	0	30.21	3.70	Moderately soluble
D2	256.68	1	2	0	30.21	3.71	Moderately soluble
D3	256.68	1	2	0	30.21	3.71	Moderately soluble
D4	256.68	1	2	0	30.21	3.78	Moderately soluble
D5	291.13	1	2	0	30.21	4.13	Moderately soluble
D6	335.58	1	2	0	30.21	4.22	Moderately soluble

Where: *MW* molecular weight, *RB* num. rotatable bonds, *HBA* num. H-bond acceptors, *HBD* num. H-bond donors, *TPSA* topological polar surface area, *LogP*_*o/w*_ lipophilicity calculated as the average value from five models, WS water solubility calculated based on Delaney et al.^17^.

### Effects on the hydrophilic and hydrophobic region of the lipid membrane—fluorometric studies

**Figure 2:**
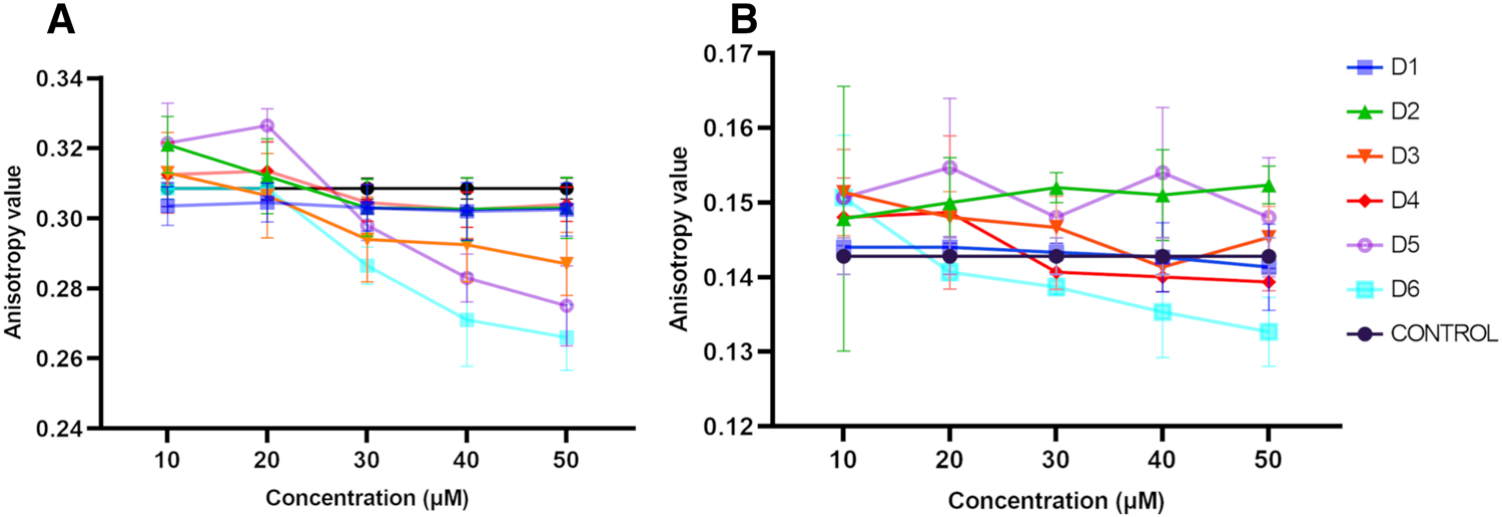
Anisotropy value for MODEL membrane (**A**) and MIMIC membrane (**B**). Error bars represent mean ± standard deviation (SD) (n = 4).

Changes in the ordering of the inner region of the lipid membranes were observed, as indicated by changes in membrane anisotropy values based on the behaviour of DPH probe (Fig. 2). In the case of MIMIC membrane, the presence of compound D6 resulted in a reduction in anisotropy values, particularly at a concentration of 50 μM. Similar effects were observed for D4, but only at the highest concentration. The observed decrease of anisotropy values suggests a potential increase in membrane fluidity. This is because the motion of the probe in the membrane is less restricted, allowing for a more fluid membrane environment. The anisotropy value of the DPH probe associated with the MODEL membrane was lower than that of the control, suggesting an increase in the fluidity of the membrane environment. This effect was particularly evident for compounds D6, D5, and D3. The measurements were performed below the temperature of the main phase transition for DPPC (42 °C). However, the temperature of 37 °C may have caused slight changes in the fluidity of the model membrane. The distribution of the lipid heads was uneven, as was the distribution of cholesterol, which may have been the cause of the integration of the compound into the membrane. Thus, access to the deeper layers of the membrane was facilitated, especially for most lipophilic compounds. The D6 compound, being the most lipophilic, is most likely to incorporate into the polar region of the lipid bilayer. This is true for both types of liposome membranes. However, this lipophilicity-affinity correlation is not observable for the other compounds. It can be suggested that the interaction between membranes and flavonoids is not solely based on lipophilicity, but also on the molecular structure of the flavonoids and the halogen substitutes attached to them.

**Figure 3:**
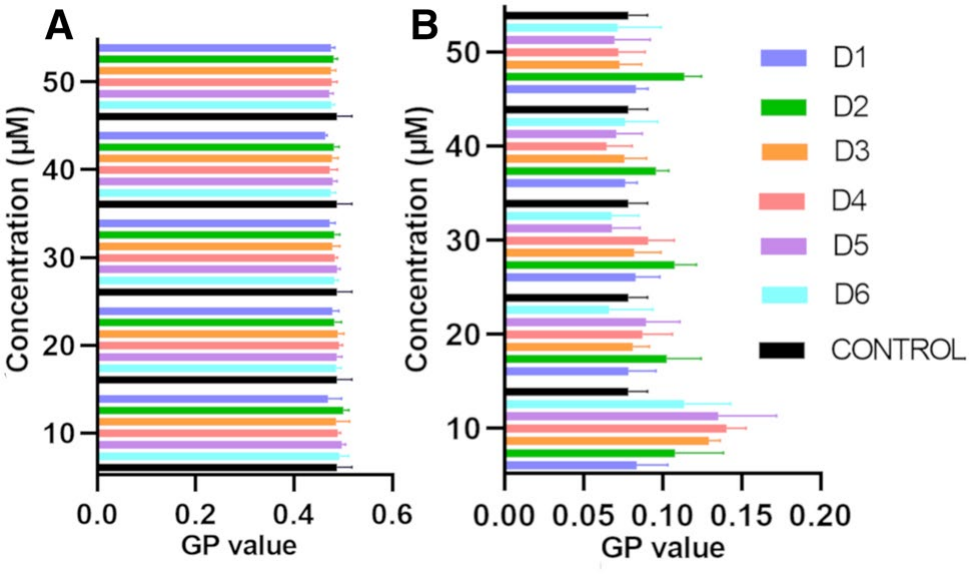
General polarization values (GP) of Laurdan probe for (**A**) MODEL and (**B**) MIMIC membranes in the presence of compounds. Error bars represent mean ± SD (n = 4).

Changes in GP (General Polarization) are associated with changes in the polarity of the membrane environment. The changes in this indicator for the two types of membranes in the presence of the tested compounds are shown in the Fig. 3. In the case of the MIMIC membrane, when tested at the lowest concentration of 10 μM, all compounds demonstrated an increase in GP compared to the control. Compound D6 exhibited lower affinity for outer membrane layers compared to D3 or D4, which supports the hypothesis that lipophilicity plays a role in these interactions. D3 and D4, having lower lipophilic profiles, were more likely to interact with the inner, more hydrophilic parts of the lipids.

As evidence of the reliability of the studies based on observations of the behaviour of fluorescent probes, we examined whether the addition of any of the investigated compounds would lead to quenching of the fluorescence of the DPH and Laurdan probes. The Stern–Volmer plots showing the concentration dependence of the fluorescence intensity ratio, which represents the degree of extinction are provided in Supplementary Materials (Fig. S1). The highest value (3.2) of F_0_/F was recorded for D2 in the concentration of 50 μM when the values for the other compounds did not exceed 2.5. This range of F_0_/F ratio values can be considered as not a quenching the probe^18,19^.

### Structural characteristics of halogenated flavonoid derivatives with lipid membranes: a spectroscopic study

**Figure 4:**
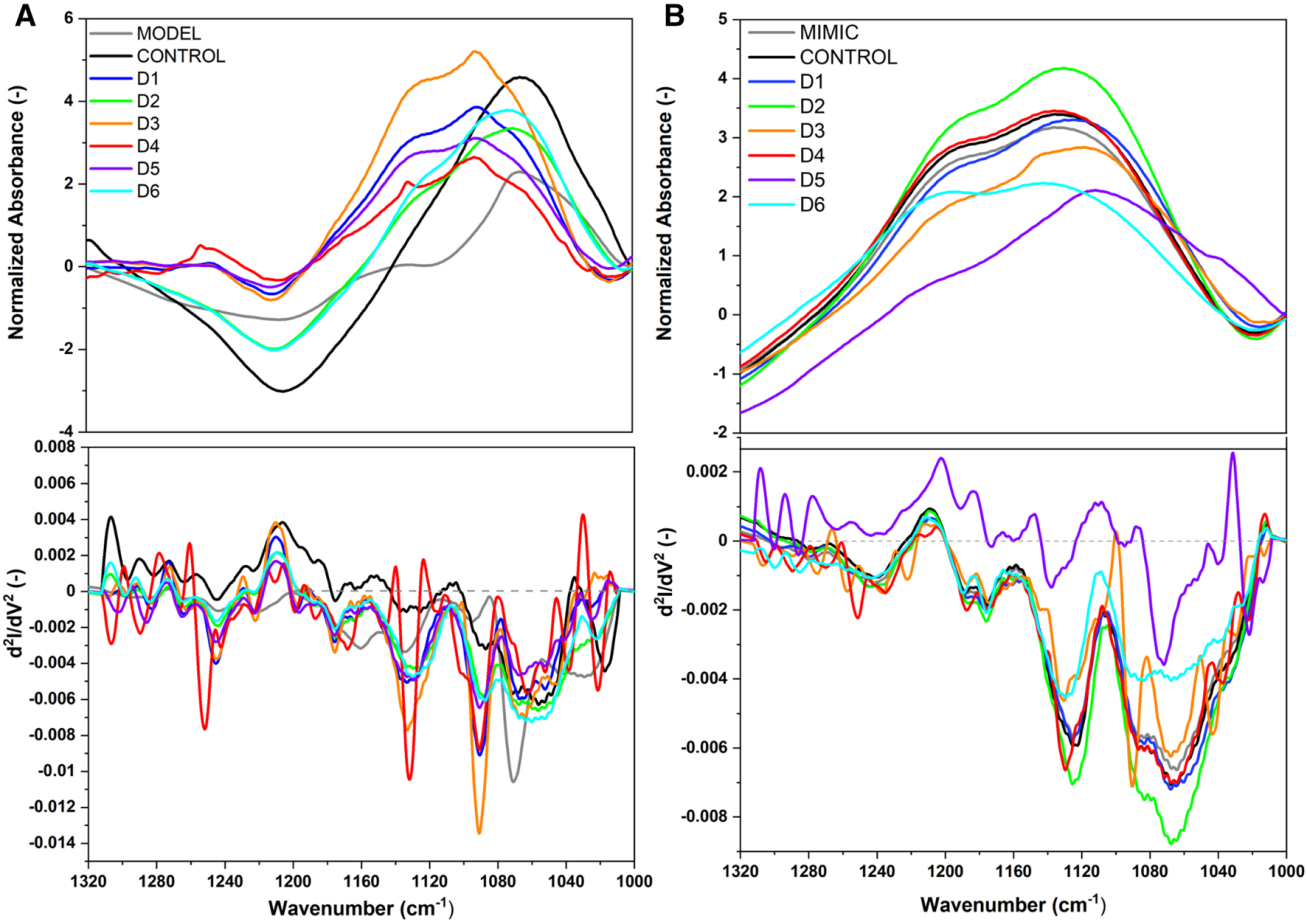
Normalized ATR-FTIR spectra of D1 to D6 compounds on MODEL (**A**) and MIMIC (**B**) lipid membranes in the range of 1320–1000 cm^−1^ with second derivatives, directly after dissolving. Spectra normalized to the CH_2_ group (1450 cm^−1^).

We explored the interaction of D1–D6 halogenated flavonoid derivatives with lipid membranes using techniques such as Attenuated Total Reflectance Fourier Transform Infrared Spectroscopy (ATR-FTIR) and Fourier Transform Raman Spectroscopy (FT-Raman). These studies focused on aspects such as lipid conformation and the structural orientation of halogenated flavonoid derivatives to MODEL and MIMIC lipid membranes. First, the spectroscopic characteristics of halogenated flavonoid derivatives were obtained. A characteristic fingerprint region of the halogenated flavonoid ATR-FTIR spectra, in the range of 1300–400 cm^−1^ (see Fig. S2), was identified for further analyses. The introduction of halogens (Cl, Br) into the chemical structure induced shifts in the ATR-FTIR powder bands corresponding to the C–Cl and C–Br stretches (see Fig. S3), contrasting with the typical spectra of flavones^20^. Thus, halogens, being more electronegative than hydrogen, result in more intense absorption bands arising from the vibrations of the studied groups^21,22^. Consequently, this underscores how the substitution of chlorine and bromine in flavonoid structures affects their interaction with lipid membranes. Secondly, ATR-FTIR was employed to study how the compounds D1 to D6, which are halogenated derivatives, influenced distinct areas of the membranes in different ways. Within the spectral range of 2980–2820 cm^−1^, associated with the symmetrical and asymmetrical stretching vibrations of CH_2_ and CH_3_ groups, no alterations within the lipid acyl chain environment were induced by the compounds D1–D6 in the MODEL and MIMIC membranes (see Fig. S4 and Table S1). Thus, the D1–D6 halogenated flavonoid derivatives did not interact with the hydrophobic part of the lipid membranes. In contrast, a clear interaction was observed in the lipid head groups, indicated by significant changes in the PO_2_^−^ stretching vibration region (see Fig. 4 and Table S1).

Analysis of the ATR-FTIR spectra, as presented in Fig. 4A, revealed that compounds D1, D3, and D5 interact with MODEL membranes. A significant shift in the symmetric stretching of PO_2_^−^ groups towards higher wavenumbers (approximately 1091 cm^−1^) was observed, as detailed in Table S1. This shift suggests an interaction with the PO_2_^−^ entities^23^. Conversely, compounds D2 and D6 exhibited a redshift of 1 cm^−1^ and 2 cm^−1^, respectively, indicating a weaker interaction with phosphate moieties, which can be attributed to the formation of hydrogen bonds. For the MIMIC membrane, a shift to lower wavenumbers in the asymmetric stretching of PO_2_^−^ was noted, potentially signifying interaction or binding with the phosphate groups or alterations within the lipid acyl chain environment. The asymmetric stretching frequency of the PO_2_^−^ group demonstrates high sensitivity to changes in environmental polarity and the capacity for hydrogen bond formation. In the case of compounds D1, D2, D3, D4, and D5, a shift to higher frequencies was recorded, as indicated in Table S1, suggesting interactions with the lipid membranes.

**Figure 5:**
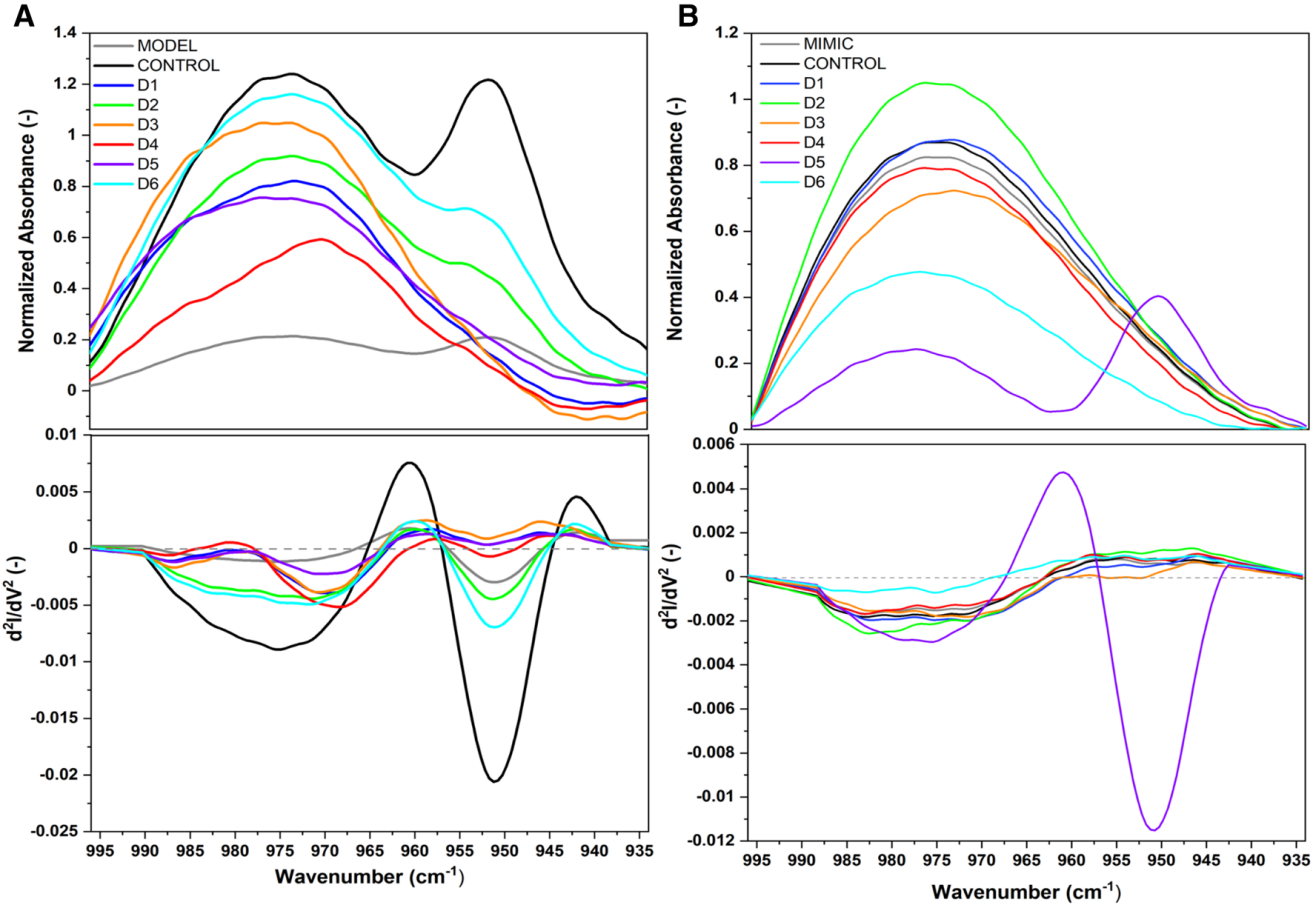
Normalized ATR-FTIR spectra of D1–D6 compounds with MODEL (**A**) and MIMIC (**B**) lipid membranes, in the range of 996 – 934 cm^−1^ with second derivatives, directly after dissolving. Spectra normalized to the CH_2_ group (1450 cm^−1^).

Furthermore, within the choline headgroup region (Fig. 5), a subtle modification in the vibrational frequencies of the asymmetric N-(CH_3_)_3_ stretching modes for both MODEL and MIMIC membranes is evident (Table S1). For MODEL membranes, all compounds demonstrated a shift toward lower wavenumbers relative to the control. Conversely, in MIMIC membranes, only compounds D3, D5, and D6 exhibited a red shift. This observation suggests differential interactions of the compounds with the choline headgroups in the two membrane models.

**Figure 6:**
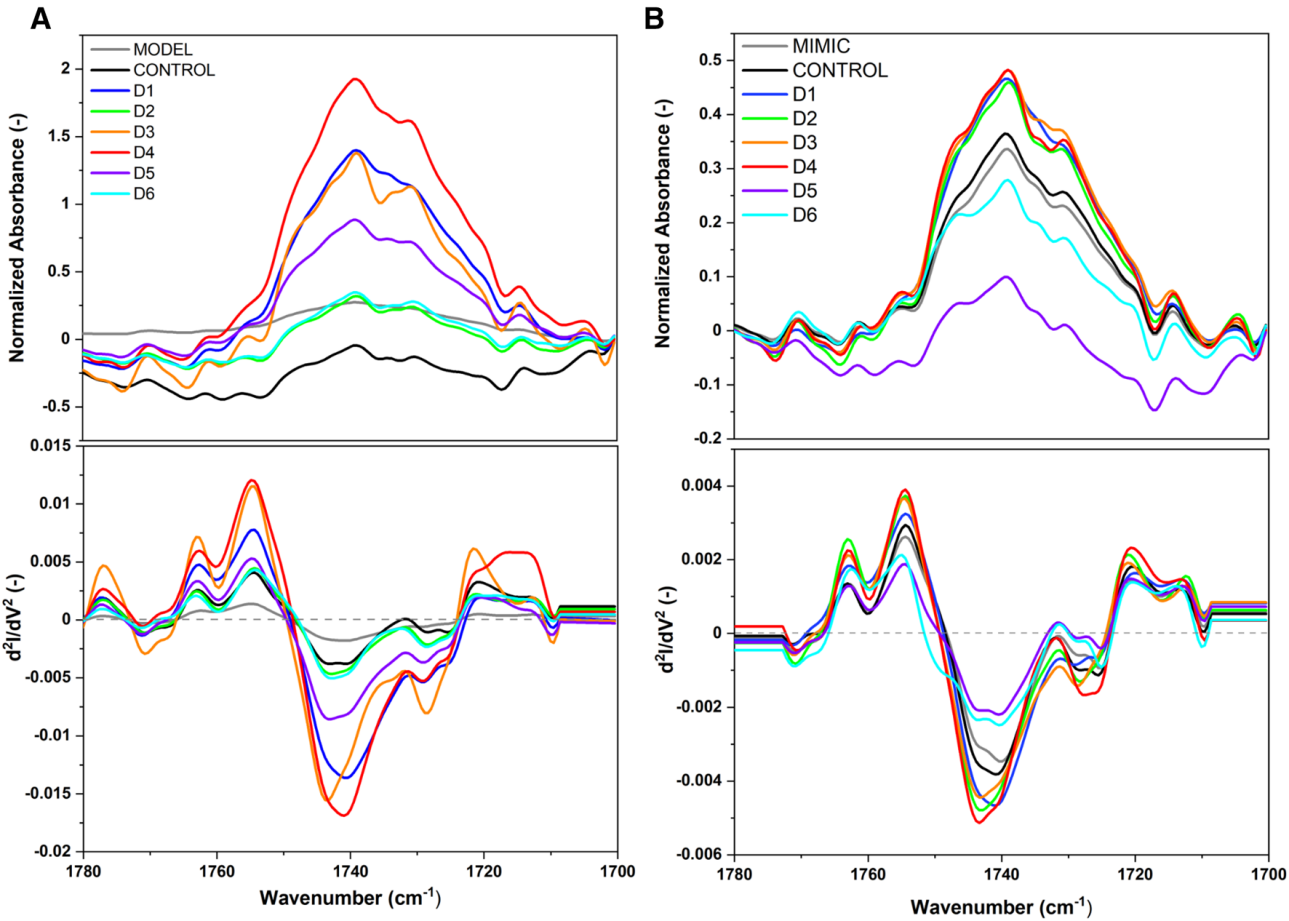
Normalized ATR-FTIR spectra of D1–D6 compounds on MODEL (**A**) and MIMIC (**B**) lipid membranes in the range of 1780–1700 cm^−1^ with second derivatives, directly after dissolving. Spectra normalized to the CH_2_ group (1450 cm^−1^).

Additionally, the evident interaction of halogenated flavonoid derivatives with the ester carbonyl (C=O stretching vibration) groups of lipids (1780–1700 cm^−1^) can be observed (see Fig. 6 and Table S1). For the MODEL system, the control shows the ν(C = O) bond stretching vibration at 1744 cm^−1^ (Table S1), indicative of nonhydrated ester carbonyl groups, and a free ν(C=O) stretching vibration at 1725 cm^−1^, corresponding to hydrated ester carbonyl groups^24^. Upon interaction with the derivatives D1 to D6, a general trend of slight shifts in the absorption peaks is observed. Derivatives D1, D2, D3, and D4 exhibit a red shift in the nonhydrated ν(C=O) bond stretching vibration, suggesting a direct interaction that affects the carbonyl group's environment, potentially through H-bonding or dipole interactions. Notably, D3 and D4 also induce a blue shift in the free ν(C=O) stretching vibration, indicating changes in the hydration state or environment of the carbonyl groups. In contrast, the MIMIC system demonstrates a slightly different interaction pattern, with the control ν(C=O) bond stretching vibration observed at 1741 cm^−1^ (Table S1). The presence of diverse lipid components in the MIMIC model appears to influence the interaction, as evidenced by the upward shifts in the ν(C=O) bond stretching vibration for D2, D3, and D4, unlike the downward or neutral shifts observed in the MODEL system. This suggests that the more complex composition of the MIMIC model, particularly the inclusion of different phospholipids and cholesterol, modulates the interaction dynamics between the ester carbonyl groups and the halogenated flavonoids.

**Figure 7:**
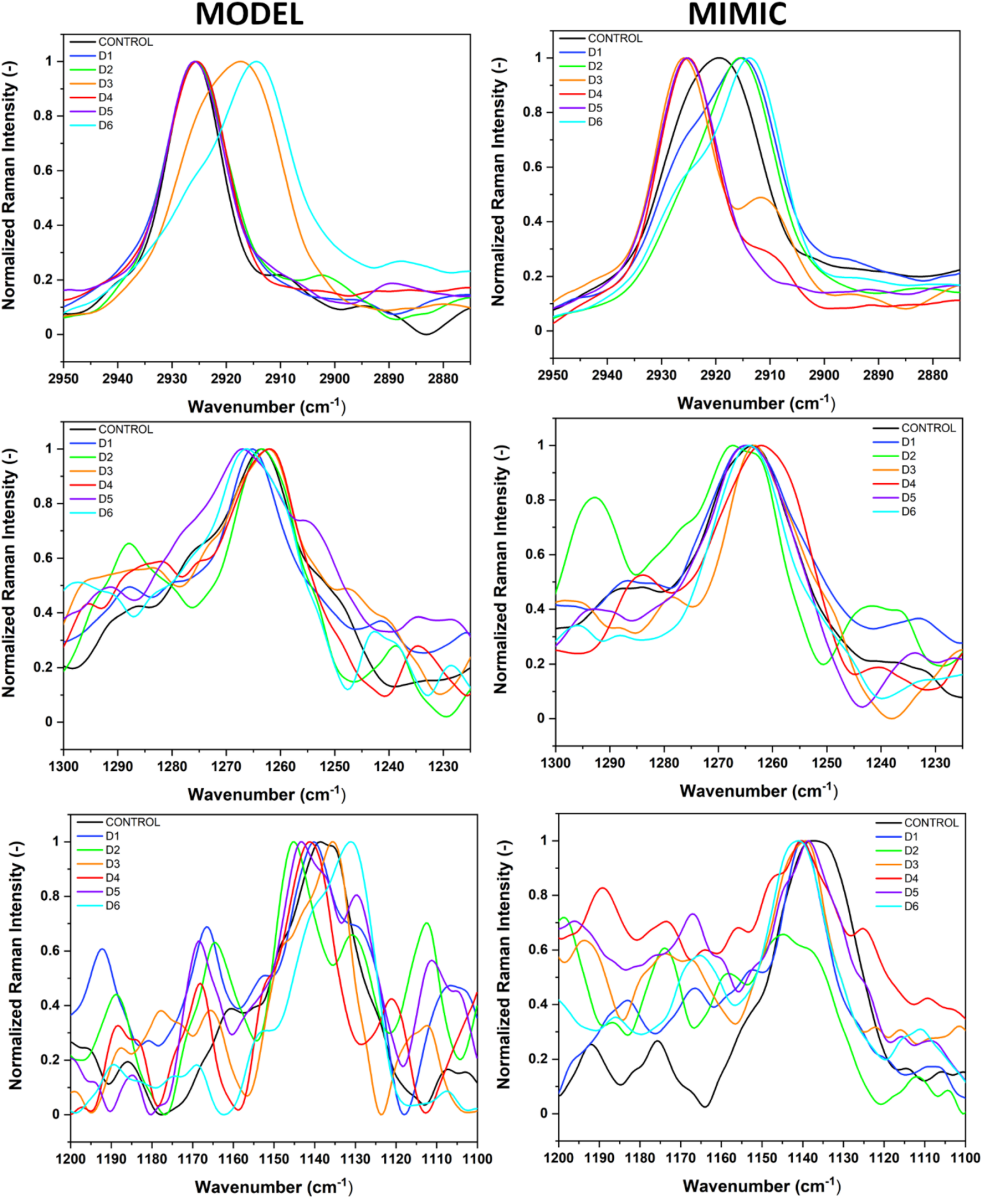
Normalized FT-Raman spectra of D1–D6 compounds with MODEL (left column) and MIMIC (right column) lipid membranes, smoothed with SG 35 (see Methods), in the wavenumber range of: 2950–2875 cm^−1^, 1300–1225 cm^−1^ and 1200–1100 cm^−1^. Spectra normalized to [0 1] in the analysed regions.

Additionally, the second vibrational technique, FT-Raman, provides additional information about the effects of D1–D6 compounds on the MODEL and MIMIC lipid membranes within their hydrophobic region^25^. In FT-Raman spectra, we focused on the three significant areas of the fatty acyl chains, complemented by several spectral bands originating from the head groups: the C–H stretching region (3000–2800 cm^−1^), the deformation regions of CH_3_ and CH_2_ (1400–1200 cm^−1^), and the C–C stretching region (1200–1000 cm^−1^) (see Fig. S5 and Table S2). For the MODEL lipid membranes, the introduction of halogenated flavonoids derivatives (D1–D6) caused subtle variations in the C–H stretching regions. Notably, compounds D2 and D6 induced a slight decrease (see Fig. 7 and Table S2) in the asymmetric CH_3_ stretching frequencies (from 2955 to 2947  cm^−1^ and 2948 cm^−1^, respectively), suggesting modifications in the membrane's hydrophobic core, possibly due to alterations in lipid packing or fluidity.

The asymmetric and symmetric CH_2_ stretching frequencies exhibited by D2 and D6 (2888 cm^−1^) also suggest an increased disorder in the acyl chain region, indicating that these compounds may induce a fluidizing effect^26^ on the MODEL membranes. Interestingly, the deformation regions of CH and C–C stretching also showed variations upon the addition of flavonoid derivatives. For instance, D5 and D6 caused an increase in the deformation of CH frequencies (1267 and 1266 cm^−1^, respectively), and D2 resulted in the highest shift in C–C stretching frequency (1145 cm^−1^). These observations hint at the specific interactions between flavonoid derivatives and lipid molecules, potentially influencing the structural dynamics and stability of the lipid bilayers^27^. In the case of MIMIC lipid membranes, the effects of the flavonoid derivatives were more pronounced. The asymmetric CH_3_ stretching frequencies decreased significantly for all derivatives (with D3 showing the lowest frequency at 2941 cm^−1^, see Fig. 7 and Table S2), indicating a considerable impact on the membrane's hydrophobic core. In the case of symmetric CH_3_ stretching, the most evident effect was observed for D6 compound with a red shift to 2914 cm^−1^, Fig. S5 and Table S2. This effect is further supported by the variations in the symmetric and asymmetric CH_2_ stretching regions, where D3 and D6 induced notable shifts (Table S2), suggesting alterations in membrane fluidity and lipid packing. Moreover, the specific interactions between flavonoid derivatives and the MIMIC membranes are evident from the deformation of CH and C–C stretching frequencies. The significant shift in the C–C stretching mode observed for D3 (1339 cm^−1^) underscores the potential of certain flavonoid derivatives to interact strongly with the lipid components, possibly affecting membrane properties such as permeability and rigidity.

### Determination of the mean diameter, size, polydispersity and zeta potential of liposomes in the presence of flavonoids

To gain insights into the physicochemical interactions between flavonoids and two liposomal membranes (MODEL and MIMIC), we conducted measurements on alterations in liposome size in nanoscale, zeta potential (ζ potential) and polydispersity index (PDI) using the dynamic light scattering method (DLS) (Table 2). The influence of flavonoids on membrane characteristics was notable, reflecting the lipophilic properties and charge of the flavonoid as well as the charge of the lipids that form the membrane. Analysing the size of liposomes under the effect of flavonoids, we have observed both an increase and a decrease in a vesicle size. The larger size is related to the coverage of the liposomes surface by the compound, while the decrease implies that the nanoparticles become enclosed within the lipid membranes through internalisation.

**Table 2:** Size, PDI index, and zeta potential of the MIMIC and MODEL membranes in the presence of the studied halogenated flavonoid derivatives.

	Size (nm)	PDI	ζ potential (mV)
MIMIC			
Control	116.80 ± 2.91	0.29 ± 0.05	− 11.91 ± 1.27
D1	88.52 ± 1.21	0.33 ± 0.05	− 11.88 ± 1.02
D2	104.5 ± 1.91	0.28 ± 0.07	− 13.08 ± 0.59
D3	117.18 ± 1.44	0.32 ± 0.05	− 12.71 ± 0.54
D4	109.73 ± 2.53	0.26 ± 0.01	− 12.18 ± 1.13
D5	105.02 ± 1.33	0.40 ± 0.03	− 12.86 ± 1.09
D6	233.75 ± 15.08	0.33 ± 0.03	− 12.43 ± 0.98
Model			
Control	195.51 ± 3.82	0.35 ± 0.03	− 0.28 ± 0.12
D1	195.27 ± 7.01	0.38 ± 0.03	− 0.17 ± 0.06
D2	200.8 ± 2.97	0.41 ± 0.06	− 0.18 ± 0.10
D3	229.7 ± 6.65	0.42 ± 0.08	0.80 ± 0.11
D4	239.48 ± 9.09	0.45 ± 0.02	0.31 ± 0.04
D5	177.86 ± 15.85	0.65 ± 0.08	0.50 ± 0.04
D6	239.83 ± 3.40	0.50 ± 0.06	0.38 ± 0.09

The incorporation of compound D6 into both MIMIC and MODEL membranes resulted in an increase in liposome diameter. This increase is attributed to D6’s high lipophilicity and affinity for the membrane. Similarly, compounds D3 and D4 also caused an increase in the liposome size in the MODEL membrane. Upon introducing (D1–D6) compounds into the MIMIC membrane, the surface charge of liposomes remained relatively unchanged. The low zeta potential of the MIMIC membrane, compared with the MODEL membrane, may be mainly associated with the negatively charged lipids forming the MIMIC membrane and not necessarily with the effect of the flavonoids. In the analysis of the Zeta Potential values for the MODEL liposome, it is evident that DPPC/CHOL liposomes exhibit a slightly negative surface charge, despite zwitterionic phospholipids having a neutral net polar head charge. The introduction of compounds D3, D4, D5 and D6 noticeably increased the surface charge of the MODEL liposomes, indicating an electrostatic interaction between the lipids and these negatively charged compounds. A slight increase in the PDI was observed in MIMIC liposomes with compound D5, compared to the control sample. Additionally, in the MODEL membranes, the PDI was elevated under the influence of all studied compounds, suggesting a broader size distribution. This observation indicates that the liposomes in the sample vary significantly in size or may even aggregate. Flavonoids have a more pronounced effect on the size of the MIMIC membrane than on the MODEL membrane. Flavonoids increase the PDI in both membranes, indicating increased particle size heterogeneity. Halogenated flavonoid derivatives have a relatively consistent effect on the MIMIC membrane's zeta potential, while in the MODEL membrane, some flavonoids shift the potential from near-neutral to positive. This analysis shows that the presence of different flavonoids can significantly affect the physical properties of both MIMIC and MODEL membranes, suggesting potential impacts on their stability, aggregation behaviour, and possibly their biological interactions.

### Cytotoxicity towards cancer and normal cell lines

We evaluated the IC_50_ value by treating four canine cancers (CLBL-1, GL-1, RDSVS-TCC1, K9NK) and one normal canine cell line (CF2TH) with halogenated flavonoid derivatives compounds (Table 3). The results showed that whether the cell lines were sensitive or resistant to the presence of the compounds depended largely on the type of flavonoid. However, all compounds tested were able to inhibit the proliferation of both cancer and normal cells.

**Table 3:** Comparison of concentrations (µM) of compounds that inhibited 50% of the cell viability (IC_50_ values) in CLBL-1, GL-1, RDSVS-TCC1, K9NK and CF2TH cells after 72 h of exposure.

Cell line	D1	D2	D3	D4	D5	D6
IC_50_ values after 72 h (µM)						
RDSVS-TCC1	32.65 ± 8.04	31.63 ± 5.97	28.20 ± 5.62	58.72 ± 13.29	42.96 ± 11.31	37.70 ± 14.42
K9NK	59.85 ± 14.69	31.18 ± 6.87	19.44 ± 6.75	35.60 ± 3.41	30.50 ± 13.80	29.06 ± 16.20
GL-1	26.53 ± 3.05	26.46 ± 4.57	35.15 ± 6.17	62.39 ± 2.89	38.84 ± 6.48	47.19 ± 1.41
CLBL-1	26.58 ± 3.61	26.30 ± 6.33	32.75 ± 2.11	74.37 ± 28.75	76.38 ± 23.17	38.07 ± 7.81
CF2TH*	28.16 ± 6.60	22.93 ± 6.88	28.39 ± 5.02	80.88 ± 14.08	28.52 ± 17.99	38.07 ± 7.87

Values are expressed as means ± SD of at least three independent experiments. The normal cell line has been marked with an asterisk.

The highest IC_50_ values were generally observed for compound D4, with the small exception of IC_50_ values when applied to K9NK cells, and for compound D5 against CLBL-1. Therefore, D4 may be considered as a less effective cytotoxic agent. A similar IC_50_ value of approximately 30 µM was found for compounds D1, D2, D3 for all lines tested except K9NK, which suggests their high cytotoxicity. In addition, cytotoxic effects were assessed in a non-cancerous canine cell line and showed that in this cell line as well, compounds D1, D2, D3 and D5 induced marked cell death. The similar IC_50_ values of compounds D1, D2 and D3, which differ only in the position of attachment of the chlorine atom to the B rings, may be explained by their analogous skeleton. Compound D4's cytotoxic properties decreased upon substitution of a chlorine atom at position 6 in the ring A, indicating that this position is not favoured for cytotoxic action for the majority of cell lines.

In summary, the data indicates variable responses of different cell lines to the halogenated flavonoid derivatives. Compounds D3 and D2 generally exhibit higher efficacy across most cell lines, while D4 tends to be less effective in inhibiting cell viability. It's important to note the variability in response between different cell lines, highlighting the specificity of these compounds' actions.

### Toxicity towards red blood cells (RBCs)

The cytotoxicity level of the compounds was assessed through a haemolytic activity test, measuring the impact of six compounds at concentrations of 10–50 μM on human red blood cells (RBCs). According to toxicity categorization, compounds are considered highly toxic if they cause a haemolysis rate of 90–100% and are considered non-toxic if the haemolysis rate falls within the range of 0–9%^28^.

**Figure 8:**
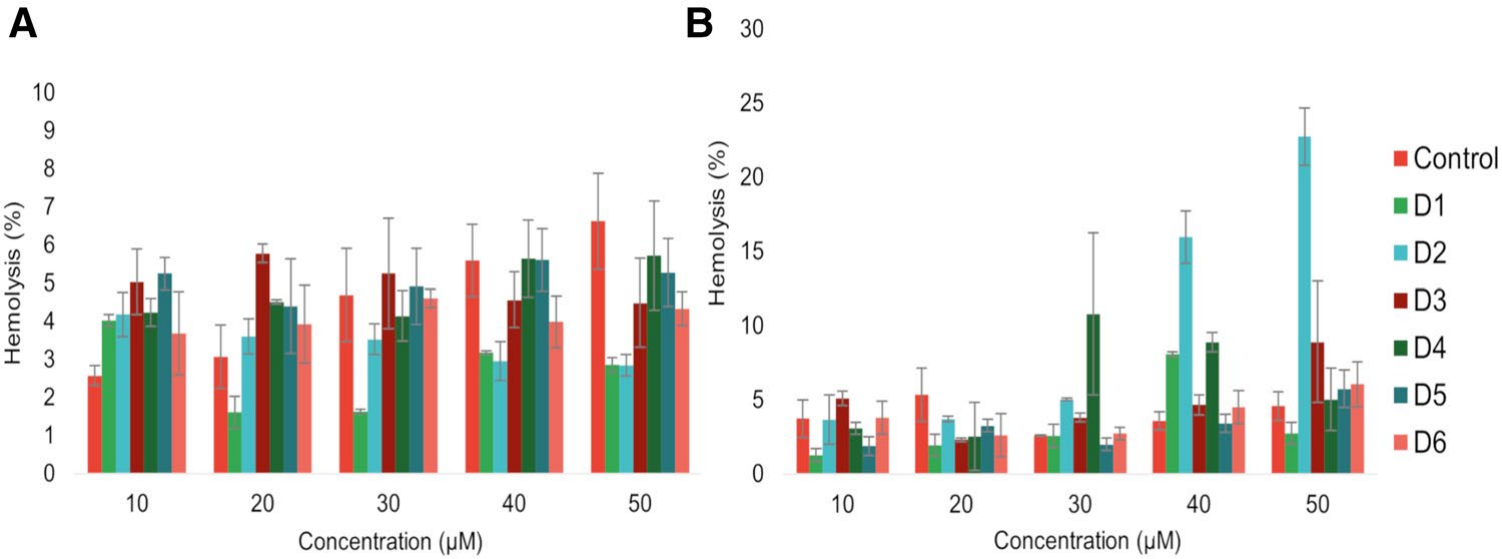
Percentage of hemolysis after 1 h (**A**) and 24 h (**B**) in the presence of the compounds. Error bars represent mean ± SD (n = 3).

After 1 h of incubation at 37 °C, all compounds at various concentrations do not exhibit cytotoxicity towards RBCs, and the hemolysis rate does not exceed 9% (Fig. 8). In a control sample containing an appropriate amount of DMSO instead of the compound, an increase in the percentage of haemolysis with increasing DMSO concentration can be observed. None of the compounds tested hemolysed to a greater extent than shown in the control. After 24 h incubation, D2 at 40 and 50 μM and D4 at 30 μM showed a higher degree of haemolysis, reaching 15.96 ± 1.78%, 22.74 ± 1.98%, and 10.79 ± 5.46%, respectively. However, these values are not representative of the potential degradation of RBCs under the influence of compounds due to specific experimental conditions.

## Discussion

In this study, we combined various complementary techniques to elucidate the potential effects of new halogenated flavonoid derivatives on lipid membranes and cells with the aim of highlighting their promising activities. To obtain preliminary information about the physicochemical properties and the potential mode of interaction of the compounds studied, we carried out a prediction of their characteristics based on SwissADME. A general guide for good oral bioavailability, which encompasses both permeability and solubility, is to have a moderate logP value, typically between 0 and 3^16^. This physicochemical characteristic is crucial in influencing interactions with membranes^29^. It is established that greater hydrophobicity, as indicated by higher logP values, enables flavonoids to penetrate membranes more effectively. However, all derivatives possess high logP values, suggesting they are lipophilic and hydrophobic, which may increase membrane permeability but could challenge aqueous solubility. Furthermore, the pharmacokinetics and drug-likeness analysis using the same tool indicated that all compounds have a high gastrointestinal absorption and a Bioavailability Score of 0.55, signifying a moderate probability of bioavailability after oral administration in rats or measurable permeability in Caco-2 cells^30^.

Our study revealed the ability of interaction of D1-D6 compounds with lipid membranes consisting of different lipids. These interactions occur mainly at the level of membrane surface layers, in the interphase between the aqueous phase and the lipid heads, which is confirmed by the ATR-FTIR. However, tracking the behaviour of the DPH probe in the deeper layers of the membrane suggests that the compounds are able to penetrate deeper into the bilayer and interact with lipid chains. This finding was confirmed by FT-Raman demonstrating the structural changes of lipid chains under the influence of compounds. It should be noted that the compounds were dissolved in DMSO, which can also affect the structure of the lipid bilayers through increased membrane penetration^31^. Interactions between DMSO and lipids have been recorded on ATR-FTIR spectra (Fig. 7) However, the concentration of DMSO in the sample was less than 1%. Villarroel et al. suggested that DMSO in concentrations of 1% or less does not have a significant inhibitory effect on cell viability and proliferation in tested cancer cells^32^. This indicates that low concentrations of DMSO might be harmless in certain biological contexts.

Various experimental methods have shown that flavonoids can be incorporated into both polar and nonpolar regions of lipid bilayers. Studies have revealed that numerous polyphenols, including quercetin, kaempferol, luteolin, and apigenin, display a high affinity for liposomal membranes^33–35^. According to the findings of Arora et al.^36^, genistein intercalates into the hydrophobic core, resulting in reduced lipid fluidity in that particular region of the membrane. The researchers used fluorescent probes to track alterations in membrane fluidity at different depths. They observed the most significant restriction in membrane mobility within the inner region. In the class of prenylated flavonoids, closely related to halogenated flavonoids, the prenyl moiety has been observed to increase their lipophilicity, resulting in an increased affinity for cell membranes^37^. This suggests that modifications aimed at increasing lipophilicity, such as halogenation, may similarly affect membrane affinity within flavonoids.

The formation of hydrogen bonds linking flavonoids' hydroxyl group to lipids is often highlighted as a major interaction mechanism with the membrane^38^. Based on the ATR-FTIR spectra, we observed the changes in the ester carbonyl (C=O stretching vibration) groups of lipids (1780–1700 cm^−1^), suggesting the possible contact with halogenated flavonoids through hydrophobic interactions.

In the case of halogenated flavonoids a different mechanism can be involved in the interactions with membranes, which may be specified for this particular type of compounds and requires further research and the use of more specific measurement methods. Some research has suggested that the halogen bond plays an important role in mediating recognition phenomena and in the distribution of halogenated compounds along phospholipid membranes^39^*.* The molecular dynamics simulations revealed positive interactions between halobenzene derivatives and phosphate or ester oxygen receptors within a simulated phospholipid bilayer. This finding supports the idea of halogen bond-mediated recognition between phospholipids and halogenated compounds. Furthermore, the research hinted at the possibility of halogen bonds facilitating the insertion of halogenated molecules from water into the membrane. The mechanism of action of the studied halogenated flavonoids was considerably influenced by the type of membrane-building lipids.

**Figure 9:**
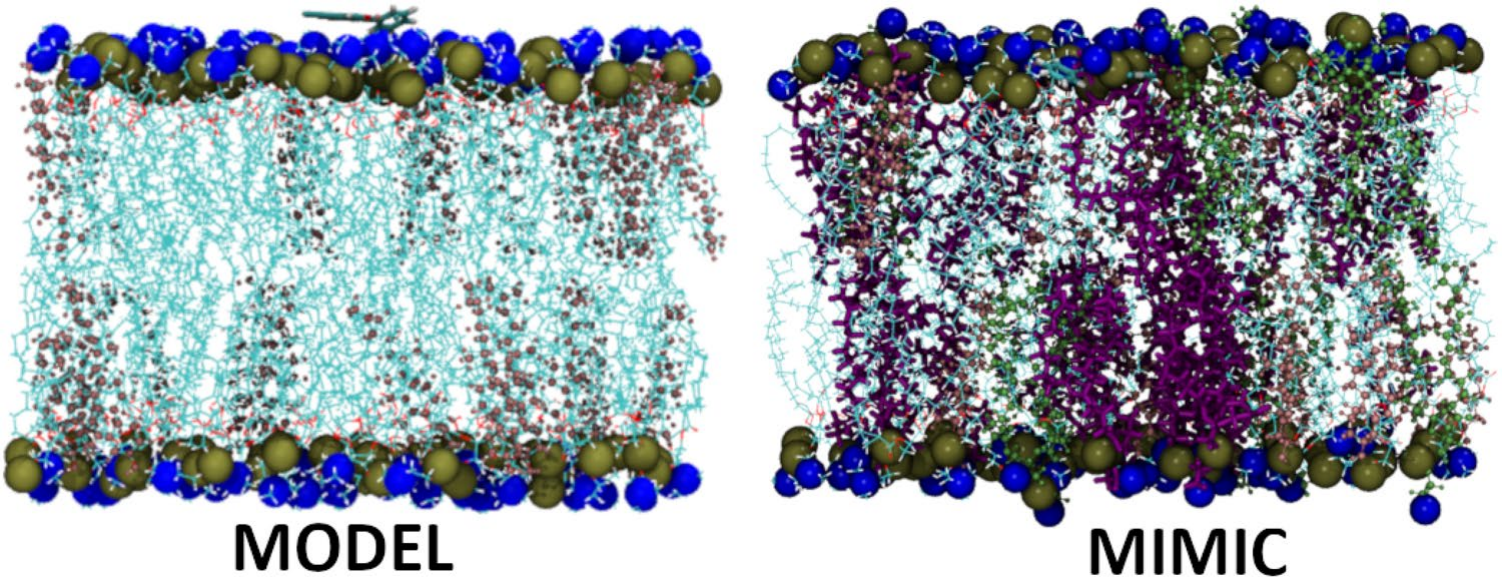
Heuristic diagram of the arrangement of D6 compounds in MODEL and MIMIC lipid membranes. The lipid head groups phosphorus P (gold) and nitrogen N (blue) are drawn as small spheres. The acyl chains of the lipids are in blue, line representation, cholesterol in CPK representation, in pink. POPE is drawn in licorice representation, in purple and SOPE in CPK representation, in lime color, compound D6 in licorice representation. Water is omitted for clarity.

In the case of MODEL and MIMIC membranes, the interaction mechanism of D1–D6 halogenated derivatives may be in a multi-faceted manner, due to the lipid composition and structural modifications of compounds. The infrared spectroscopy as well as the FT-Raman technique confirmed the distinctive interactions. That interaction could likely occur at both the surface and deeper into the lipid bilayer. However, a more pronounced effect was observed for MIMIC membranes in which the compounds influenced membrane fluidity and packing order (see Fig. 7). We observed significant changes in the CH_3_ frequencies of the studied halogenated derivatives with respect to the control sample. The shift in the CH_3_ region reflects conformational order and interchain coupling^24^, which means that the chains would exhibit increased mobility^40^. Based on that, the halogenated derivatives can more easily penetrate into the hydrophobic part of the lipid membrane. The heuristic diagram in Fig. 9 shows the possible orientation of the D6 compound within the lipid membrane. The D6 compound, with its specific halogenation pattern, could interact with lipid membranes by inserting into the lipid bilayer, potentially interacting with the hydrophobic tails of lipids due to its hydrophobic halogenated region. This interaction could affect the membrane's fluidity and permeability. Furthermore, the presence of functional groups in the D6 compound might enable hydrogen bonding or electrostatic interactions with the polar head groups of lipids, influencing the membrane's structural integrity and dynamics. The ATR-FTIR and FT- Raman spectroscopy data support the notion that D6 can integrate into the membrane, influencing both the surface layers and the deeper hydrophobic core. This dual interaction mode highlights the potential of D6 as a bioactive molecule with specific affinities for membrane components. The molecular dynamics simulations in a future study will also support the interaction of studied compounds with lipid membranes.

Liposome size analysis using DLS confirmed the interaction between the halogenated flavonoids and the surface membrane layers. Compounds, and in particular D6 increased the size of the liposomes, thus indicating an accumulation of compound molecules on the surface. An increase in the PDI index may also indicate liposome aggregation and non-specific reactions occurring between the hydrophilic part of the bilayer and the electronegative compound. In studies concerning the encapsulation of liposomes, authors demonstrated the increase in the diameter of liposomes with increasing concentration of added flavonoids and suggested that this phenomenon could be correlated with the spanning orientation of flavonoids and therefore with the expansion of the liposome volume^41^. The same research group noted that as the diameter increases, there is a corresponding rise in the PDI index. This results in a broader size distribution and a more heterogeneous suspension. Investigations focusing on fisetin encapsulation have shown an increased PDI compared to empty liposomes, which can be explained by a reduction in lipid cohesion, leading to polydispersion of the formulations when fisetin is introduced into the liposome^42^.

Similarly, as we have shown (see Table 2), the parameters characterising liposomes vary considerably depending on the lipids used to form the liposomes. Bonechi et.al^43^ demonstrated that the incorporation of quercitin into liposomes caused changes in the zeta potential of negatively charged liposomes (DOPE/DOPA) and similar behaviour was shown in rutin-loaded liposomes, changing the zeta potential towards more negative values. Both polyphenolic compounds seem to have significantly reduced the negative surface charge of DOPE/DOPA liposomes, which can be explained by their negative charge.

In order to investigate the behaviour of the halogenated flavonoids in more complex systems, their cytotoxicity was tested against cell lines and human erythrocytes.

The cytotoxicity of halogenated flavonoids derivatives is highlighted in the literature and indicates high inhibitory properties on the metabolism of various cell lines. We have shown that all derivatives tested have cytotoxic properties against cancer and normal cells. The extent to which they are able to decrease the cell viability strongly depends on the behaviour of the compound and the cell line. Kajiya et al*.*^7^ investigated the impact of lipophilicity, directly associated with the hydroxyl groups on ring B of the flavonoid structure, on the toxicity of flavonols and found that the cytotoxicity against Chinese hamster lung fibroblast V79 cells correlated with the lipophilicity order of these compounds, except for myricetin. Despite being the least lipophilic, myricetin exhibited the strongest cytotoxic effect. Such a correlation was not observed in our study because least cytotoxic effects were noted for D4 compounds.

The substitution of a bromine atom for baicalin (8-bromobaicalei) showed the highest cytotoxic effect against breast cancer cells MCF-7 among other derivatives and the IC_50_ was 10 ± 3 μM^11^. The newly established class of c 1,2,3-triazole linked flavonols hybrids were found to be strong cytotoxic agents against human colon and ovarian carcinoma with IC_50_ values < 3.0 μM^44^. On the other hand, another research showed that bromo- and chloroflavons have not shown an antiproliferative effect in HUVEC cells but have demonstrated noticeable antiangiogenic effects^10^.

The non-toxic effect of halogenated derivatives towards red blood cells obtained in our research is in agreement with other authors investigating the cytotoxicity of chalcone derivatives with bromine and chlorine atoms. They showed that the chalcone derivatives did not display haemolytic potential, and the results obtained were concentration-dependent^45^.

## Conclusions

The study's comprehensive approach combining experimental and in silico methods provided novel insights into the interaction of halogenated flavonoid derivatives with lipid membranes and their cytotoxic properties. Halogenation improved flavonoids' membrane interaction and pharmacokinetic properties, showing potential as anti-cancer agents with minimal erythrocyte toxicity. It highlighted the role of halogenation in enhancing membrane interactions, potentially affecting therapeutic efficacy.

Future research should focus on elucidating the specific molecular mechanisms underlying these interactions and the in silico efficacy and safety of these compounds to further assess their therapeutic potential.

## Materials and methods

### Chemical synthesis

The flavones, 2′-chloroflavone (D1), 3′-chloroflavone (D2), 4′-chloroflavone (D3), 6-chloroflavone (D4), 6,8-dichloroflavone (D5), and 8-bromo-6-chloroflavone (D6) were synthesized according to the reaction presented in Fig. 1. The first stage involved synthesizing chalcones through the Claisen–Schmidt condensation from appropriately substituted acetophenone (Sigma-Aldrich, St. Louis, MO, USA) with benzaldehyde (Sigma-Aldrich, St. Louis, MO, USA), as shown in Fig. 1. Substrates for synthesis were dissolved in methanol under alkaline conditions (NaOH) with the addition of water. The reaction proceeded for 3 h at the boiling point under reflux. Next, the flavones (D1–D6) were obtained by reacting appropriate chalcone with iodine in dimethyl sulfoxide (DMSO) for 3 h under reflux at 125 °C in an oil bath. Structural product analyses were conducted via Nuclear Magnetic Resonance (NMR) (^1^H-NMR, ^13^C-NMR, COSY, HSQC, HMBC) using a DRX Avance™ 600 MHz NMR spectrometer (Bruker, Billerica, MA, USA). As a solvent, deuterated acetone was used. The detailed data and method were presented in our previous paper^15^.

*2′-chloroflavone* (D1). Light-yellow crystals, ESIMS m/z 257.0 ([M+H]^+^, C_15_H_9_ClO_2_, mp = 109–110 °C, tR = 16.30, ^1^H NMR (acetone-d_6_) δ (ppm): 8.16 (1H, dd, *J* = 7.9, 1.6 Hz, H-5), 7.84 (2H, m, H-7, H-6′), 7.64 (3H, m, H-3′, H-8, H-4′), 7.57 (1H, td, *J* = 7.5, 1.3 Hz, H-5′), 7.53 (1H, m, H-6), 6.56 (1H, s, H-3); ^13^C NMR (acetone-d_6_) δ (ppm): 177.6 (C-4), 163.5 (C-2), 157.5 (C-8a), 135.1 (C-7), 133.2 (C-4′), 133.0 (C-1′, C-2′), 132.0 (C-6′), 131.4 (C-3′), 128.5 (C-5′), 126.4 (C-6), 126.1 (C-5), 124.7 (C-4a), 119.3 (C-8), 113.4 (C-3). Supporting Information: Figs. S8–S21.

*3′-chloroflavone* (D2). Light-yellow crystals, ESIMS m/z 257.0 ([M+H]^+^, C_15_H_9_ClO_2_, mp = 121–122 °C, tR = 16.86, ^1^H NMR (acetone-d_6_) δ (ppm): 8.13 (1H, td, *J* = 1.8, 0.7 Hz, H-2′), 8.12 (1H, m, H-5), 8.06 (1H, m, H-6), 7.83 (1H, ddd, *J* = 8.6, 7.0, 1.7 Hz, H-7), 7.77 (1H, m, H-8), 7.63 (2H, m, H-4′, H-5′), 7.50 (1H, ddd, J = 8.0, 7.1, 1.1, H-6), 6.93 (1H, s, H-3); ^13^C NMR (acetone-d_6_) δ (ppm): 177.8 (C-4), 162.3 (C-2), 157.1 (C-8a), 135.6 (C-3′), 135.0 (C-7), 134.9 (C-1′), 132.2 (C-4′), 131.7 (C-5′), 127.0 (C-2′), 126.3 (C-6), 126.0 (C-5), 125.8 (C-6′), 124.8 (C-4a), 119.4 (C-8), 108.8 (C-3). Supporting Information: Fig. S22–S35.

*4′-chloroflavone* (D3). Light-yellow crystals, ESIMS m/z 257.0 ([M+H]^+^, C_15_H_9_ClO_2_, mp = 182–183 °C, tR = 16.88, ^1^H NMR (acetone-d_6_) δ (ppm): 8.12 (3H, m, H-2′, H-6′, H-5), 7.83 (1H, ddd, *J* = 8.7, 7.1, 1.7 Hz, H-7), 7.74 (1H, dd, *J* = 8.5, 1.1, H-8), 7.63 (2H, m, H-3′, H-5′), 7.50 (1H, ddd, *J* = 8.1, 7.1, 1.1 Hz, H-6), 6.89 (1H, s, H-3); ^13^C NMR (acetone-d_6_) δ (ppm): 177.9 (C-4), 162.7 (C-2), 157.1 (C-8a), 138.0 (C-4′), 135.0 (C-7), 131.6 (C-1′), 130.1 (C-3′, C-5′), 128.9 (C-2′, C-6′), 126.3 (C-6), 126.0 (C-5), 124.8 (C-4a), 119.3 (C-8), 108.2 (C-3), Supporting Information: Figs. S36–S48.

*6-chloroflavone* (D4). Light-yellow crystals, ESIMS m/z 257.0 ([M+H]^+^, C_15_H_9_ClO_2_, mp = 180–181 °C, tR = 17.10, ^1^H NMR (acetone-d_6_) δ (ppm): 8.12 (2H, m, H-2′, H-6′), 8.05 (1H, m, H-5), 7.83 (2H, m, H-7, H-8), 7.62 (3H, m, H-3′, H-4′, H5′), 6.92 (1H, s, H-3); ^13^C NMR (acetone-d_6_) δ (ppm): 176.8 (C-4), 164.3 (C-2), 155.7 (C-8a), 134.8 (C-7), 132.7 (C-4′), 132.5 (C-1′), 131.4 (C-6), 130.0 (C-3′, C-5′), 127.3 (C-2′, C-6′), 126.0 (C-4a), 125.1 (C-5), 121.7 (C-8), 107.9 (C-3). Supporting Information: Figs. S49–S62.

*6,8-dichloroflavone* (D5). Light-yellow crystals, ESIMS m/z 291.0 ([M+H]^+^, C_15_H_8_Cl_2_O_2_, mp = 166–167 °C, tR = 18.83, ^1^H NMR (acetone-d_6_) δ (ppm): 8.14 (2H, dd,* J* = 8.0, 1.5 Hz, H-2′, H-6′), 7.98 (2H, dd, *J* = 7.5, 2.5 Hz, H-5, H-7), 7,65 (3H, m, H-3′, H-4′, H-5′), 6.99 (1H, s, H-3); ^13^C NMR (acetone-d_6_) δ (ppm): 176.2 (C-4), 164.1 (C-2), 151.5 (C-8a), 134.4 (C-7), 133.0 (C-4′), 132.0 (C-1′), 131.1 (C-6), 130.2 (C-3′, C-5′), 127.4 (C-2′, C-6′), 126.8 (C-4a), 125.4 (C-8), 124.2 (C-5), 107.9 (C-3). Supporting Information: Figs. S63–S76.

*8-bromo-6-chloroflavone* (D6). Light-yellow crystals, C_15_H_8_BrClO_2_, ^1^H NMR and ^13^C NMR see^15^.

### Analysis of synthesis products

HPLC analyses of synthesis products were performed on a Dionex Ultimate 3000 instrument (Thermo Fisher Scientific, Waltham, MA, USA) with a DAD-3000 diode array detector using an analytical octadecylsilica (ODS) 2 column (4.6 mm × 250 mm, Waters, Milford, MA, USA) and pre-column. The gradient program was as follows: initial conditions—32.5% B in A, 4 min—40% B in A, 8 min—40% B in A, 10 min—45% B in A, 15 min—95% B in A, 18 min—95% B in A, 19 min—32.5% B in A, 23 min—32.5% B in A. The flow rate was 1 mL/min, the injection volume was 5 µL, and the detection wavelength was 280 nm.

NMR analyses (1H-NMR, 13C-NMR, COSY, Heteronuclear Single Quantum Correlation (HSQC), HMBC) were performed using a DRX AvanceTM 600 MHz NMR spectrometer (Bruker, Billerica, MA, USA). The prepared samples of synthesis products were dissolved in deuterated acetone.

Molecular formulas of all products were confirmed by analysis performed on the LC–MS 8045 SHIMADZU Triple Quadrupole Liquid Chromatograph Mass Spectrometer with electrospray ionization (ESI) source (Shimadzu, Kyoto, Japan), operated at 30 °C. The principal operating parameters for the LC–MS were set as follows: nebulizing gas flow: 3 L min^−1^, heating gas flow: 10 L min^−1^, interface temperature: 300 °C, drying gas flow: 10 L min^−1^, data acquisition range, m/z 100–500 Da; ionization mode: positive. Data were collected with LabSolutions version 5.97 (Shimadzu, Kyoto, Japan) software.

### Prediction of physicochemical properties

The SwissADME tool was used to evaluate basic physicochemical properties, lipophilicity, pharmacokinetic properties and drug likeness^16^. The lipophilicity descriptor: n-octanol/water partition coefficient was defined as the consensus LogP_o/w_ of the five models provided by the software. The water solubility rate was calculated based on the Delaney’s model^17^.

### Liposome preparation

Two types of liposomes were prepared by the thin-film hydration method along with the extrusion technique: liposomes mimicking the composition of cancer cell membranes (MIMIC: POPC—48%, POPE—24%, SOPS—8%, and cholesterol—20%) and liposomes formed from DPPC and 20% cholesterol (MODEL). Lipids dissolved in chloroform were evaporated under a nitrogen atmosphere and then dried in a desiccator for 120 min under vacuum. The obtained dry lipid film was hydrated in phosphate buffer, pH 7.4, obtaining a concentration of 0.1 mg/ml, and then vortexed until a homogeneous, milky suspension was observed. Lipid film hydration was performed above the main phase transition temperature of DPPC (42 °C). The mixture was sonicated using 20 kHz probe sonicator (Sonic, Milano, Italia) for 10 min providing a cooling temperature of 0 °C. The suspension was then processed (extrusion) through polycarbonate filters with pore diameters of 200 nm using a Lipex extruder (Evonik).

### Fluorometric studies

Fluorescence-based analysis was employed to investigate the lipid arrangement in MIMIC and MODEL liposomes. Laurdan and DPH fluorescence probes were used to assess the impact of flavonoids on the hydrophilic and hydrophobic parts of the liposomal membranes. Laurdan, sensitive to polarity changes, provided the General Polarization (GP) index. The anisotropy index of the DPH probe evaluated potential changes in the fluidity of the deeper layers. These indicators collectively offer insights into how flavonoids influence both the outer and deeper layers of the liposomal membrane, providing a comprehensive view of their impact on lipid organization. GP (1) and anisotropy (2) indexes were calculated according to the formulas:

1 $$A = \frac{{I_{\parallel } - GI_{ \bot } }}{{I_{\parallel } + 2GI_{ \bot } }}$$

where I_‖_ and I_⏊_ are the intensity of fluorescence in parallel and perpendicular directions to the plane of polarization of the excitation wave, respectively. G is an apparatus constant that depends on the emission wavelength^46^.

2 $$GP=\frac{{I}_{b}- {I}_{r}}{{I}_{b}+{I}_{r}}$$

where I_b_ is fluorescence intensity at λ = 440 nm and I_r_ is fluorescence intensity at λ = 490 nm^47^.

For the fluorescence assays described above, liposomes were incubated for 30 min with the appropriate probe (dissolved in DMF, 1 µM concentration). Flavonoids at concentrations of 10 µM to 50 µM (dissolved in DMSO) were added to the liposome mixture, subsequently incubated and shaken at 37˚C for 2 h using a Thermoshaker. The control sample consisted of liposomes, fluorescent probe and DMSO without the tested compounds. Fluorescence was measured on a fluorimeter (Cary Eclipse, Varian, San Diego, USA) at 37 °C. The excitation wavelength λ_exc_ and emission wavelength λ_em_ respectively: DPH – λ_exc_ = 360 nm, λ_em_ = 425 nm. nm; Laurdan – λ_exc_ = 360 nm, λ_em_ = 440 nm and 490 nm.

### Attenuated total reflectance Fourier transform infrared spectroscopy (ATR-FTIR)

Spectral data were acquired utilizing a Nicolet 6700 FTIR spectrometer (Thermo Scientific, USA), equipped with an attenuated total reflectance (ATR) feature and a temperature-controlled diamond top-plate from PIKE Technologies, under a continuous flow of dry air. For analysis, 10 μL of the liposome suspension was placed on the diamond surface, left to evaporate, and then immediately analyzed post-preparation (dry-film technique). The ATR-FTIR spectral data spanned from 3600 to 400 cm^−1^, compiling 512 interferograms per spectrum with a spectral resolution of 4 cm^−1^ (Fig. S6). Measurements were uniformly performed at a stable temperature of 37 °C. Prior to each sample analysis, a reference spectrum of the diamond surface in contact with air was captured (512 scans, 4 cm^−1^ resolution). The experiments utilized a compound concentration of 50 μM and a lipid concentration of 0.1 mg/ml to enhance the signal-to-noise ratio. Spectral analysis was conducted using OriginPro 2021 software (Origin Lab Corporation, Northampton, Massachusetts, United States), which involved steps such as automatic atmospheric interference correction, baseline correction, and normalization of the spectra against the CH_2_ stretch at 1450 cm^−1^.

### Fourier transform-Raman (FT-Raman)

Raman spectral data were collected using a Nicolet NXR 9650 FT-Raman spectrometer (Thermo Scientific, USA), which includes a MicroStage extension, a neodymium-doped yttrium orthovanadate (Nd: YVO4) laser operating at 1064 nm with a power of 100 mW for excitation, and an indium gallium arsenide (InGaAs) detector for signal detection. Each analysis involved placing a 10 μL drop of the sample liposome suspension onto a gold substrate, which was then allowed to dry via exposure to the laser. The FT-Raman spectra captured a frequency range from 3700 to 0 cm^−1^, achieving a resolution of 4 cm^−1^ by averaging over 1024 scans (Fig. S7). The study maintained a consistent concentration of the compounds at 50 μM and lipids at 0.1 mg/ml to optimize the signal-to-noise ratio. Spectral acquisition was performed immediately following the dissolution of samples. Spectral processing and analysis were conducted using OriginPro 2021 software (Origin Lab Corporation, Northampton, Massachusetts, United States), with procedures including baseline adjustment, Savitzky-Golay (SG) smoothing with a window of 35 points and a polynomial order of 2, and normalization of spectra within the [0–1] range for the specific band region under investigation.

### DLS measurements—mean diameter, size, polydispersity and zeta potential of liposomes

To the previously prepared liposomes according to procedure 2.2, the test compounds were added at a concentration of 30 μM and incubated and shaken at 37 °C for 2 h using Termoshaker. All determinations were recorded at 37 °C using a ZetasizerNano ZS (Malvern Instruments, Malvern, UK). The control sample consisted of liposomes, fluorescent probe, and DMSO without the tested compounds.

### Cytotoxicity towards cancer and normal cell lines

The study involved four canine cancer cell lines: RDSVS-TCC1 and K9NK (bladder cancer), CLBL-1 (B-cell lymphoma), GL-1 (B/T-cell leukemia) and noncancerous cell line CF2TH (fetal canine thymus). CLBL-1 was obtained from Barbara C.Ruetgen from the Institute of Immunology, Department of Pathobiology, University of Veterinary Medicine, Vienna^48^. GL-1 from Yasuhito Fujino and Hajime Tsujimoto of the University of Tokyo, Department of Veterinary Internal Medicine^49^. The CF2TH cell line was purchased from American Type Culture Collection (ATCC, Rockville, MD, USA).

K9NK cell line was generous gifts from Deepika Dhawan (Purdue University College of Veterinary Medicine, USA) and RDSVS-TCC1 was a gift from Maciej Parys (Royal (Dick) School of Veterinary Studies and The Roslin Institute, University of Edinburgh, Scotland), respectively.

The cell lines were maintained in RPMI- 1640 (CLBL-1, GL-1 and CF2TH) (Gibco, Grand Island, NY, USA) or DMEM (Institute of Immunology and Experimental Therapy, Polish Academy of Sciences, Wrocław, Poland) (RDSVS-TCC1, K9NK) culture medium supplemented with 10% fetal bovine serum FBS (Gibco, Grand Island, NY, USA), 100 units/mL penicillin and 100 mg/mL streptomycin and 1% of L-glutamine (Sigma Aldrich, Steinheim, Germany).

The cytotoxicity assay was determined by MTT test after 72 h of treatment, according to the procedure described previously^50^ with minor modifications.

In summary, the cells plated at the density of 3*10^4 (k9NK, RDSVS-TCC1), 2*10^5^ (CLBL-1, GL-1) and 7.5*10^3^ (CF2TH) per mililiter were seeded in 96-well plates (NUNC, Denmark).

The concentration of tested flavonoids was in the range 1.25–50 µM and DMSO concentration was < 1% in each dilution which is considered harmless to the cells). After incubation for 72 h, 10 µl of 3-(4,5-dimethylthiazol-2-yl)-2,5-diphenyltetrazolium bromide (MTT) solution was added to each well followed by 4 h incubation, then 40 μL of lysis buffer were added. The optical density (OD) of formazan formed from MTT was measured after 24 h using a spectrophotometric microplate reader (Spark, Tecan, Männedorf, Switzerland) at a reference wavelength of 570 nm.

The results were: determined from more than three independent experiments (three wells each) and presented as mean IC_50_ value ± SD.

### Hemolysis of red blood cells (RBCs)

Hemolysis, a method to assess the release of hemoglobin from red blood cells (RBCs), was comprehensively explained in our prior research^51^.

For erythrocytes with a hematocrit level of 12%, the compounds were introduced at concentrations of 10, 20, 40 and 60 μM. The prepared samples were kept at 37 °C for 1 and 24 h.

After the incubation period, the samples underwent centrifugation, and the hemoglobin concentration in the supernatants was quantified using a UV–VIS spectrophotometer (Specord 40, AnalytikJena) at a wavelength of 540 nm. The percentage of RBCs hemolysis were determined by comparing the absorbance of hemoglobin in the tested samples to the absorbance of hemoglobin in completely hemolyzed cells (100%). Samples achieving total hemolysis (100%) were generated by adding distilled water.

### Supplementary Information

Supplementary Information.

## Data Availability

The datasets used and/or analysed during the current study are available from the corresponding author on reasonable request.
