# Supplementary material for: Structural investigation of interactions between halogenated flavonoids and the lipid membrane along with their role as cytotoxic agents
Source: Sci Rep. 2024 May 8;14:10561. doi: 10.1038/s41598-024-61037-y (PMC11078956; doi:10.1038/s41598-024-61037-y)
Supplement: Supplementary file 1 — Supplementary Information. [file 41598_2024_61037_MOESM1_ESM.pdf]

Supplementary materials

Structural investigation of interactions between halogenated flavonoids and lipid membrane along with their role as cytotoxic agents

Anita Dudek[1*], Natalia Szulc[1], Aleksandra Pawlak[2], Paulina Strugała-Danak[1], Agnieszka Krawczyk-Łebek[3], Martyna Perz[3], Edyta Kostrzewa-Susłow[3] Hanna Pruchnik[1]

Content	Page number
**Figure S1.** Stern–Volmer plots for D1 (A), D2 (B), D3 (C), D4 (D), D5 (E), D6 (F) induced quenching of Laurdan and DPH probes. F0 and F denote the absence and the presence of a quencher, respectively. The F0/F ratio was plotted against the quencher concentration.	**5**
**Figure S2**. Raw ATR-FTIR powder spectra of D1, D2, D3, D5 and D6 compounds in the range of 3600-400 cm-1.	**6**
**Figure S3.** Raw ATR-FTIR powder spectra of D3, D5 and D6 compounds in the range of 900-500 cm-1.	**6**
**Figure S4.** Normalized ATR-FTIR spectra of D1 to D6 compounds with MODEL (A) and MIMIC (B) lipid membranes, in the range of 2980-2820 cm-1 with second derivatives, directly after dissolving. Spectra normalized to the CH2 group (1450 cm-1).	**7**
**Table S1.** Tentative assignments of bands in ATR-FTIR spectra of the studied D1-D6 halogenated flavonoid derivatives with lipid membranes (MODEL and MIMIC) in dry-film form at 37°C, where '↓' denotes a shift to a lower wavenumber compared to the control, and '↑' indicates a shift to a higher wavenumber compared to the control.	**8,9**
**Figure S5.** Normalized FT-Raman spectra of D1 to D6, MODEL, MIMIC membranes and their interactions in regions: (3000−2800 cm-1), (1400−1200 cm-1), and (1200−1000 cm-1). Spectra normalized to [0 1] in the analyzed regions.	**10**
**Table S2.** Tentative assignments of the local maxima presented in the FT-Raman spectra of the studied D1-D6 halogenated flavonoid derivatives with lipid membranes (MODEL and MIMIC) in dry-film form at room temperature, where 'sh' denotes the shoulder band. Additionally, rows correspond to the most intensive bands, which are filled in lilac color.	**11**
**Figure S6.** Raw ATR-FTIR spectra of D1 to D6, MODEL, MIMIC membranes and their interactions in the range of 3600-400 cm-1.	**12,13,14**
**Figure S7.** Raw FT-Raman spectra of D1 to D6, MODEL, MIMIC membranes and their interactions in the range of 3600-400 cm-1.	**15,16,17**
**Figure S8.** MS analysis of 2’-chloroflavone (**D1**)	**17**
**Figure S9.** HPLC analysis of 2’-chloroflavone (**D1**)	**18**
**Figure S10.** ^{1}H NMR spectrum (*δ,* acetone-d6, 600 MHz) of 2’-chloroflavone (**D1**)	**19**
**Figure S11.** ^{1}H NMR spectrum expansion (*δ,* acetone-d6, 600 MHz) of 2’-chloroflavone (**D1**)	**19**
**Figure S12.** ^{13}C NMR spectrum (*δ,* acetone-d6, 151 MHz) of 2’-chloroflavone (**D1**)	**20**
**Figure S13.** ^{13}C NMR spectrum expansion (*δ,* acetone-d6, 151 MHz) of 2’-chloroflavone (**D1**)	**20**
**Figure S14.** ^{13}C NMR spectrum expansion (*δ,* acetone-d6, 151 MHz) of 2’-chloroflavone (**D1**)	**21**
**Figure S15.** COSY contour map – ^{1}H x ^{1}H of 2’-chloroflavone (**D1**)	**21**
**Figure S16.** COSY contour map – ^{1}H x ^{1}H expansion of 2’-chloroflavone (**D1**)	**22**
**Figure S17.** HMQC contour map – ^{1}H x ^{13}C of 2’-chloroflavone (**D1**)	**22**
**Figure S18.** HMQC contour map – ^{1}H x ^{13}C expansion of 2’-chloroflavone (**D1**)	**23**
**Figure S19.** HMBC contour map – ^{1}H x ^{13}C of 2’-chloroflavone (**D1**)	**23**
**Figure S20.** HMBC contour map – ^{1}H x ^{13}C expansion of 2’-chloroflavone (**D1**)	**24**
**Figure S21.** HMBC contour map – ^{1}H x ^{13}C expansion of 2’-chloroflavone (**D1**)	**24**
**Figure S22.** MS analysis of 3’-chloroflavone (**D2**)	**25**
**Figure S23.** HPLC analysis of 3’-chloroflavone (**D2**)	**26**
**Figure S24.** ^{1}H NMR spectrum (*δ,* acetone-d6, 600 MHz) of 3’-chloroflavone (**D2**)	**26**
**Figure S25.** ^{1}H NMR spectrum expansion (*δ,* acetone-d6, 600 MHz) of 3’-chloroflavone (**D2**)	**26**
**Figure S26.** ^{13}C NMR spectrum (*δ,* acetone-d6, 151 MHz) of 3’-chloroflavone (**D2**)	**27**
**Figure S27.** ^{13}C NMR spectrum expansion (*δ,* acetone-d6, 151 MHz) of 3’-chloroflavone (**D2**)	**27**
**Figure S28.** ^{13}C NMR spectrum expansion (*δ,* acetone-d6, 151 MHz) of 3’-chloroflavone (**D2**)	**28**
**Figure S29.** COSY contour map – ^{1}H x ^{1}H of 3’-chloroflavone (**D2**)	**28**
**Figure S30.** COSY contour map – ^{1}H x ^{1}H expansion of 3’-chloroflavone (**D2**)	**29**

**Figure S31.** HMQC contour map – ^{1}H x ^{13}C of 3'-chloroflavone (**D2**)	**29**
**Figure S32.** HMQC contour map – ^{1}H x ^{13}C expansion of 3'-chloroflavone (**D2**)	**30**
**Figure S33.** HMBC contour map – ^{1}H x ^{13}C of 3'-chloroflavone (**D2**)	**30**
**Figure S34.** HMBC contour map – ^{1}H x ^{13}C expansion of 3'-chloroflavone (**D2**)	**31**
**Figure S35.** HMBC contour map – ^{1}H x ^{13}C expansion of 3'-chloroflavone (**D2**)	**31**
**Figure S36.** MS analysis of 4'-chloroflavone (**D3**)	**32**
**Figure S37.** HPLC analysis of 4'-chloroflavone (**D3**)	**32**
**Figure S38.** ^{1}H NMR spectrum (*δ,* acetone-d6, 600 MHz) of 4'-chloroflavone (**D3**)	**33**
**Figure S39.** ^{1}H NMR spectrum expansion (*δ,* acetone-d6, 600 MHz) of 4'-chloroflavone (**D3**)	**33**
**Figure S40.** ^{13}C NMR spectrum (*δ,* acetone-d6, 151 MHz) of 4'-chloroflavone (**D3**)	**34**
**Figure S41.** ^{13}C NMR spectrum expansion (*δ,* acetone-d6, 151 MHz) of 4'-chloroflavone (**D3**)	**34**
**Figure S42.** COSY contour map – ^{1}H x ^{1}H of 4'-chloroflavone (**D3**)	**35**
**Figure S43.** COSY contour map – ^{1}H x ^{1}H expansion of 4'-chloroflavone (**D3**)	**35**
**Figure S44.** HMQC contour map – ^{1}H x ^{13}C of 4'-chloroflavone (**D3**)	**36**
**Figure S45.** HMQC contour map – ^{1}H x ^{13}C expansion of 4'-chloroflavone (**D3**)	**36**
**Figure S46.** HMBC contour map – ^{1}H x ^{13}C of 4'-chloroflavone (**D3**)	**37**
**Figure S47.** HMBC contour map – ^{1}H x ^{13}C expansion of 4'-chloroflavone (**D3**)	**37**
**Figure S48.** HMBC contour map – ^{1}H x ^{13}C expansion of 4'-chloroflavone (**D3**)	**38**
**Figure S49.** MS analysis of 6-chloroflavone (**D4**)	**38**
**Figure S50.** HPLC analysis of 6-chloroflavone (**D4**)	**38,39**
**Figure S51.** ^{1}H NMR spectrum (*δ,* acetone-d6, 600 MHz) of 6-chloroflavone (**D4**)	**39**
**Figure S52.** ^{1}H NMR spectrum expansion (*δ,* acetone-d6, 600 MHz) of 6-chloroflavone (**D4**)	**40**
**Figure S53.** ^{13}C NMR spectrum (*δ,* acetone-d6, 151 MHz) of 6-chloroflavone (**D4**)	**40**
**Figure S54.** ^{13}C NMR spectrum expansion (*δ,* acetone-d6, 151 MHz) of 6-chloroflavone (**D4**)	**41**
**Figure S55.** ^{13}C NMR spectrum expansion (*δ,* acetone-d6, 151 MHz) of 6-chloroflavone (**D4**)	**41**
**Figure S56.** COSY contour map – ^{1}H x ^{1}H of 6-chloroflavone (**D4**)	**42**
**Figure S57.** COSY contour map – ^{1}H x ^{1}H expansion of 6-chloroflavone (**D4**)	**42**
**Figure S58.** HMQC contour map – ^{1}H x ^{13}C of 6-chloroflavone (**D4**)	**43**
**Figure S59.** HMQC contour map – ^{1}H x ^{13}C expansion of 6-chloroflavone (**D4**)	**43**
**Figure S60.** HMBC contour map – ^{1}H x ^{13}C of 6-chloroflavone (**D4**)	**44**
**Figure S61.** HMBC contour map – ^{1}H x ^{13}C expansion of 6-chloroflavone (**D4**)	**44**
**Figure S62.** HMBC contour map – ^{1}H x ^{13}C expansion of 6-chloroflavone (**D4**)	**45**
**Figure S63.** MS analysis of 6,8-dichloroflavone (**D5**)	**45**
**Figure S64.** HPLC analysis of 6,8-dichloroflavone (**D5**)	**45,46**
**Figure S65.** ^{1}H NMR spectrum (*δ,* acetone-d6, 600 MHz) of 6,8-dichloroflavone (**D5**)	**47**
**Figure S66.** ^{1}H NMR spectrum expansion (*δ,* acetone-d6, 600 MHz) of 6,8-dichloroflavone (**D5**)	**47**
**Figure S67.** ^{13}C NMR spectrum (*δ,* acetone-d6, 151 MHz) of 6,8-dichloroflavone (**D5**)	**48**
**Figure S68.** ^{13}C NMR spectrum expansion (*δ,* acetone-d6, 151 MHz) of 6,8-dichloroflavone (**D5**)	**48**
**Figure S69.** ^{13}C NMR spectrum expansion (*δ,* acetone-d6, 151 MHz) of 6-dichloroflavone (**D5**)	**49**
**Figure S70.** COSY contour map – ^{1}H x ^{1}H of 6,8-dichloroflavone (**D5**)	**49**
**Figure S71.** COSY contour map – ^{1}H x ^{1}H expansion of 6,8-dichloroflavone (**D5**)	**50**
**Figure S72.** HMQC contour map – ^{1}H x ^{13}C of 6,8-dichloroflavone (**D5**)	**50**
**Figure S73.** HMQC contour map – ^{1}H x ^{13}C expansion of 6,8-dichloroflavone (**D5**)	**51**
**Figure S74.** HMBC contour map – ^{1}H x ^{13}C of 6,8-dichloroflavone (**D5**)	**51**
**Figure S75.** HMBC contour map – ^{1}H x ^{13}C expansion of 6,8-dichloroflavone (**D5**)	**52**
**Figure S76.** HMBC contour map – ^{1}H x ^{13}C expansion of 6,8-dichloroflavone (**D5**)	**52**

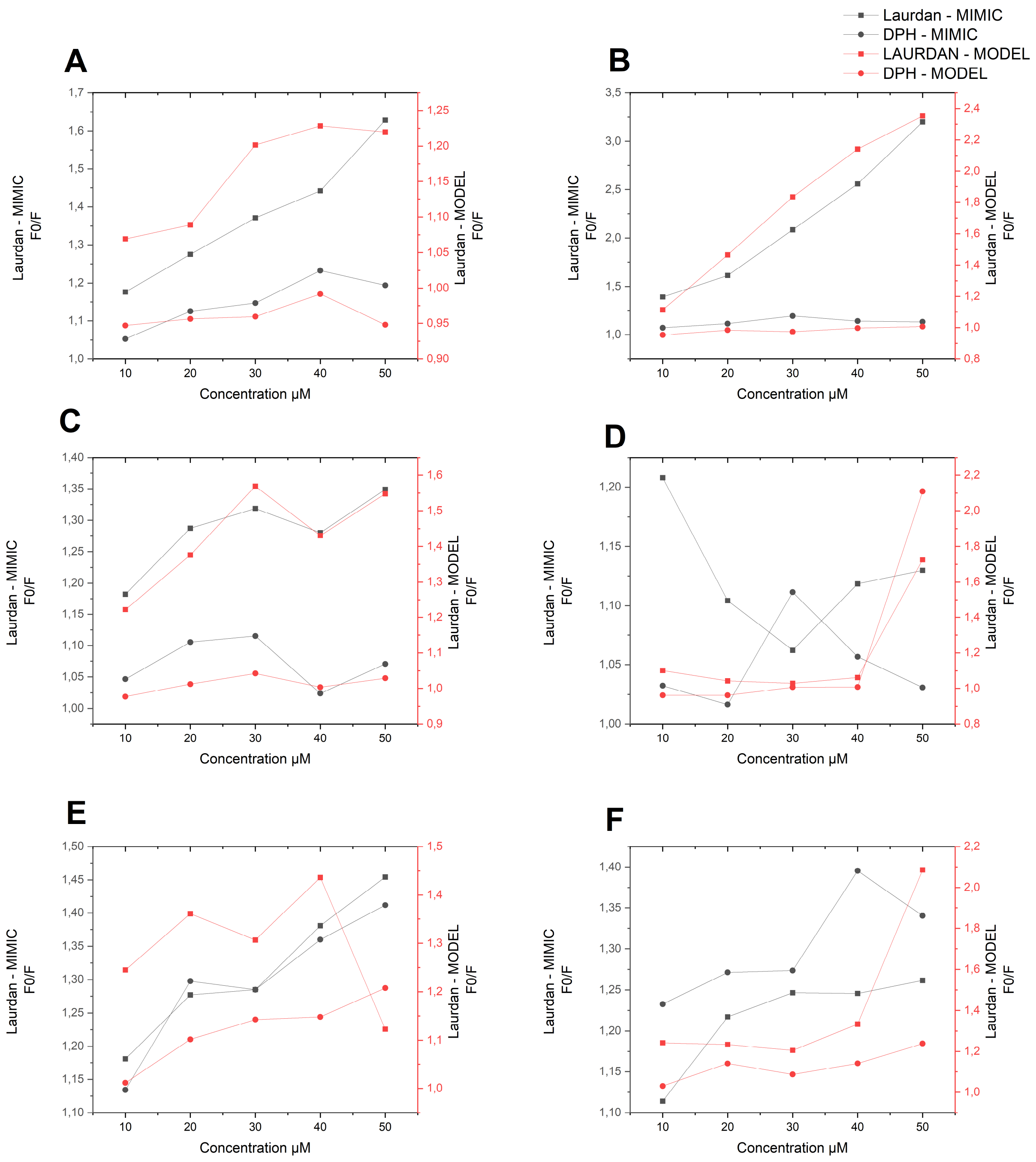

Figure S1. Stern–Volmer plots for D1 (A), D2 (B), D3 (C), D4 (D), D5 (E), D6 (F) induced quenching of Laurdan and DPH probes. F0 and F denote the absence and the presence of a quencher, respectively. The F0/F ratio was plotted against the quencher concentration.

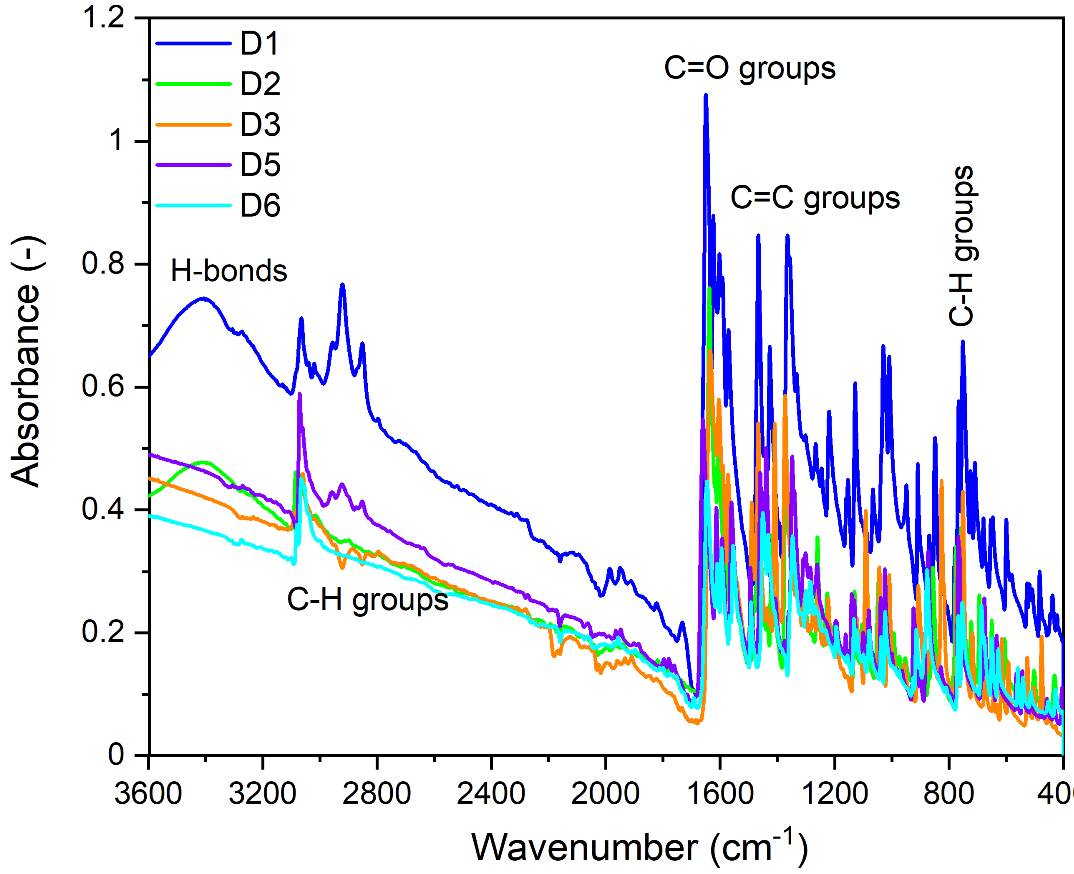

Figure S2. Raw ATR-FTIR powder spectra of D1,D2, D3, D5 and D6 compounds in the range of 3600-400 cm^{-1}.

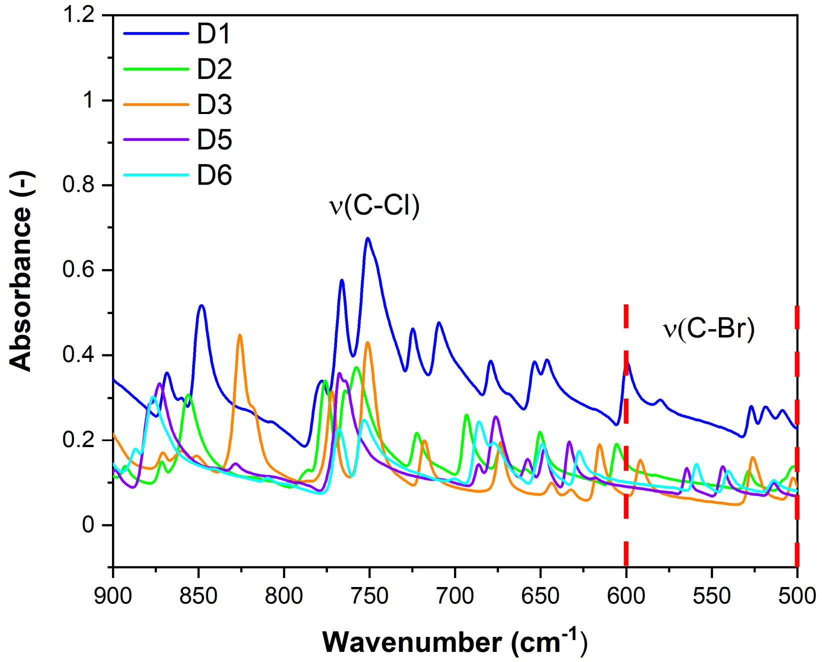

Figure S3. Raw ATR-FTIR powder spectra of D3, D5 and D6 compounds in the range of 900-500 cm^{-1}.

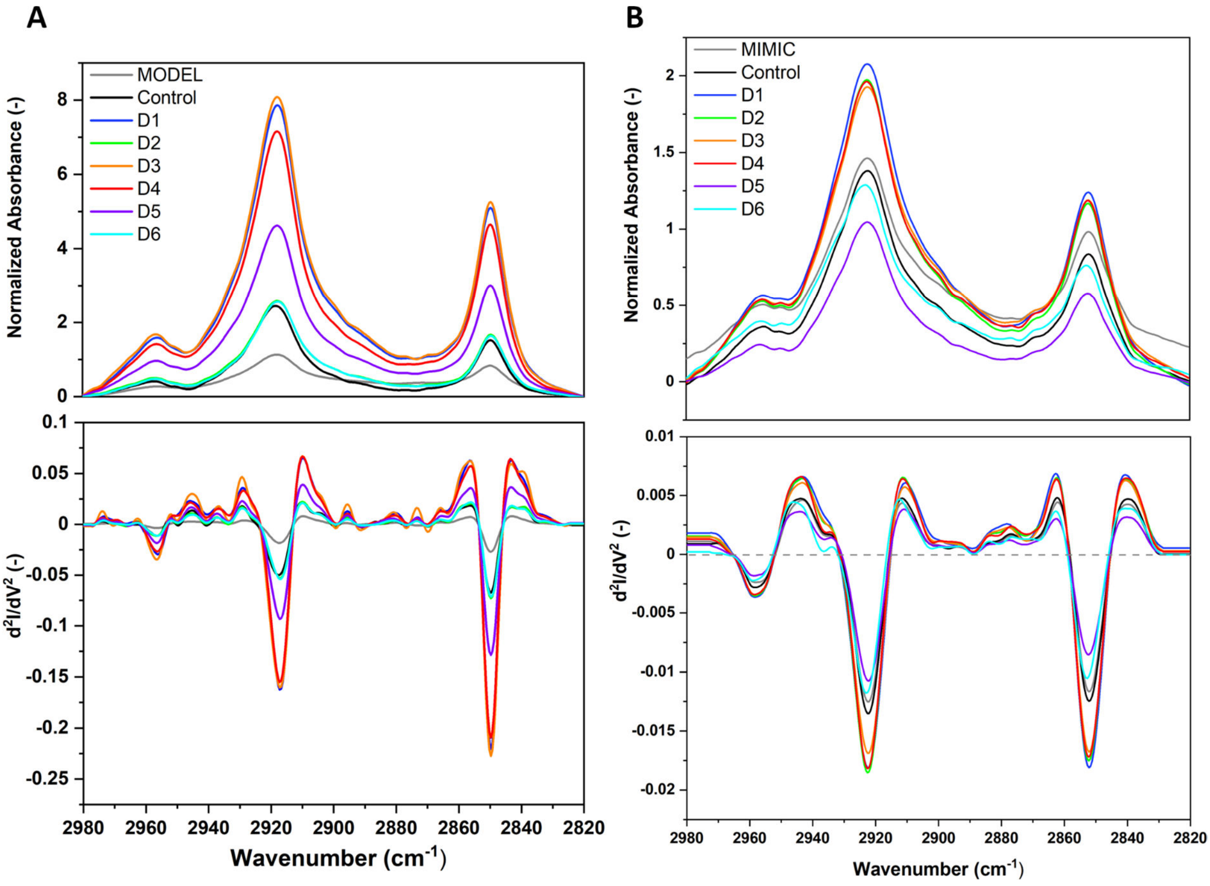

Figure S4. Normalized ATR-FTIR spectra of D1 to D6 compounds with MODEL (A) and MIMIC (B) lipid membranes, in the range of 2980-2820 cm^{-1} with second derivatives, directly after dissolving. Spectra normalized to the CH_2 group (1450 cm^{-1}).

Table S1. Tentative assignments of bands in ATR-FTIR spectra of the studied D1-D6 halogenated flavonoid derivatives with lipid membranes (MODEL and MIMIC) in dry-film form at 37°C, where '↓' denotes a shift to a lower wavenumber compared to the control, and '↑' indicates a shift to a higher wavenumber compared to the control.

Group	MODEL Control	D1	D2	D3	D4	D5	D6	Assignment
**ν_{as}(—C—H)**	**2956**	2956	2956	2956	2956	2956	2956	Asymmetric CH_3 stretching
**ν_{s}(—C—H)**	**2917**	2917	2917	2917	2917	2917	2917	Asymmetric CH_2 stretching
**ν_{s}(—C—H)**	**2870**	2870	2870	2870	2870	2870	2870	Symmetric CH_3 stretching
**ν_{as}(—C—H)**	**2850**	2850	2850	2850	2850	2850	2850	SymmetricCH_2 stretching
**$\nu_{as}(PO_2^-)$**	**1246**	1245↓	1243 ↓	1245 ↓	1252 ↑	1245 ↓	1245 ↓	Asymmetric PO_2^- stretching
**$\nu_{s}(PO_2^-)$**	**1056**	1091↑	1055 ↓	1091 ↑	1132 ↑	1091 ↑	1054 ↓	Symmetric PO_2^- stretching
**$\nu(C{=}O)_{bond}$**	**1744**	1740↓	1743 ↓	1743 ↓	1741 ↓	1744	1743 ↓	C=O stretching *(nonhydrated)*
**$\nu(C{=}O)_{free}$**	**1725**	1724↓	1735 ↑	1729 ↑	1725	1725	1725	C=O stretching *(hydrated)*
**ν(C=O)**	**1729**	1729	1729	1734 ↑	1729	1729	1729	Stretching C=O
**$\nu_{as}(N(CH_3)_3)$**	**951**	951	951	951	951	951	951	Asymmetric N-$(CH_3)_3$ stretching
**$\nu_{as}(N(CH_3)_3)$**	**975**	970↓	971↓	970↓	968↓	970↓	971↓	Asymmetric N-$(CH_3)_3$ stretching
**Group**	**MIMIC Control**	**D1**	**D2**	**D3**	**D4**	**D5**	**D6**	**Assignment**
**ν_{as}(—C—H)**	**2958**	2958	2958	2958	2958	2958	2958	Asymmetric CH_3 stretching
**ν_{s}(—C—H)**	**2923**	2923	2923	2923	2923	2923	2923	Asymmetric CH_2 stretching
**ν_{s}(—C—H)**	**2871**	2871	2871	2871	2871	2871	2871	Symmetric CH_3 stretching
**ν_{as}(—C—H)**	**2852**	2852	2852	2852	2852	2852	2852	Symmetric CH_2 stretching
**$\nu_{as}(PO_2^-)$**	**1241**	1245↑	1243 ↑	1258 ↑	1251 ↑	1245 ↑	1240 ↓	Asymmetric PO_2^- stretching

v $_{s}$**(PO_2^-)**	**1065**	1067↑	1068 ↑	1067 ↑	1065 ↑	1072 ↑	1068 ↑	Symmetric PO_2^- stretching
v**(C=O)**$_{\mathbf{bond}}$	**1741**	1740↓	1743 ↑	1743 ↑	1743 ↑	1740 ↓	1740 ↓	C=O stretching *(nonhydrated)*
v **(C=O)**$_{\mathbf{free}}$	**1725**	1725	-	-	1725	1725	1725	C=O stretching *(hydrated)*
v **(C=O)**	**1728**	1729↑	1728	1728	1728	1729 ↑	1729 ↑	Stretching C=O
v $_{\mathbf{as}}$**(N(CH_3)$_3$)**	**951**	951	951	951	951	951	951	Asymmetric N-(CH_3)$_3$ stretching
v $_{\mathbf{as}}$**(N(CH_3)$_3$)**	**983**	983	983	972↓	983	976↓	975↓	Asymmetric N-(CH_3)$_3$ stretching

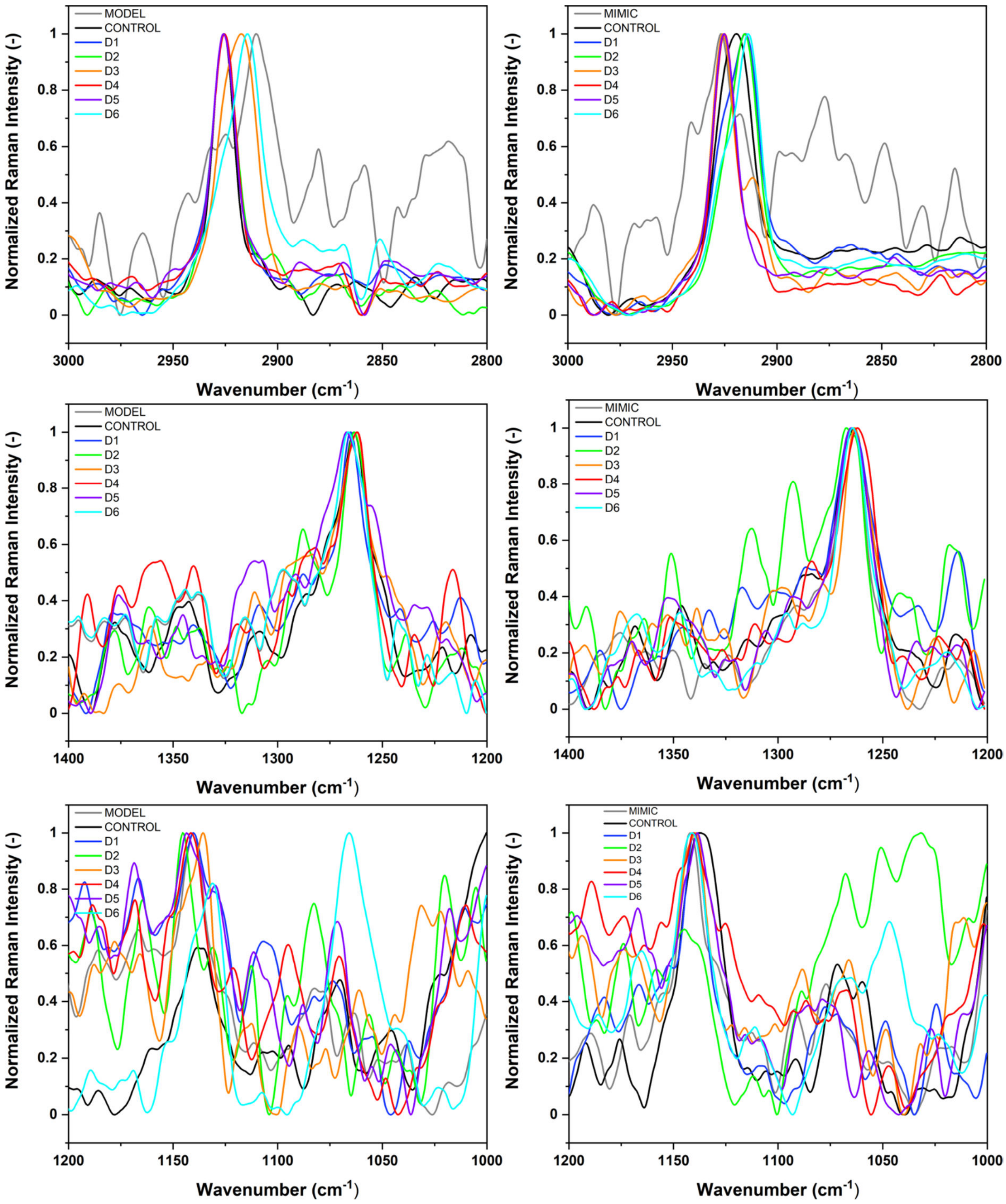

Figure S5. Normalized FT-Raman spectra of D1 to D6, MODEL, MIMIC membranes and their interactions in regions: (3000−2800 cm^{-1}), (1400−1200 cm^{-1}), and (1200−1000 cm^{-1}). Spectra normalized to [0 1] in the analyzed regions.

Table S2. Tentative assignments of the local maxima presented in the FT-Raman spectra of the studied D1-D6 halogenated flavonoid derivatives with lipid membranes (MODEL and MIMIC) in dry-film form at room temperature, where 'sh' denotes the shoulder band. Additionally, rows correspond to the most intensive bands, which are filled in lilac color.

Group	MODEL Control	D1	D2	D3	D4	D5	D6	Assignment
ν_{as}(—C—H)	**2955**	2955	2952	2947	2953	2950	2948	Asymmetric CH_3 stretching
ν_{s}(—C—H)	**2894**	2894	2902	2888	2901	2890	2888	Asymmetric CH_2 stretching
ν_{s}(—C—H)	**2925**	2925	2925	2917	2925	2926	2915	Symmetric CH_3 stretching
ν_{as}(—C—H)	**2850**	2850	2850	2851	2850	2850	2851	Symmetric CH_2 stretching
δ(=CH)	**1263**	1265	1263	1262	1262	1267	1266	Deformation CH
ν(—C—C)	**1137**	1140	1145	1336	1141	1144	1131	C-C stretching
**Group**	**MIMIC Control**	**D1**	**D2**	**D3**	**D4**	**D5**	**D6**	**Assignment**
ν_{as}(—C—H)	**2969**	2945	2947	2941	2944	2949	2947	Asymmetric CH_3 stretching
ν_{s}(—C—H)	**2889**	2894	2882	2894	2891	2892	2893	Asymmetric CH_2 stretching
ν_{s}(—C—H)	**2919**	2915	2916	2926,2911sh	2925	2925	2914	Symmetric CH_3 stretching
ν_{as}(—C—H)	**2843**	2843	2851	2850	2853	2844	2854	Symmetric CH_2 stretching
δ(=CH)	**1264**	1265	1267	1263	1262	1265	1264	Deformation CH
ν(—C—C)	**1137**	1140	1145	1339	1140	1139	1141	C-C stretching

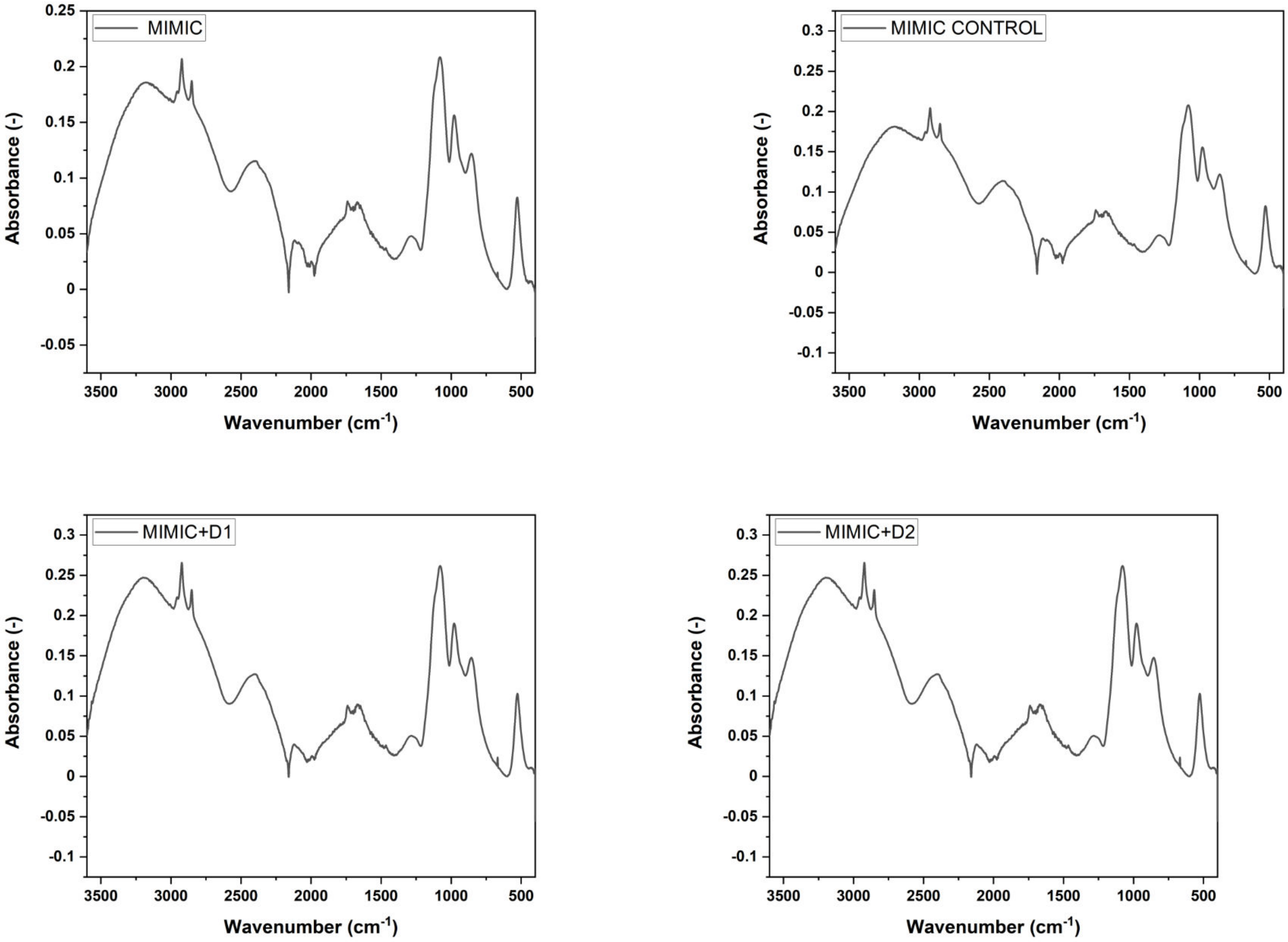
MIMIC
Absorbance (-)
Wavenumber (cm-1)
MIMIC CONTROL
Absorbance (-)
Wavenumber (cm-1)
MIMIC+D1
Absorbance (-)
Wavenumber (cm-1)
MIMIC+D2
Absorbance (-)
Wavenumber (cm-1)

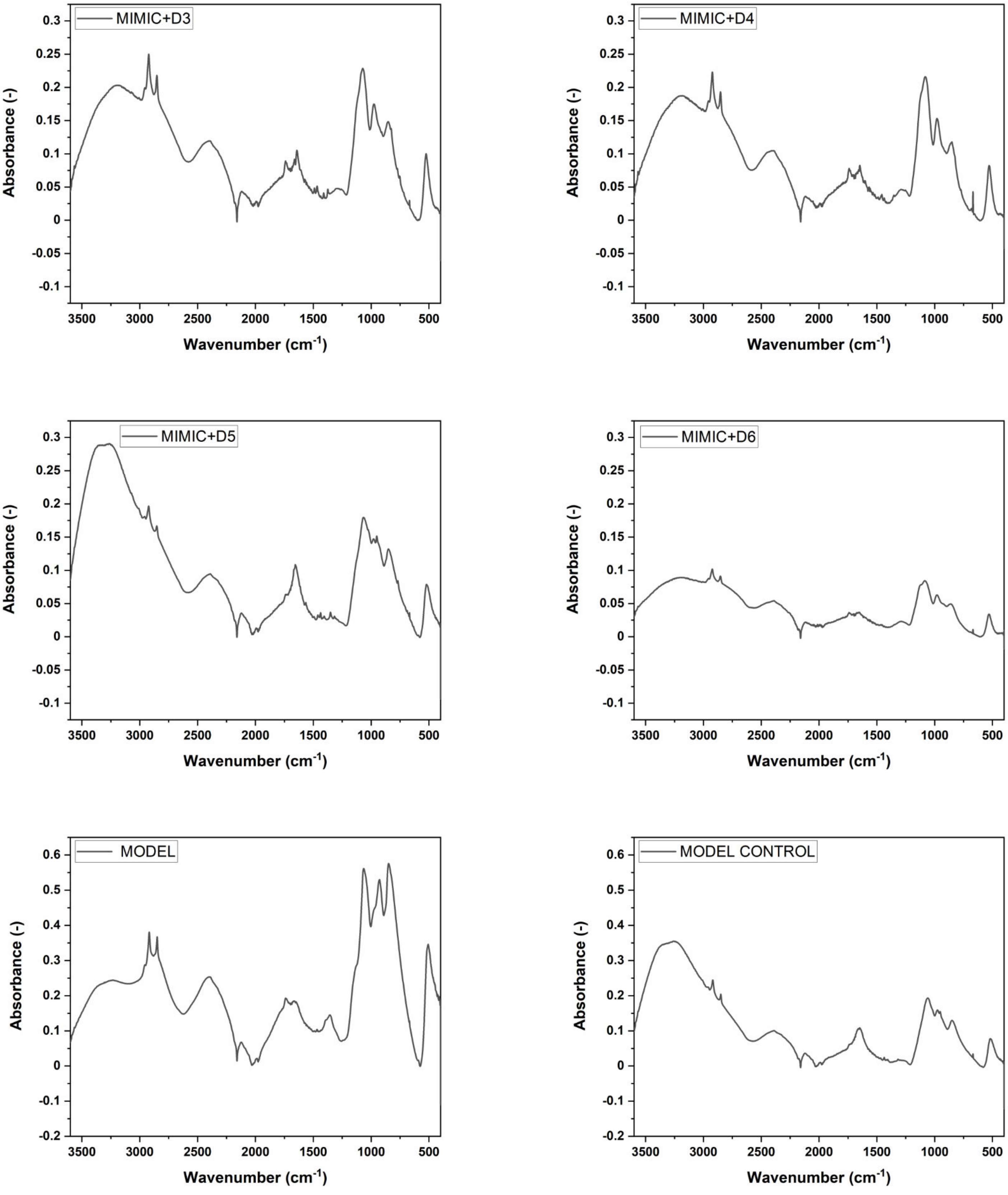
MIMIC+D3
MIMIC+D4
MIMIC+D5
MIMIC+D6
MODEL
MODEL CONTROL
Absorbance (-)
Wavenumber (cm-1)

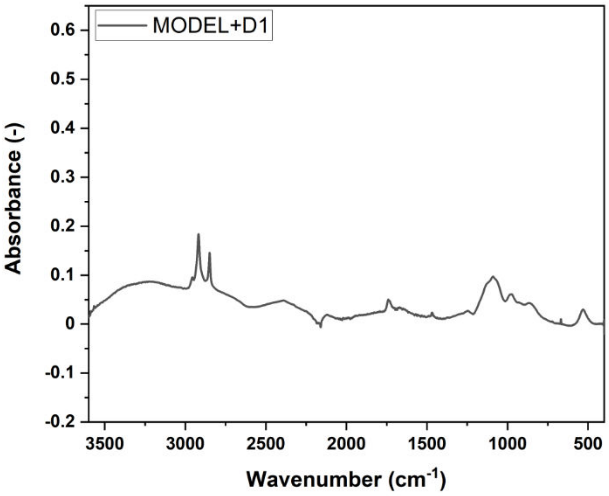
MODEL+D1
Absorbance (-)
Wavenumber (cm^{-1})

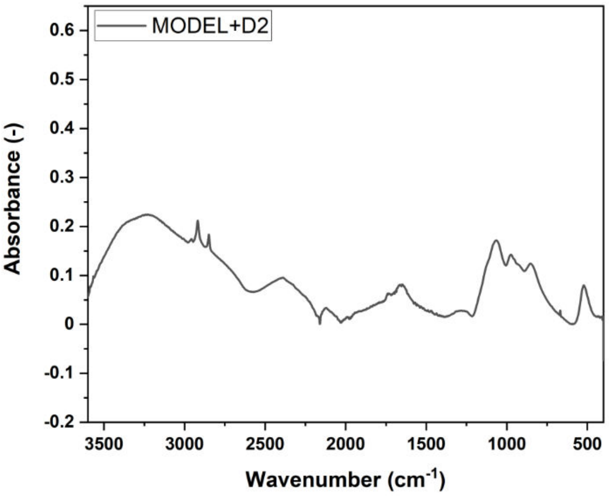
MODEL+D2
Absorbance (-)
Wavenumber (cm^{-1})

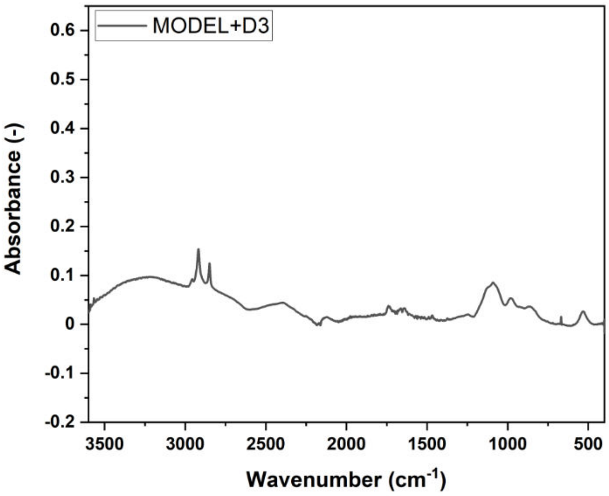
MODEL+D3
Absorbance (-)
Wavenumber (cm^{-1})

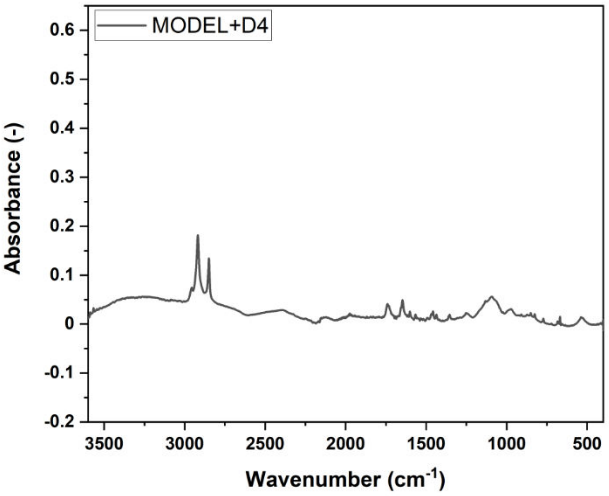
MODEL+D4
Absorbance (-)
Wavenumber (cm^{-1})

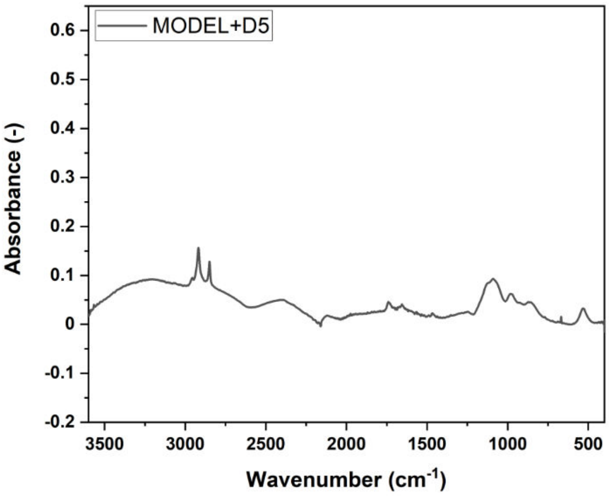
MODEL+D5
Absorbance (-)
Wavenumber (cm^{-1})

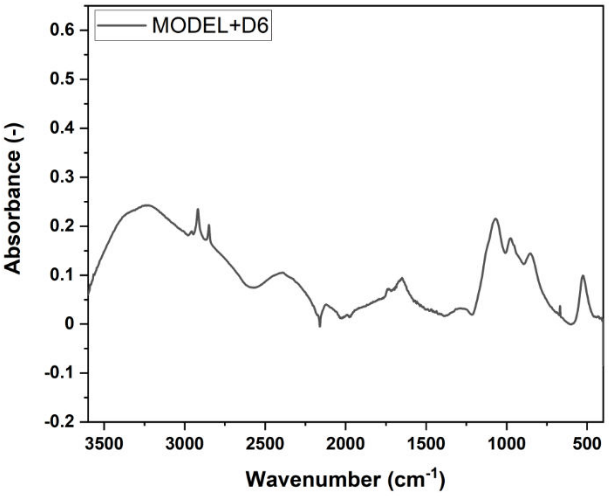
MODEL+D6
Absorbance (-)
Wavenumber (cm^{-1})

Figure S6. Raw ATR-FTIR spectra of D1 to D6, MODEL, MIMIC membranes and their interactions in the range of 3600-400 cm^{-1}.

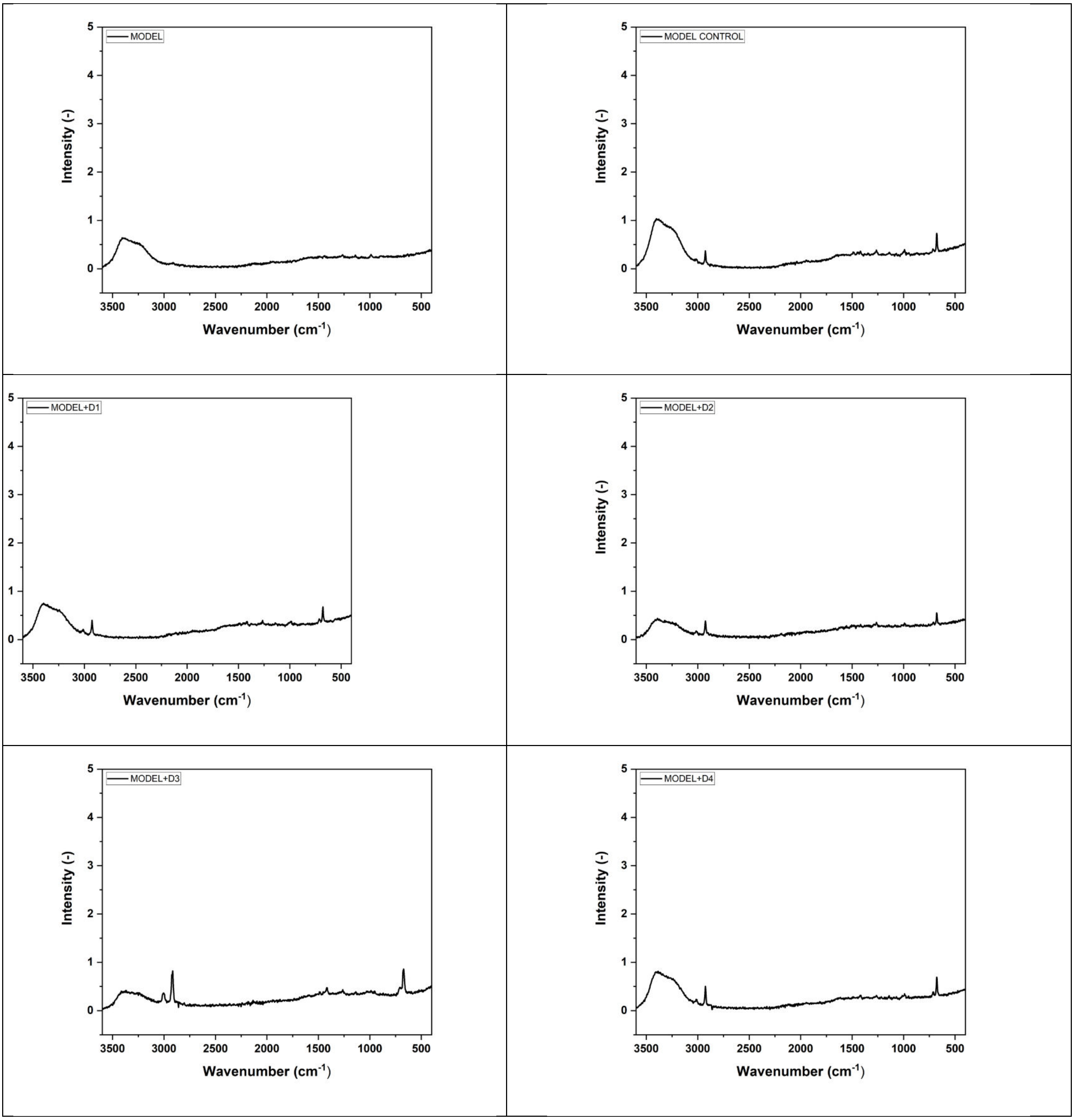

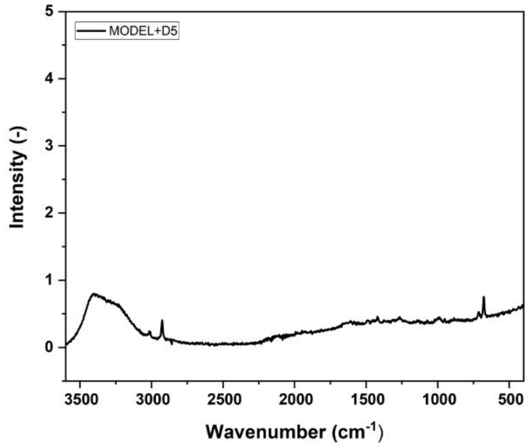
MODEL+D5
Intensity (-)
Wavenumber (cm-1)
0
1
2
3
4
5
3500
3000
2500
2000
1500
1000
500

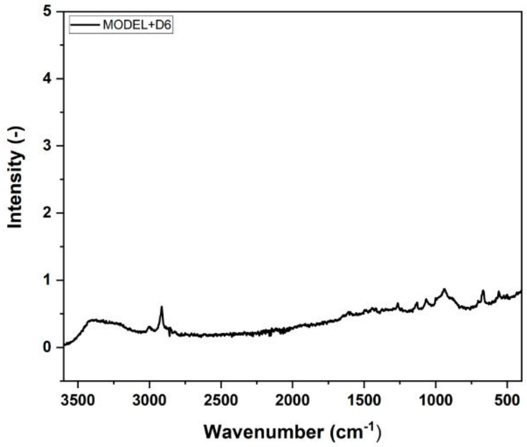
MODEL+D6
Intensity (-)
Wavenumber (cm-1)
0
1
2
3
4
5
3500
3000
2500
2000
1500
1000
500

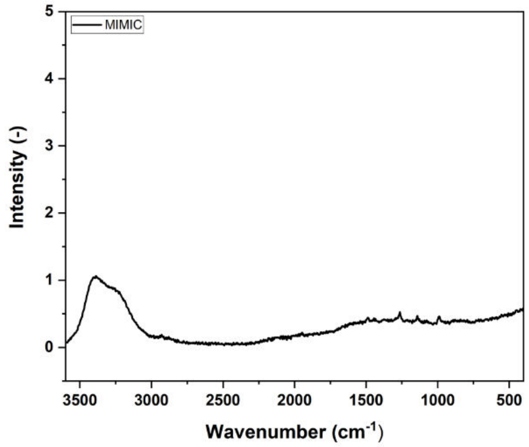
MIMIC
Intensity (-)
Wavenumber (cm-1)
0
1
2
3
4
5
3500
3000
2500
2000
1500
1000
500

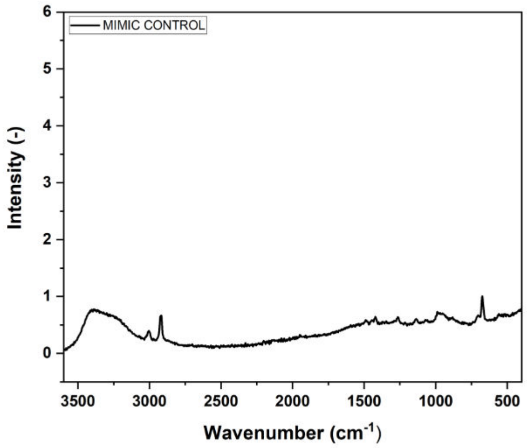
MIMIC CONTROL
Intensity (-)
Wavenumber (cm-1)
0
1
2
3
4
5
6
3500
3000
2500
2000
1500
1000
500

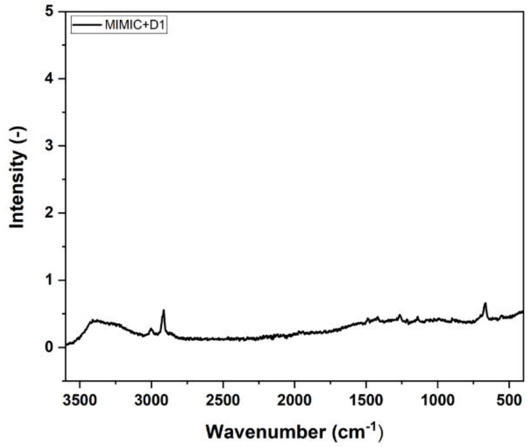
MIMIC+D1
Intensity (-)
Wavenumber (cm-1)
0
1
2
3
4
5
3500
3000
2500
2000
1500
1000
500

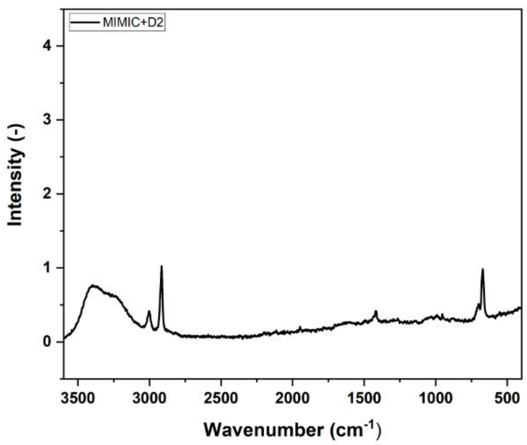
MIMIC+D2
Intensity (-)
Wavenumber (cm-1)
0
1
2
3
4
3500
3000
2500
2000
1500
1000
500

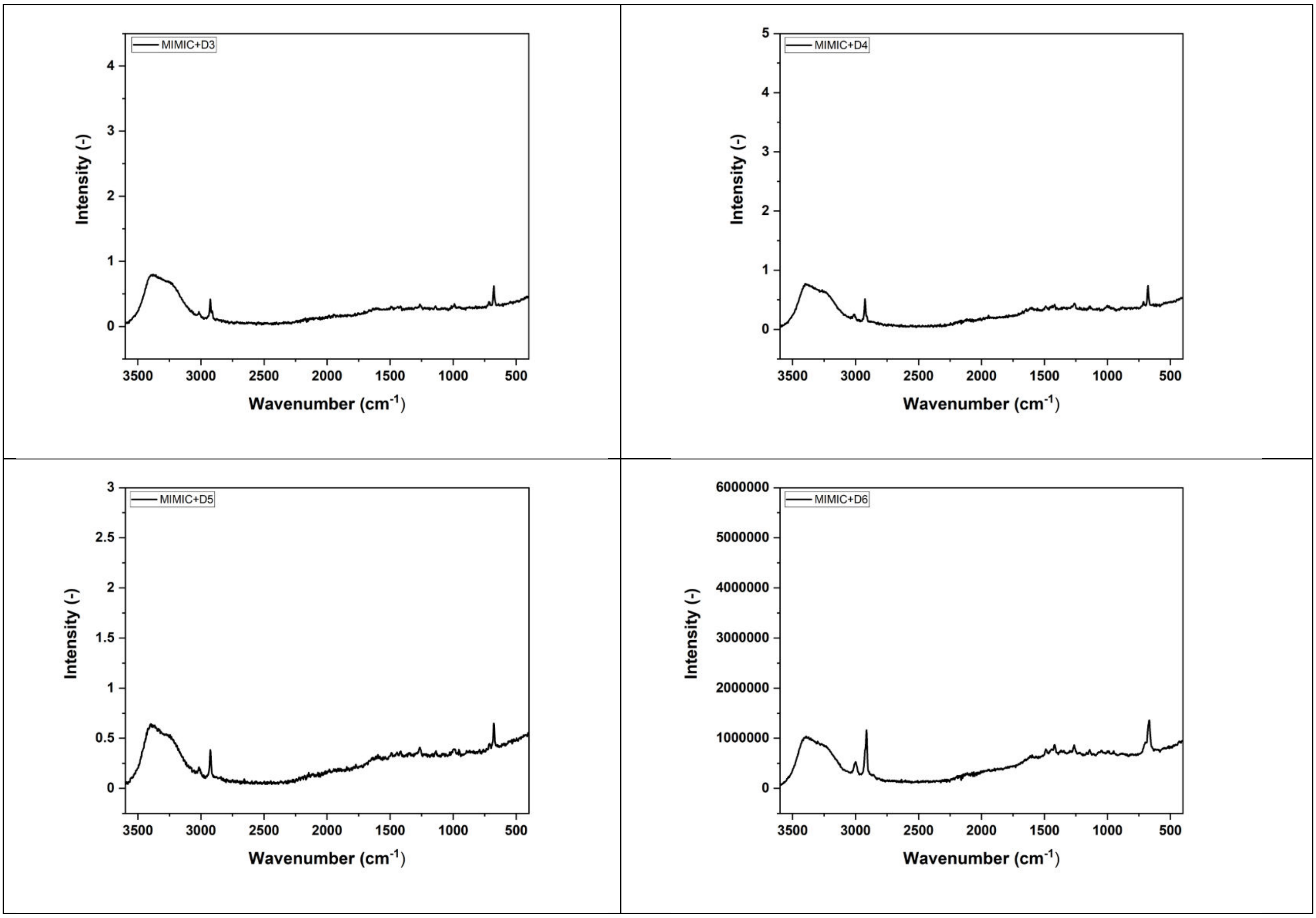

Figure S7. Raw FT-Raman spectra of D1 to D6, MODEL, MIMIC membranes and their interactions in the range of 3600-400 cm^{-1}.

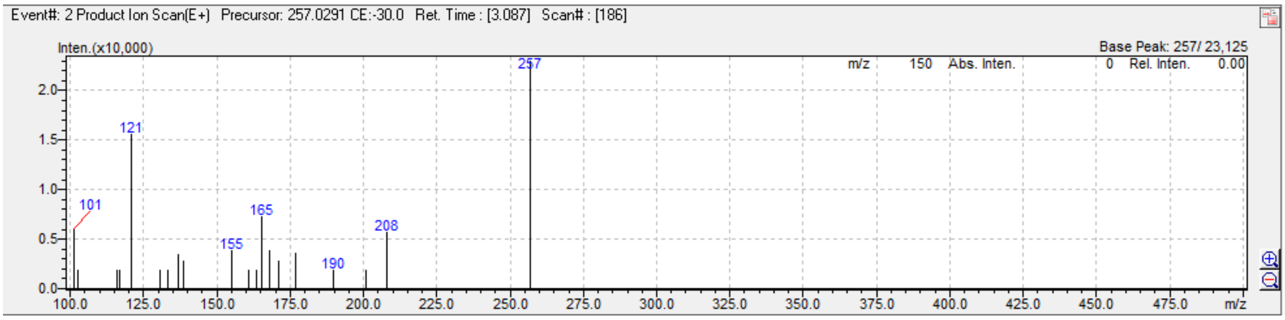

Figure S8. MS analysis of 2'-chloroflavone (**D1**)

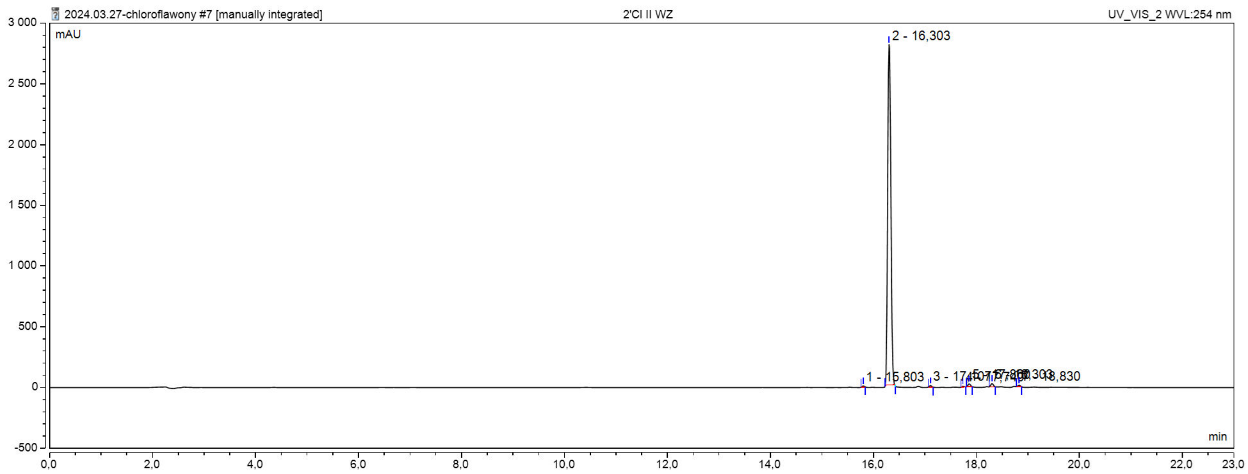

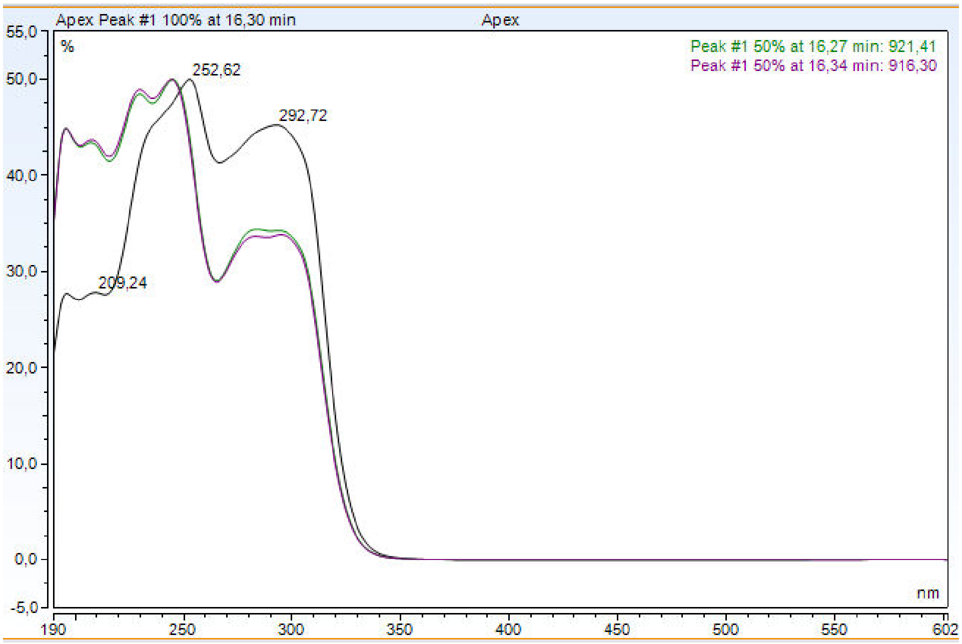

Peak	Peak Name	Ret.Time	Amount	Rel.Area	Area	Height	Type	Width (50%)	Asym.	Resol.	Plates
No.		min	n.a.	%	mAU*min	mAU		min	EP	EP	EP
UV_VIS_2	UV_VIS_2	UV_VIS_2	UV_VIS_2	UV_VIS_2	UV_VIS_2	UV_VIS_2	UV_VIS_2	UV_VIS_2	UV_VIS_2	UV_VIS_2	UV_VIS_2
1		15,803	n.a.	0,24	0,4908	10,45	BMB*	0,047	0,97	5,09	615436
2		16,303	n.a.	97,63	202,0784	2803,29	M *	0,069	1,16	8,05	313460
3		17,107	n.a.	0,30	0,6277	12,76	BMB*	0,049	1,11	8,19	669378
4		17,740	n.a.	0,17	0,3514	8,24	BMB*	0,042	1,03	1,52	987517
5		17,860	n.a.	0,62	1,2746	24,14	BMB*	0,051	1,06	4,92	669044
6		18,303	n.a.	0,75	1,5591	27,77	BMB*	0,055	1,05	5,79	615372
7		18,830	n.a.	0,29	0,6089	11,74	BMB*	0,052	0,95	n.a.	717238
Maximum			0,0000	97,63	202,0784	2803,29		0,069	1,16	8,19	987517
Minimum			0,0000	0,17	0,3514	8,24		0,042	0,95	1,52	313460
Sum			0,0000	100,00	206,9909	2898,40					

Figure S9. HPLC analysis of 2'-chloroflavone (D1)

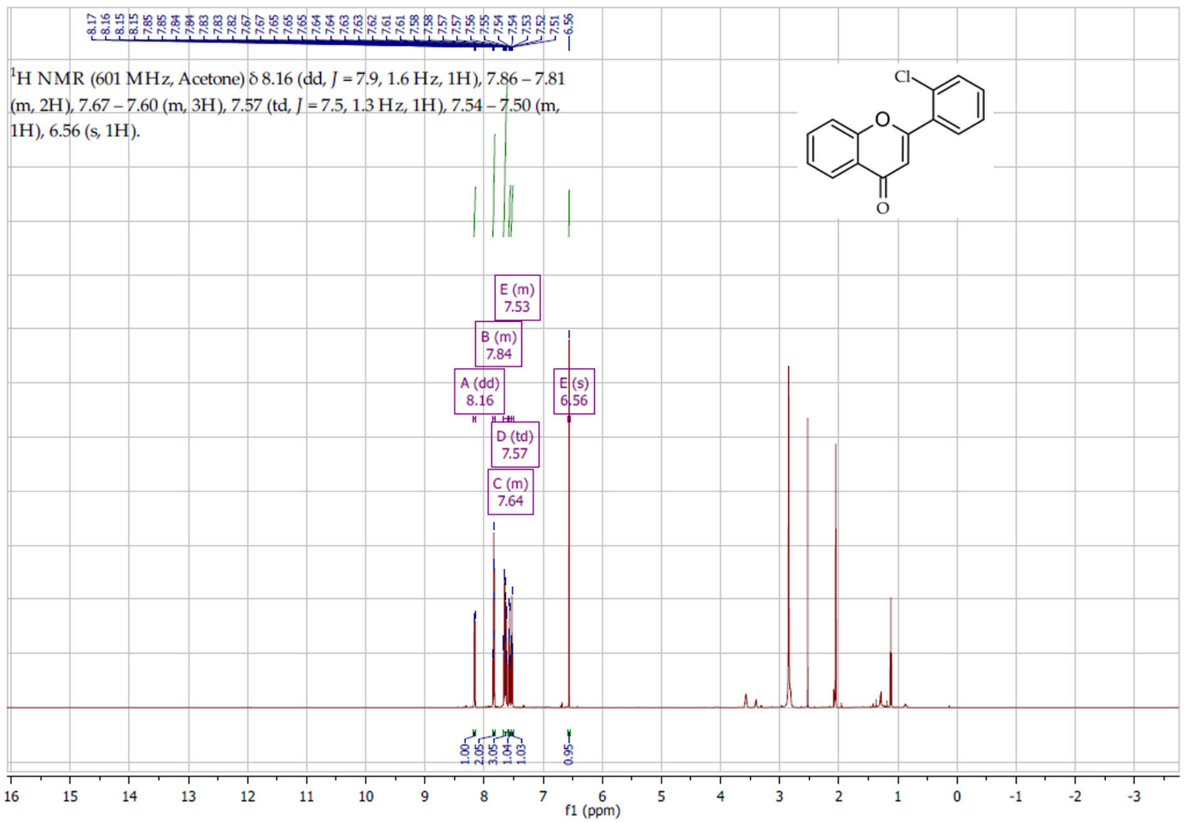

Figure S10. ^{1}H NMR spectrum (*δ,* acetone-d6, 600 MHz) of 2’-chloroflavone (**D1**)

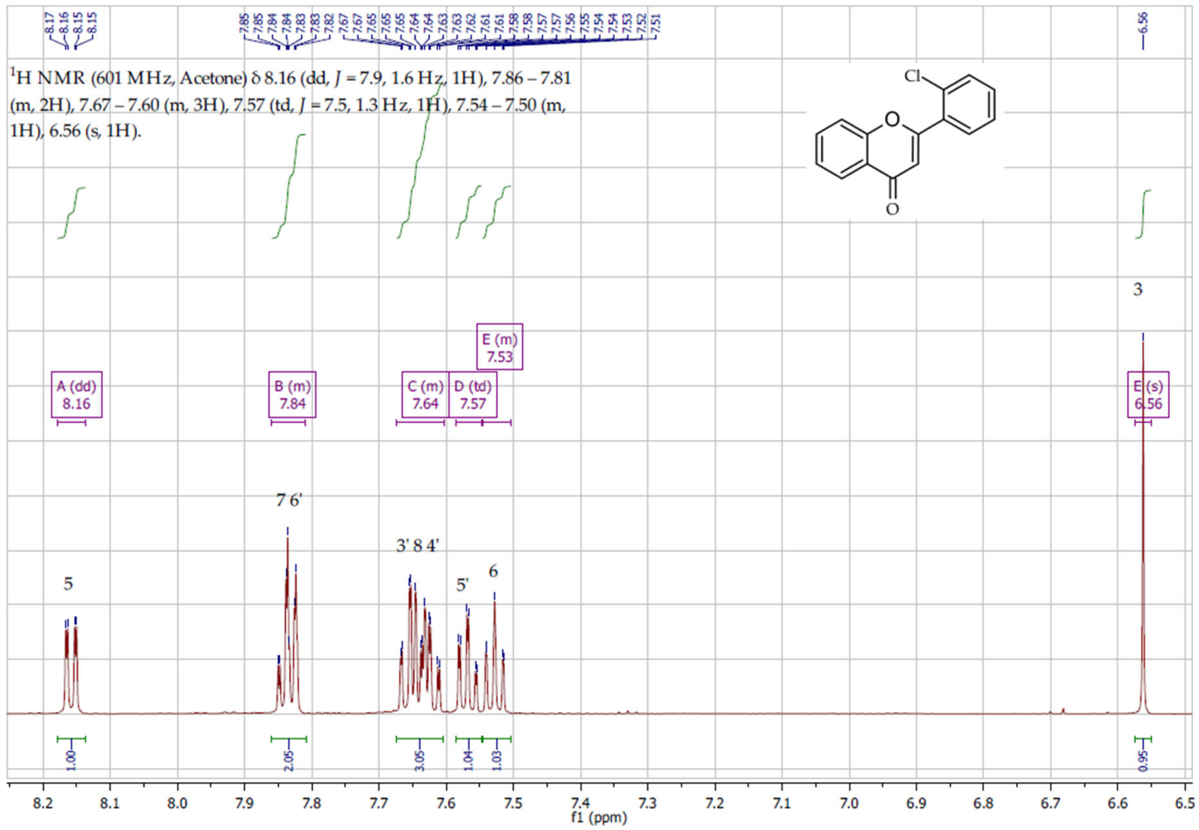

Figure S11. ^{1}H NMR spectrum expansion (*δ,* acetone-d6, 600 MHz) of 2’-chloroflavone (**D1**)

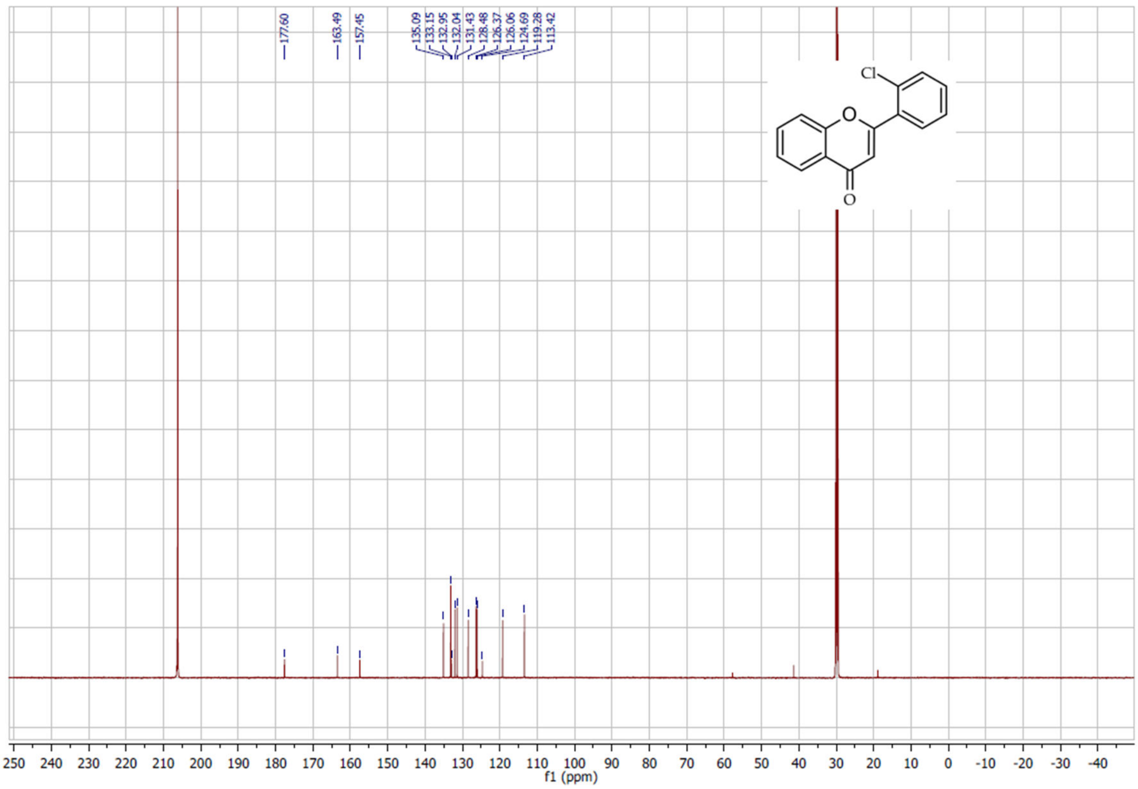

Figure S12. ^{13}C NMR spectrum (δ, acetone-d6, 151 MHz) of 2'-chloroflavone (**D1**)

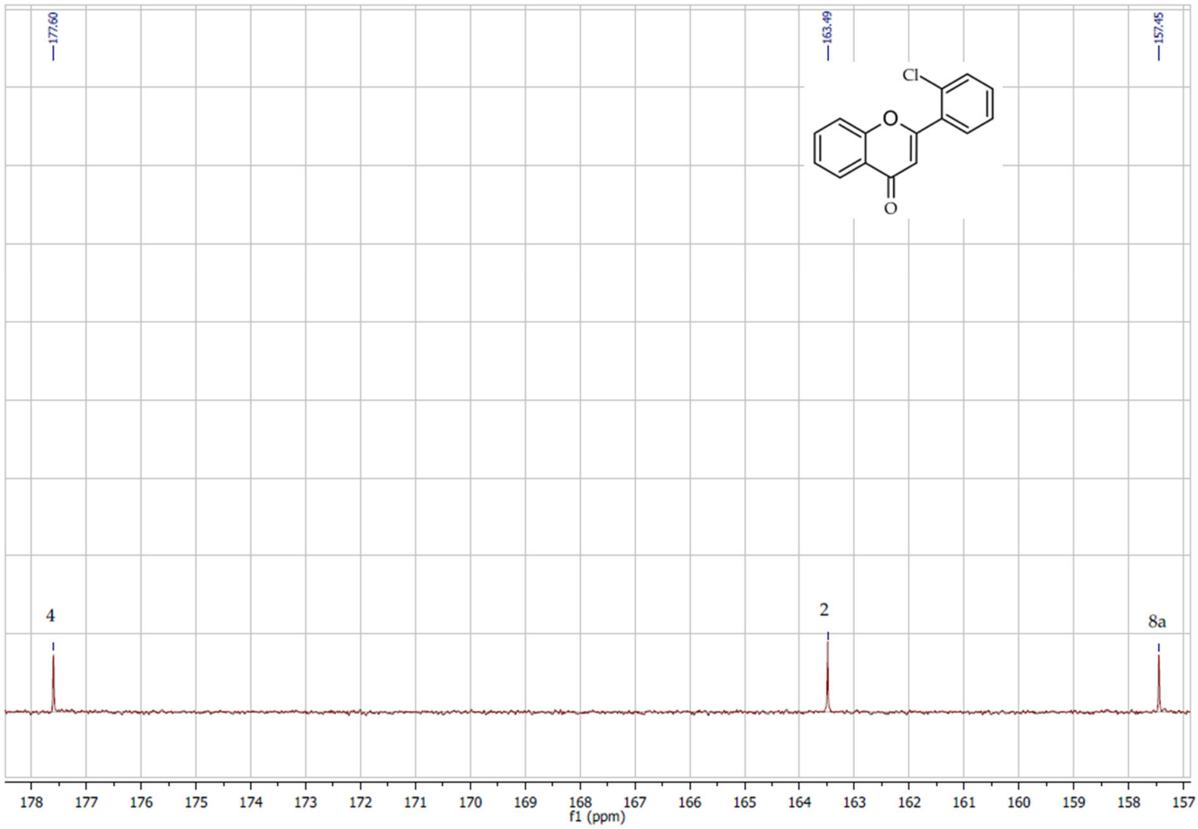

Figure S13. ^{13}C NMR spectrum expansion (δ, acetone-d6, 151 MHz) of 2'-chloroflavone (**D1**)

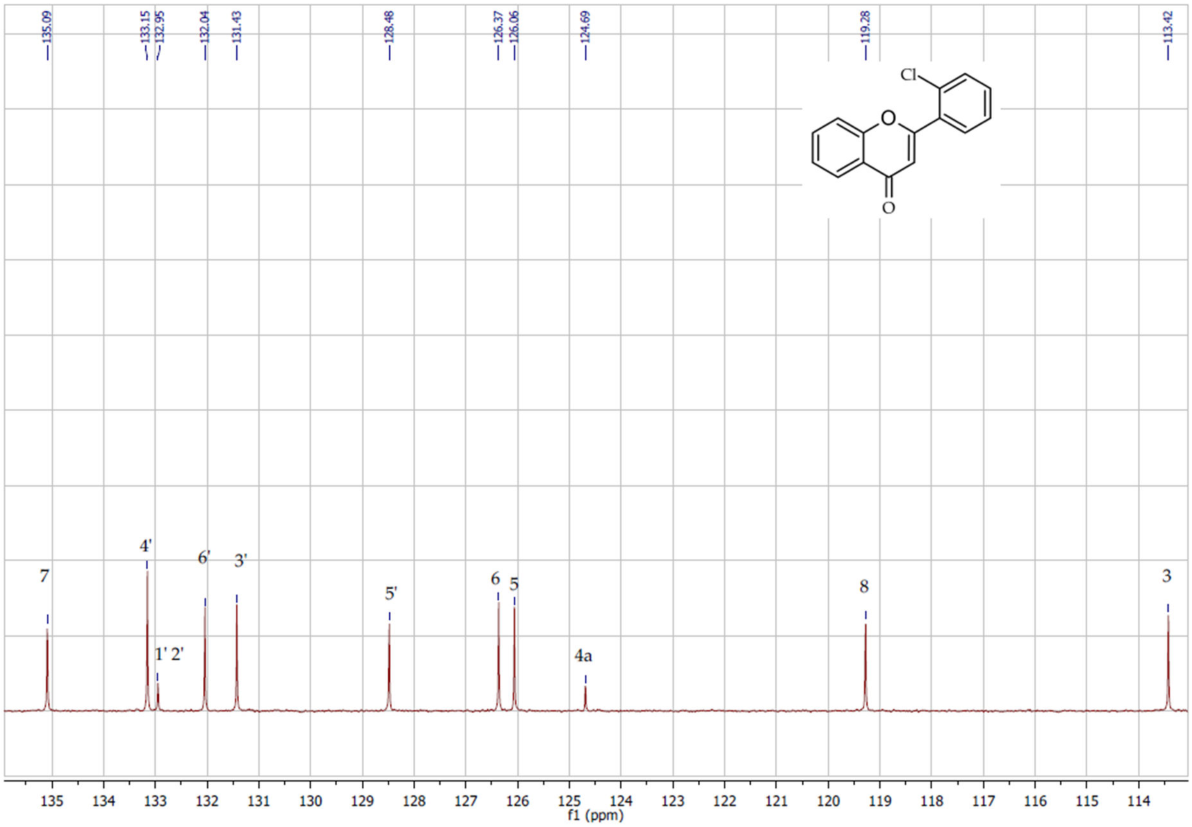

Figure S14. ^{13}C NMR spectrum expansion (δ, acetone-d6, 151 MHz) of 2'-chloroflavone (**D1**)

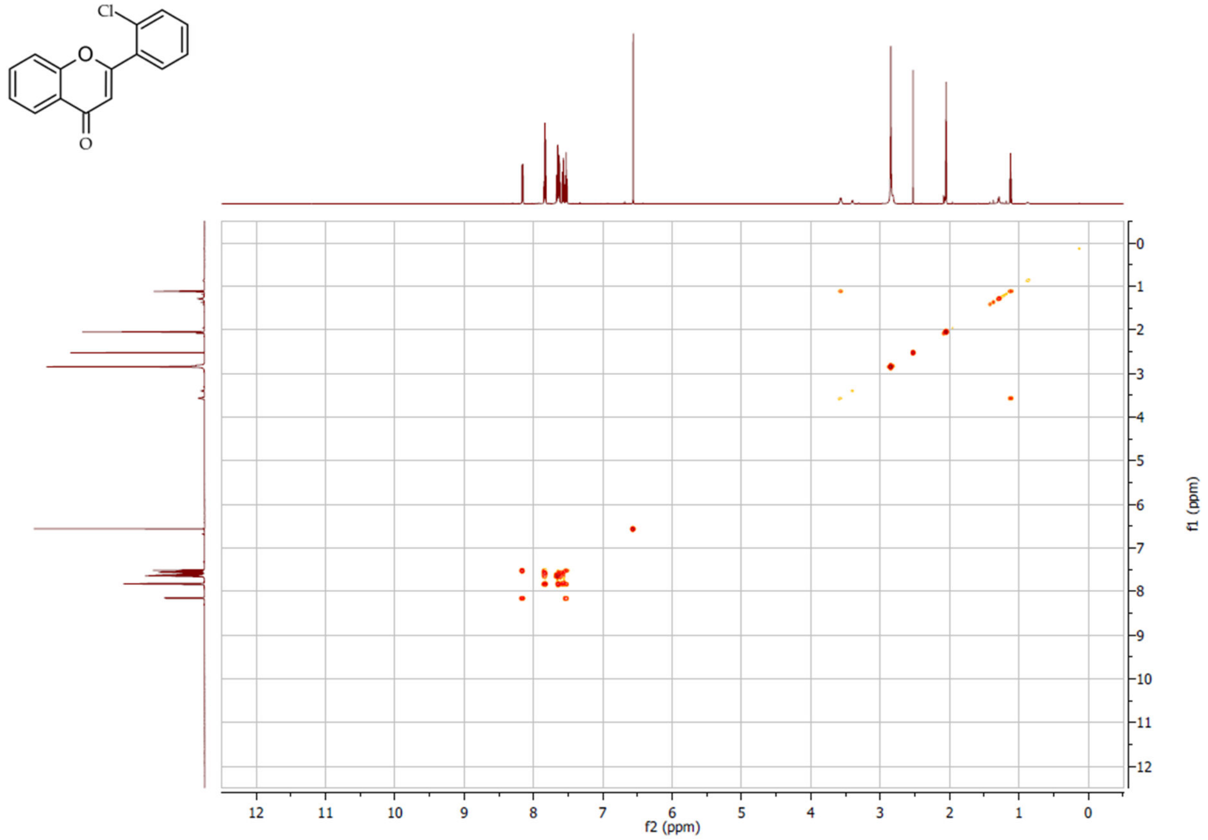

Figure S15. COSY contour map – ^1H x ^1H of 2'-chloroflavone (**D1**)

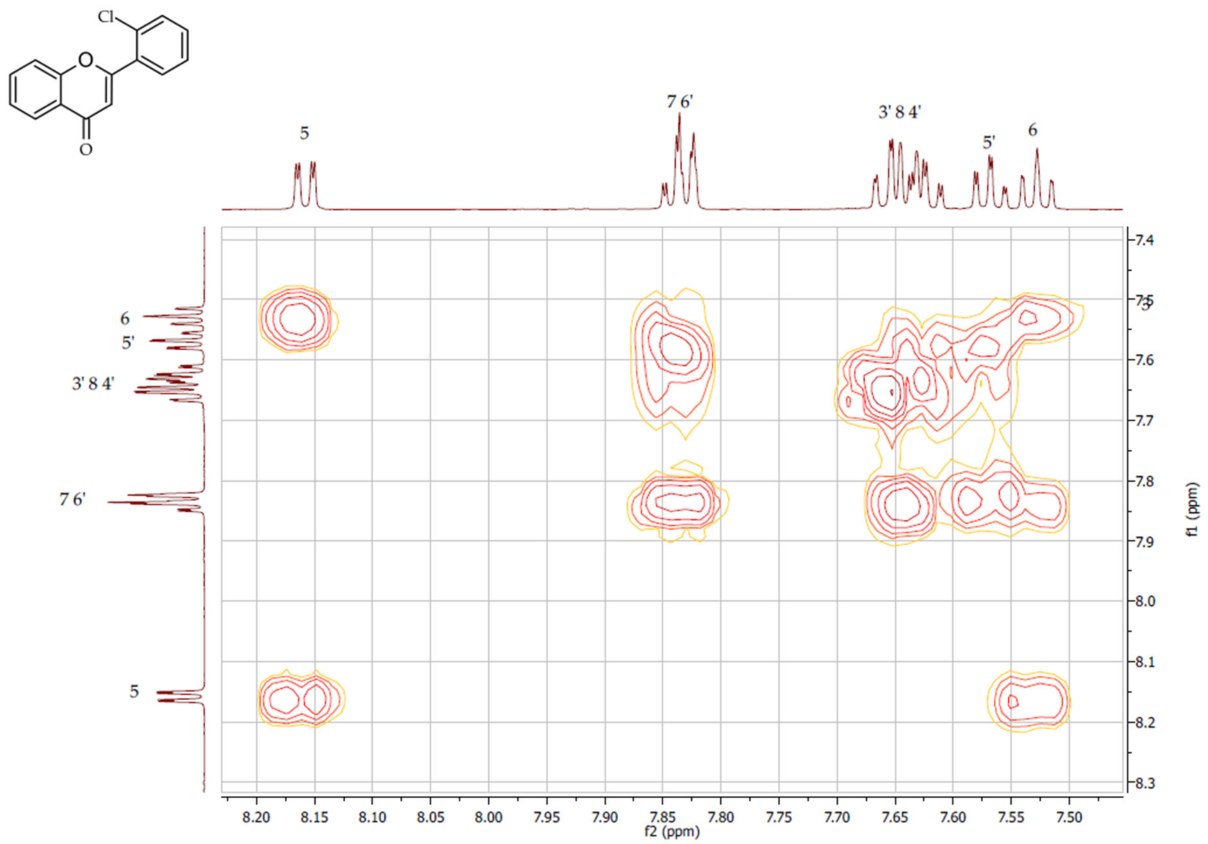

Figure S16. COSY contour map – ^{1}H x ^{1}H expansion of 2’-chloroflavone (**D1**)

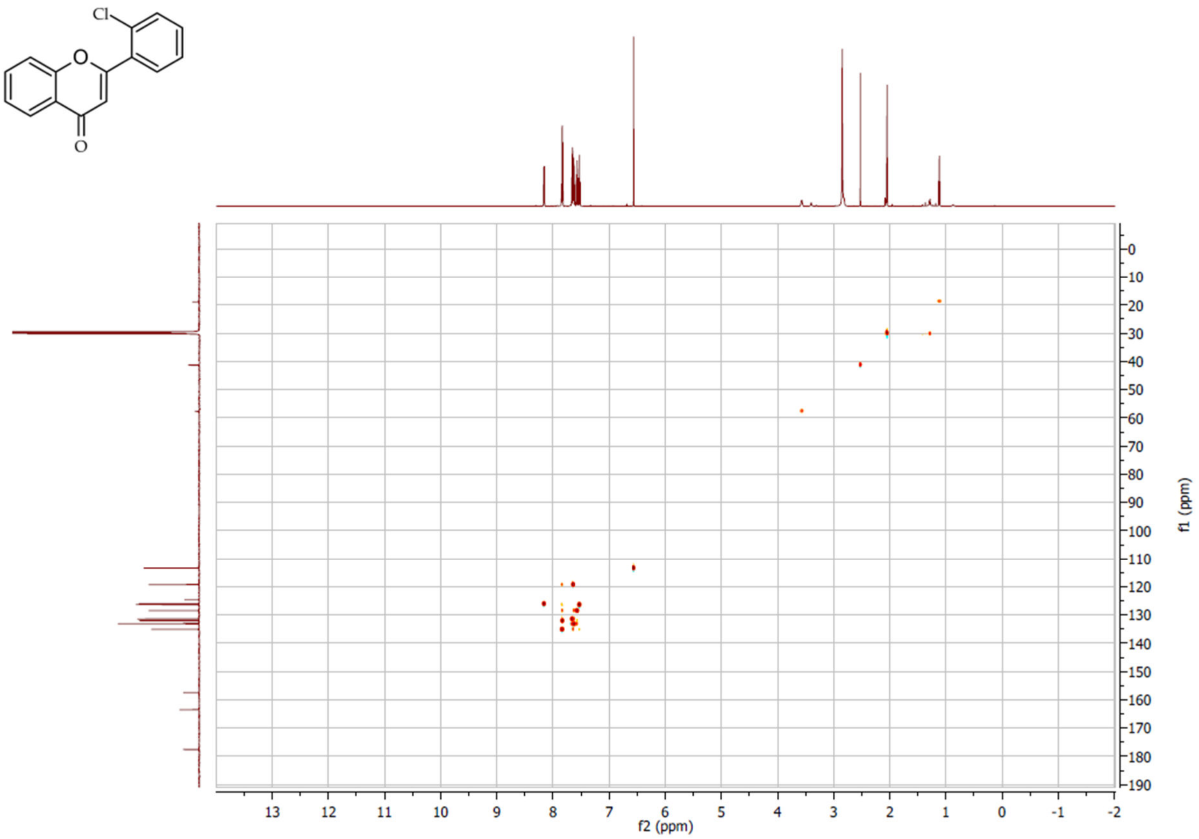

Figure S17. HMQC contour map – ^{1}H x ^{13}C of 2’-chloroflavone (**D1**)

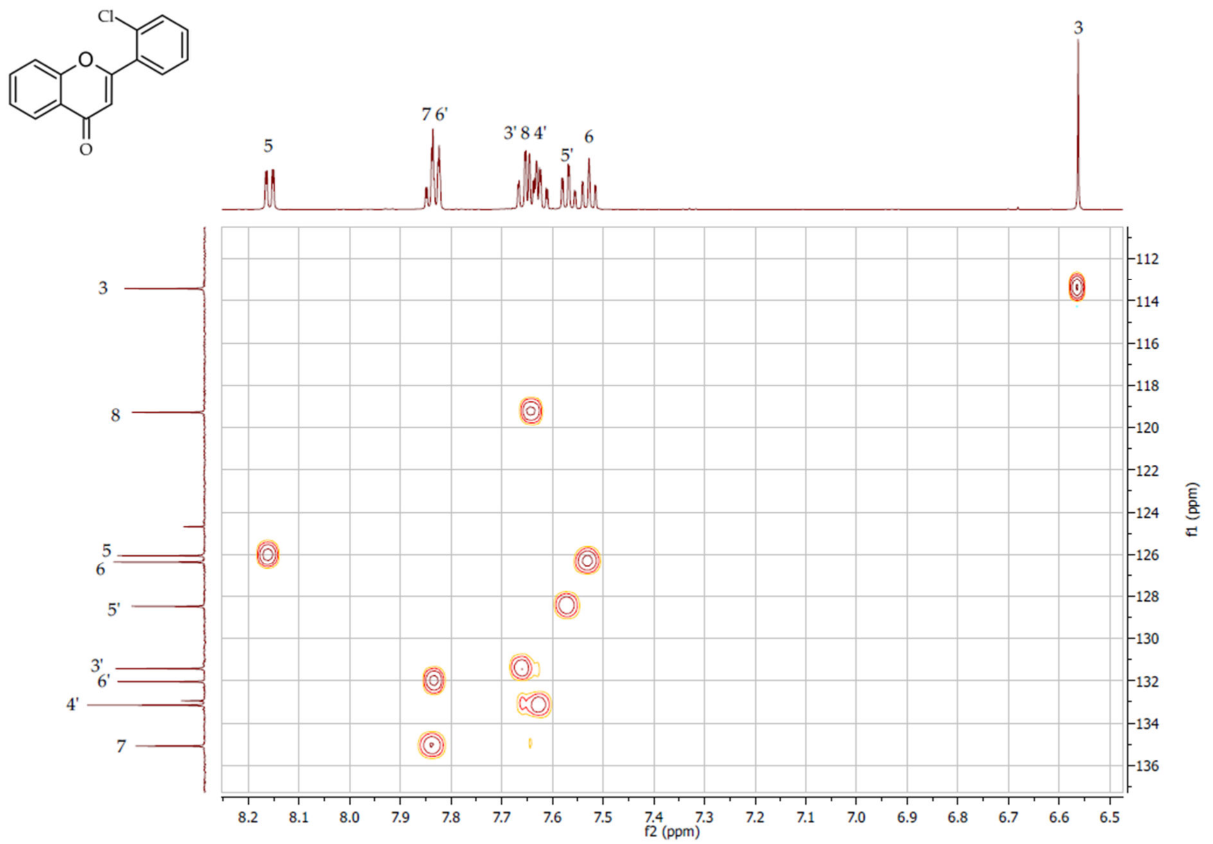

Figure S18. HMQC contour map – ^{1}H x ^{13}C expansion of 2'-chloroflavone (**D1**)

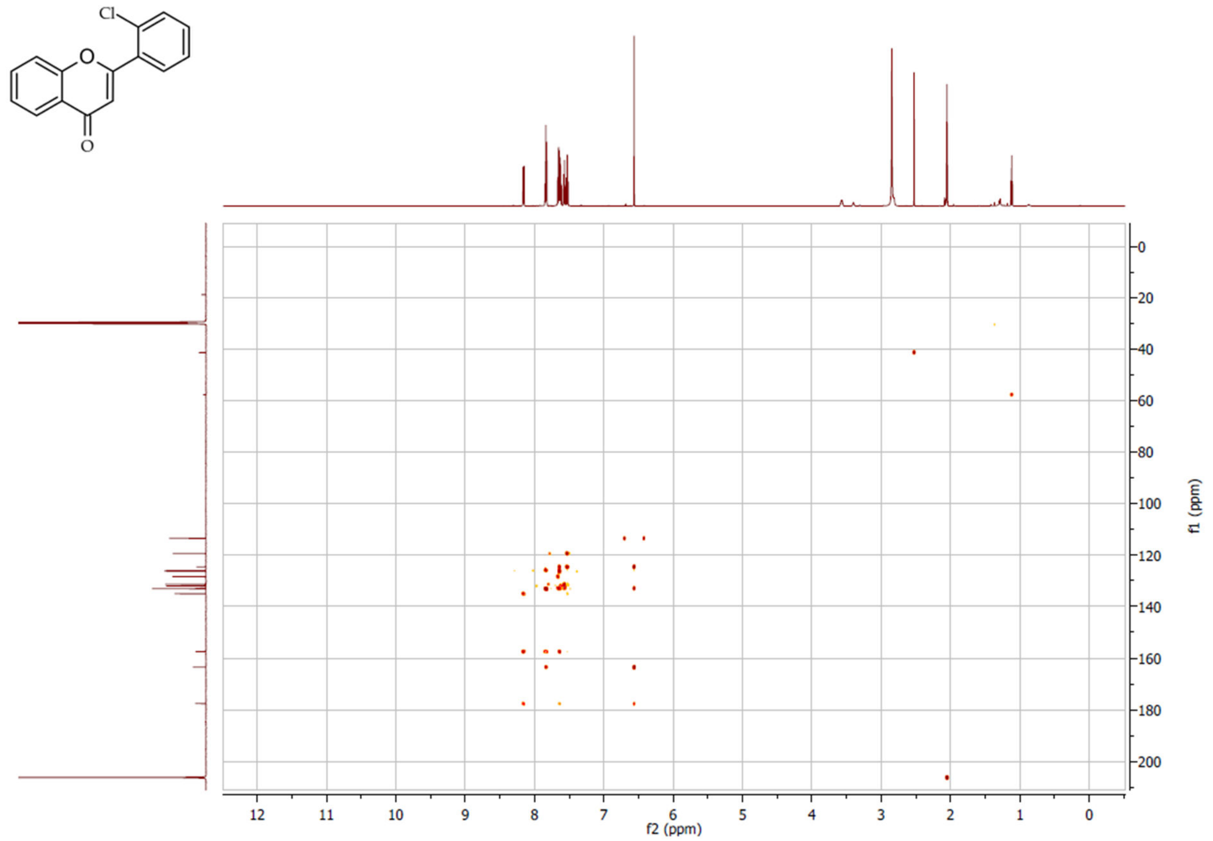

Figure S19. HMBC contour map – ^{1}H x ^{13}C of 2'-chloroflavone (**D1**)

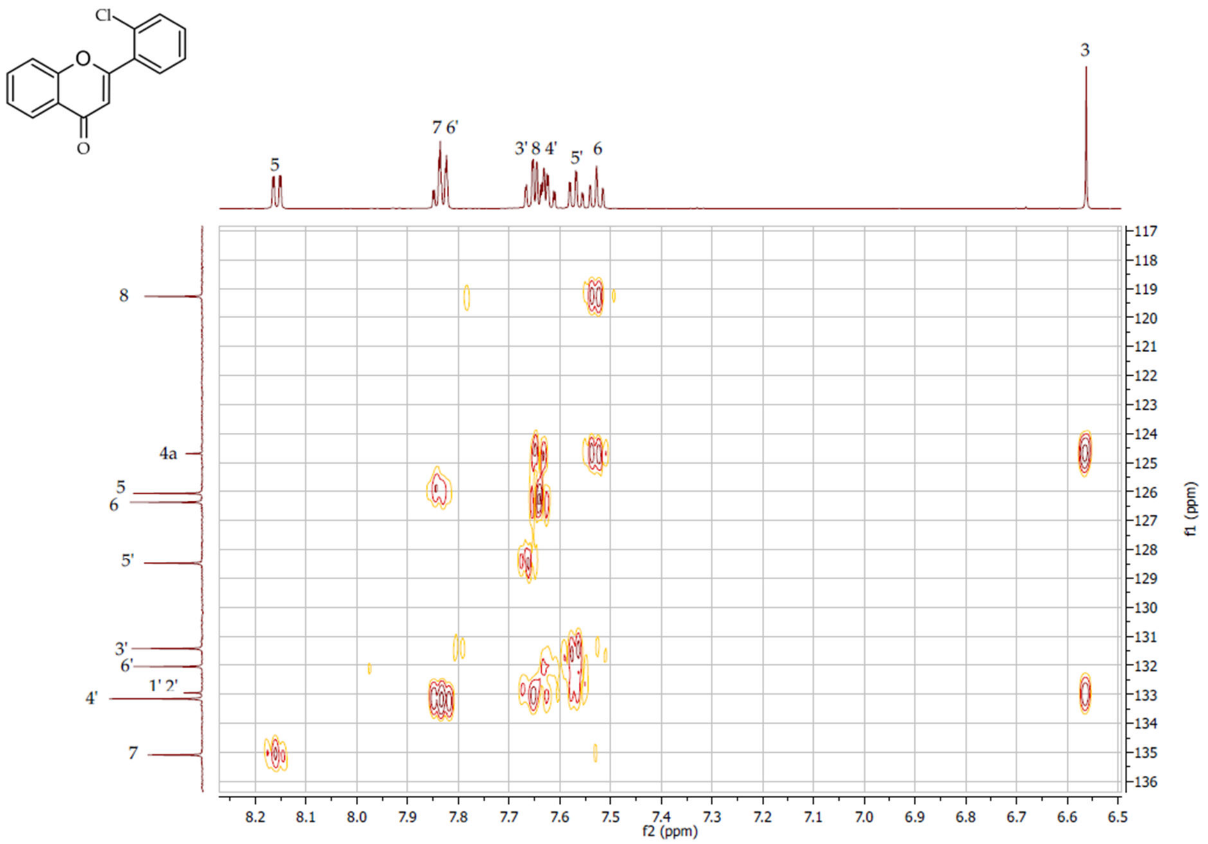

Figure S20. HMBC contour map – ^1H x ^{13}C expansion of 2'-chloroflavone (**D1**)

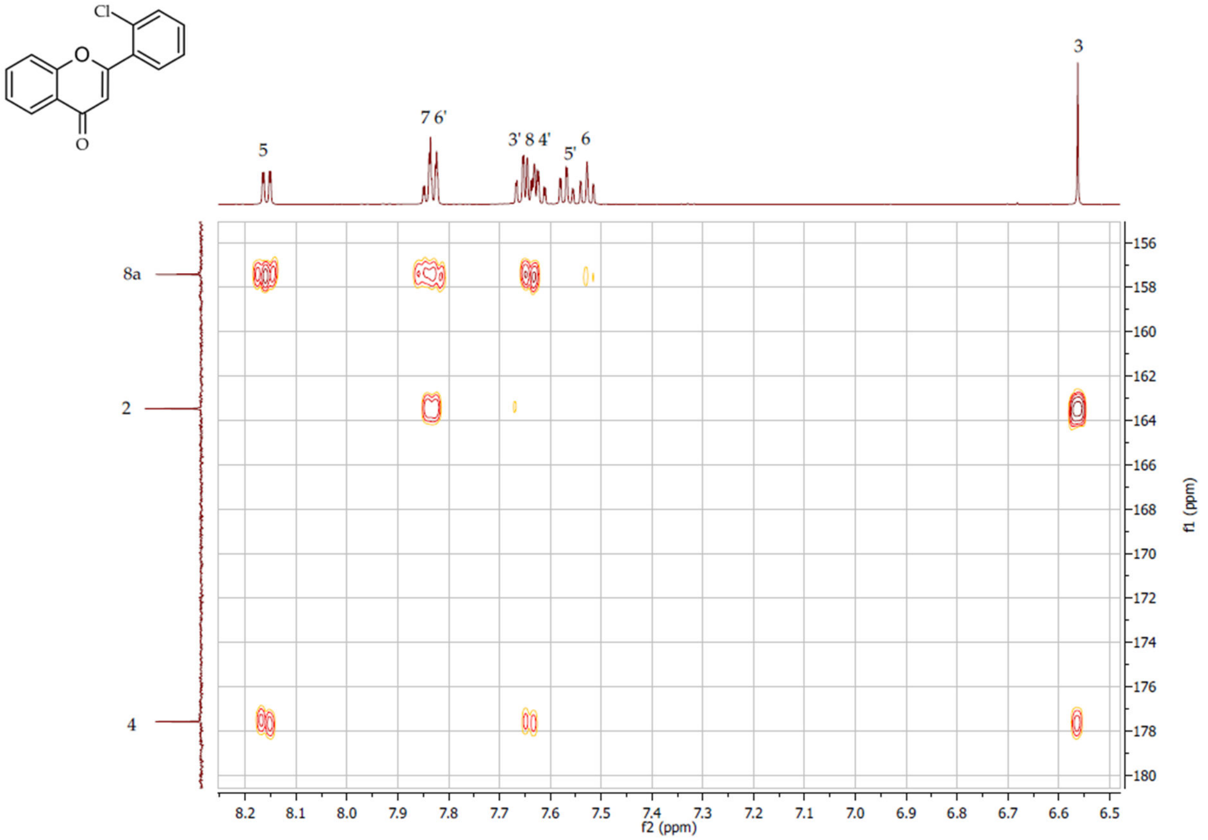

Figure S21. HMBC contour map – ^1H x ^{13}C expansion of 2'-chloroflavone (**D1**)

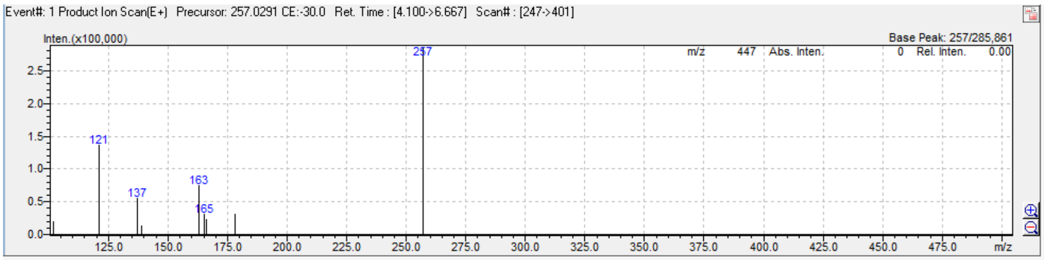

Figure S22. MS analysis of 3’-chloroflavone (**D2**)

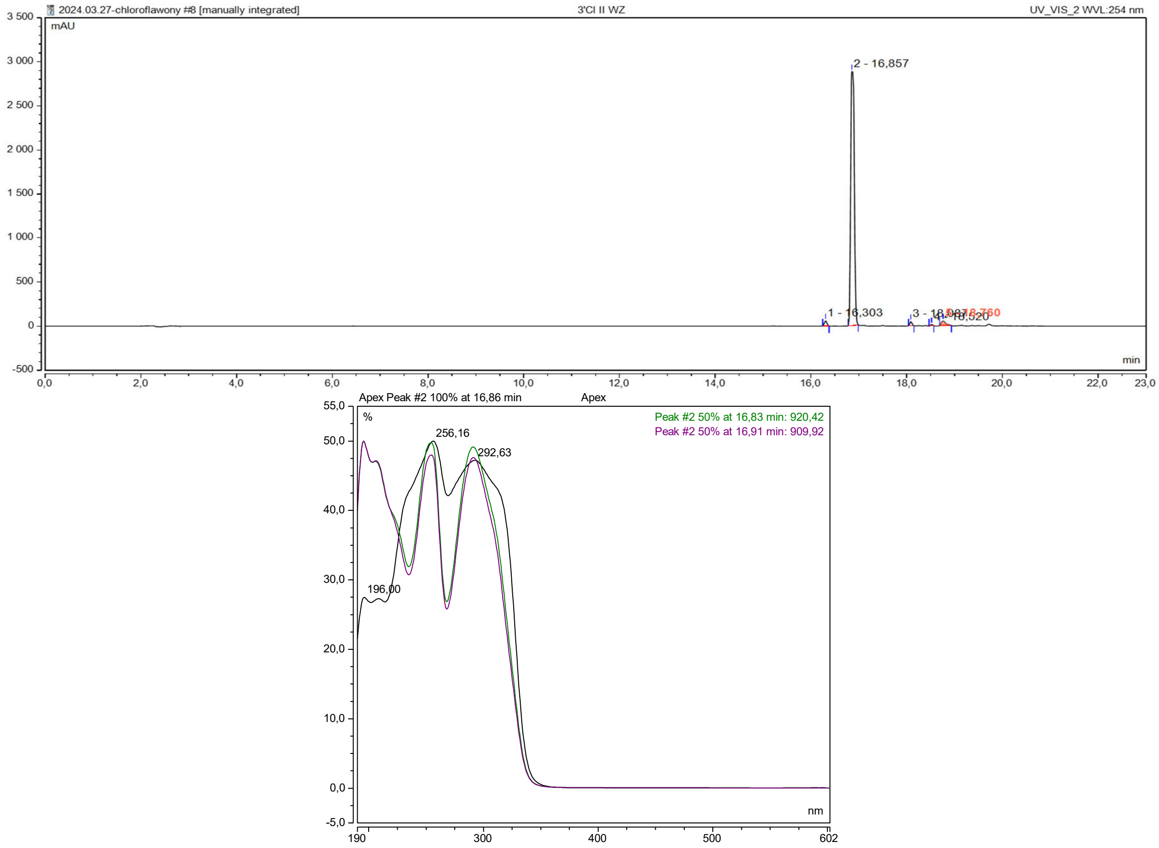

Peak	Peak Name	Ret.Time	Amount	Rel.Area	Area	Height	Type	Width (50%)	Asym.	Resol.	Plates
No.		min	n.a.	%	mAU*min	mAU		min	EP	EP	EP
UV_VIS_2	UV_VIS_2	UV_VIS_2	UV_VIS_2	UV_VIS_2	UV_VIS_2	UV_VIS_2	UV_VIS_2	UV_VIS_2	UV_VIS_2	UV_VIS_2	UV_VIS_2
1		16,303	n.a.	1,36	3,6146	59,90	BMB*	0,057	1,12	4,62	447792
2		16,857	n.a.	95,34	253,2738	2885,31	BMB*	0,084	1,30	10,81	223544
3		18,087	n.a.	0,93	2,4813	47,58	BMB*	0,050	1,15	5,00	715889
4		18,520	n.a.	0,31	0,8307	15,72	BMB*	0,052	0,99	1,96	702242
5		18,760	n.a.	2,05	5,4576	50,58	BMB*	0,092	1,72	n.a.	228473
Maximum			0,0000	95,34	253,2738	2885,31		0,092	1,72	10,81	715889
Minimum			0,0000	0,31	0,8307	15,72		0,050	0,99	1,96	223544
Sum			0,0000	100,00	265,6579	3059,10					

Figure S23. HPLC analysis of 3’-chloroflavone (**D2**)

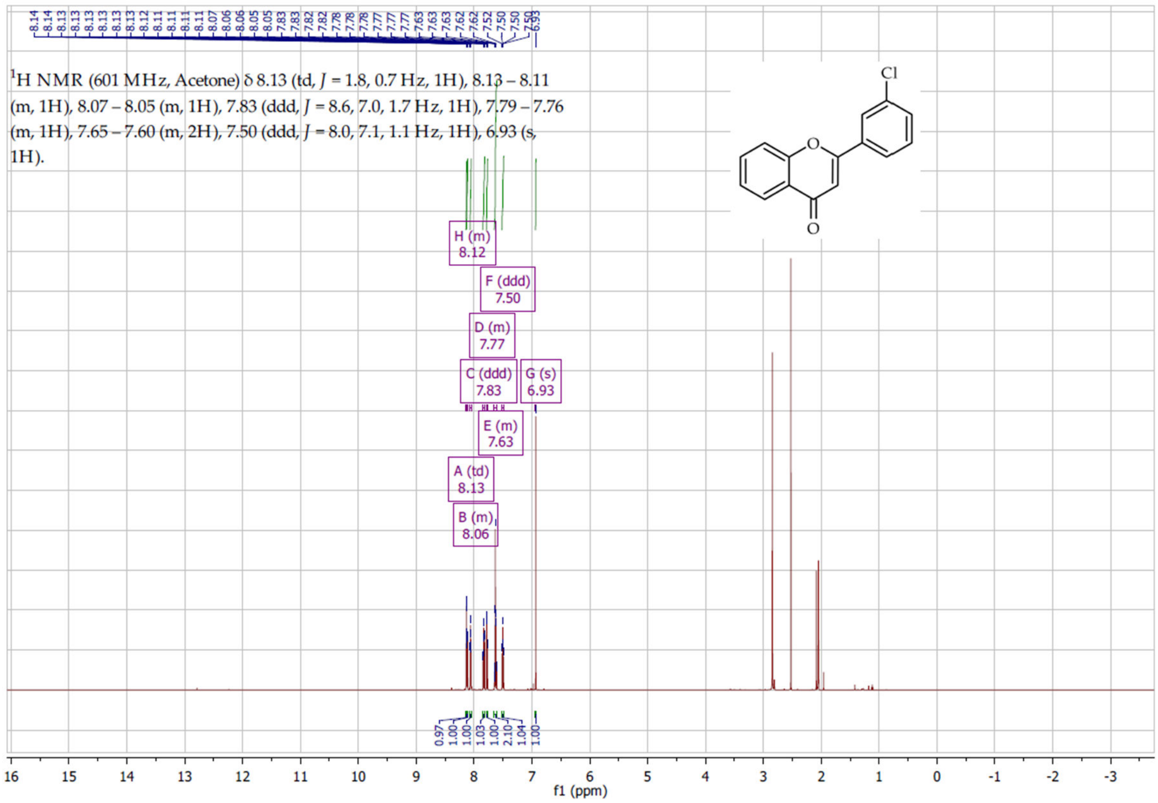

Figure S24. ^{1}H NMR spectrum (δ, acetone-d6, 600 MHz) of 3'-chloroflavone (**D2**)

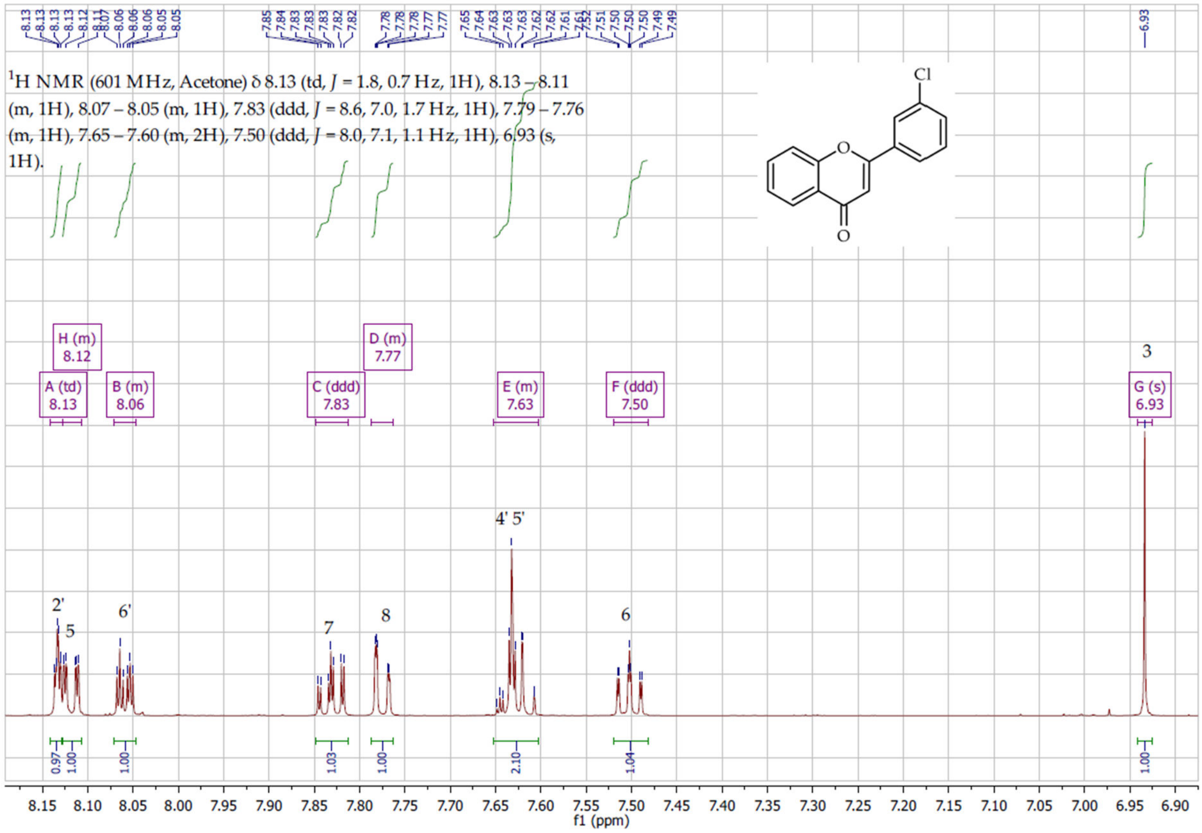

Figure S25. ^{1}H NMR spectrum expansion (δ, acetone-d6, 600 MHz) of 3'-chloroflavone (**D2**)

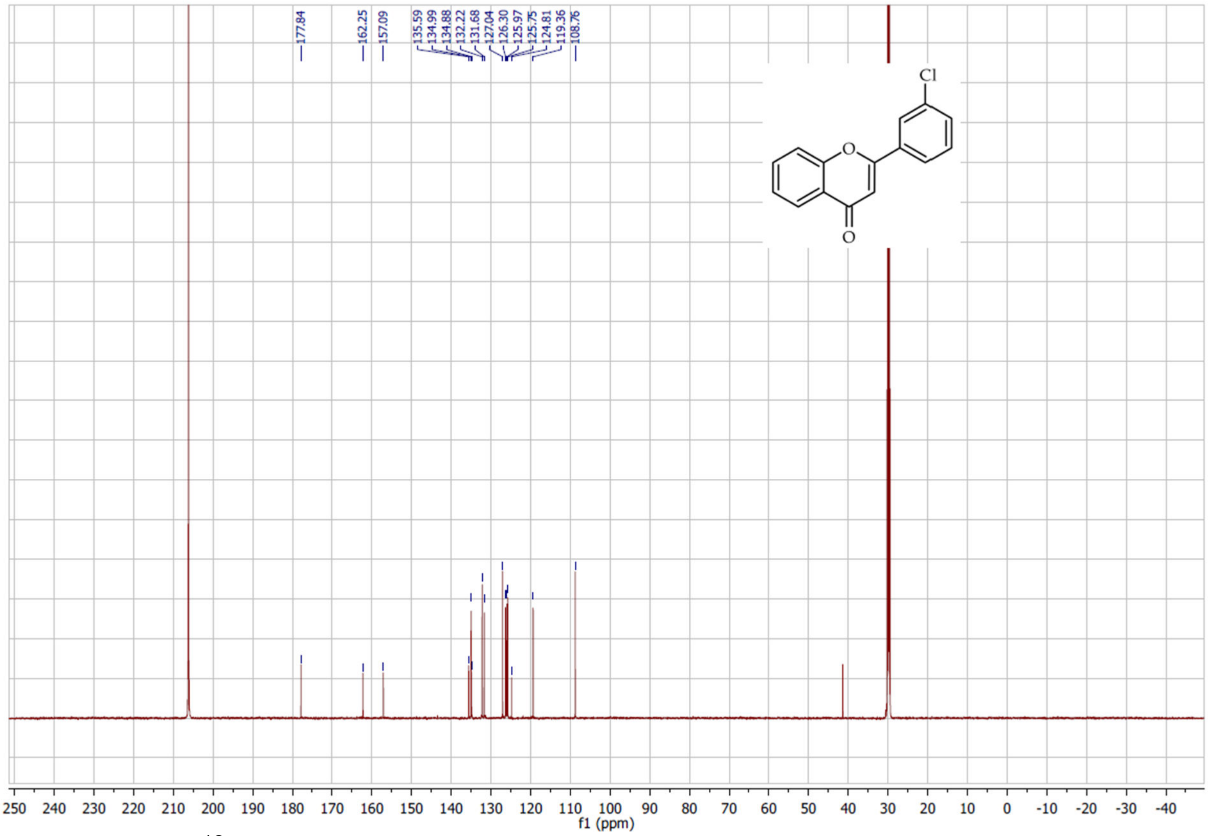

Figure S26. ^{13}C NMR spectrum (*δ,* acetone-d6, 151 MHz) of 3’-chloroflavone (**D2**)

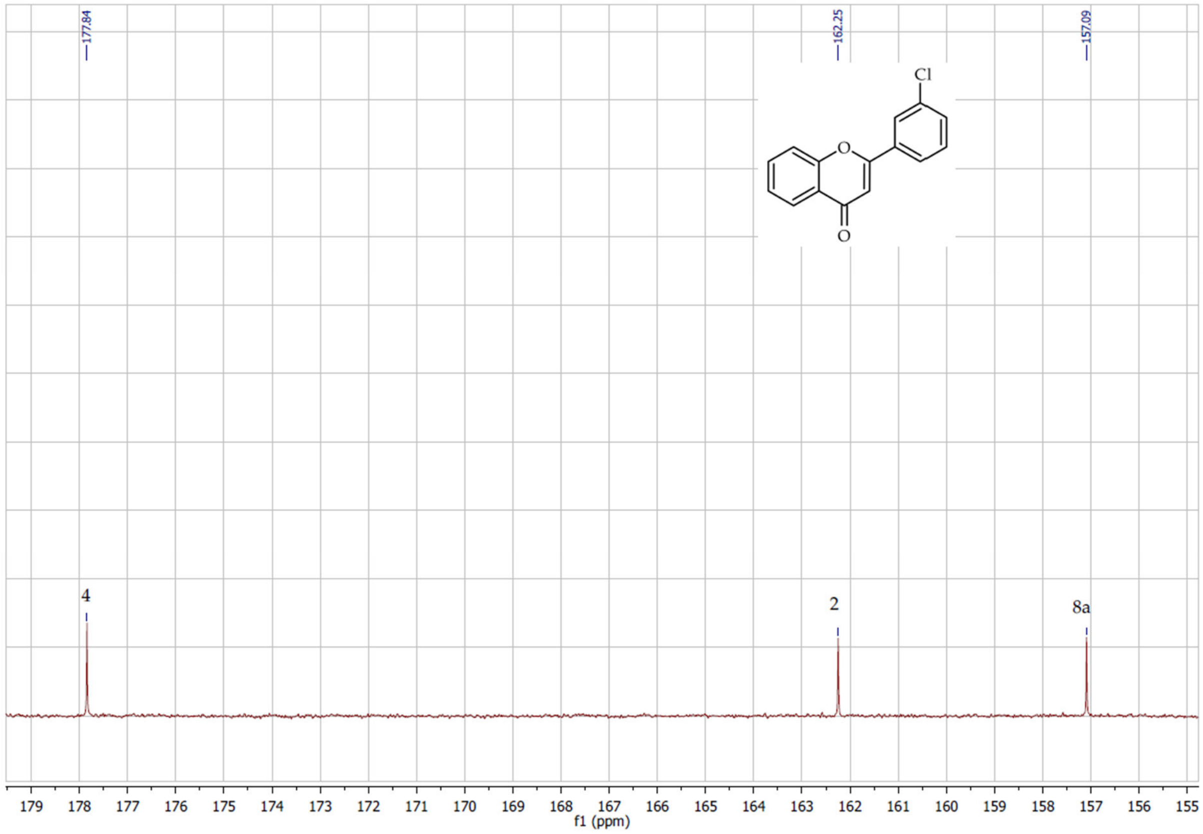

Figure S27. ^{13}C NMR spectrum expansion (*δ,* acetone-d6, 151 MHz) of 3’-chloroflavone (**D2**)

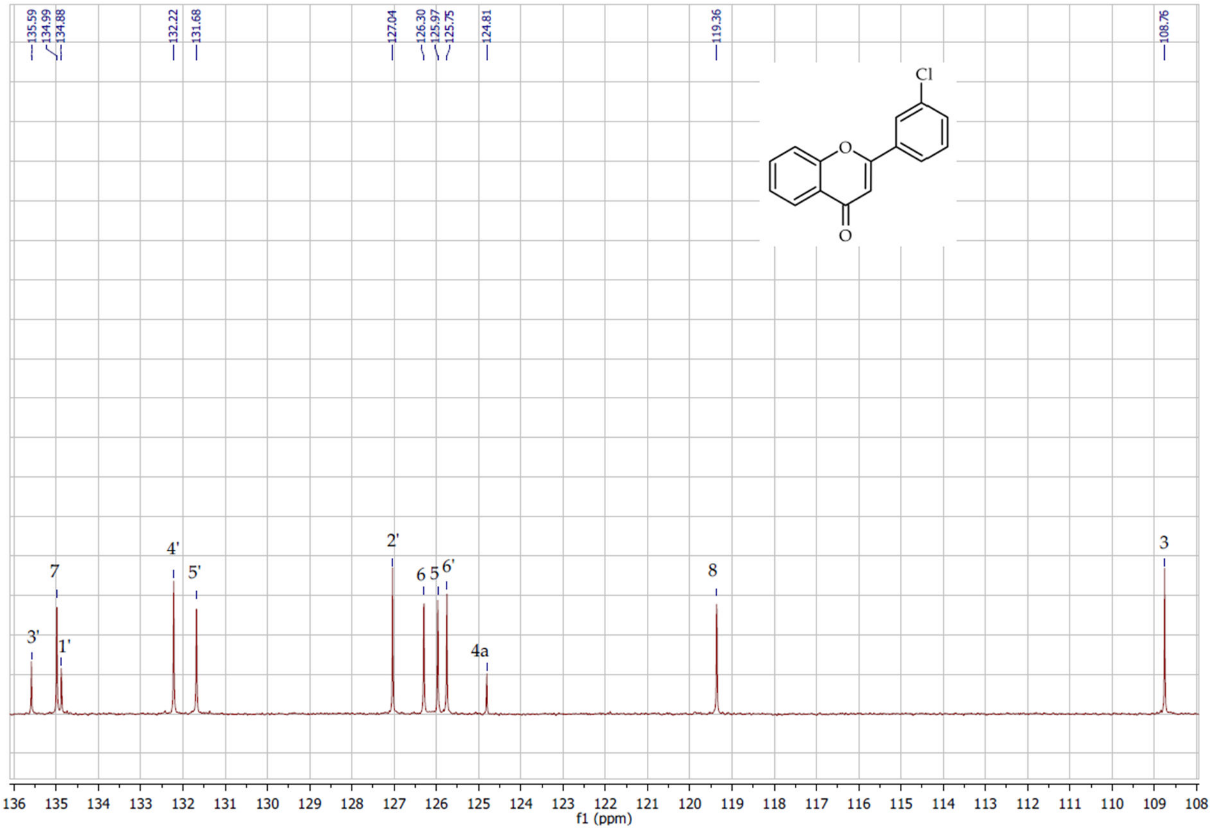

Figure S28. ^{13}C NMR spectrum expansion (*δ,* acetone-d6, 151 MHz) of 3'-chloroflavone (**D2**)

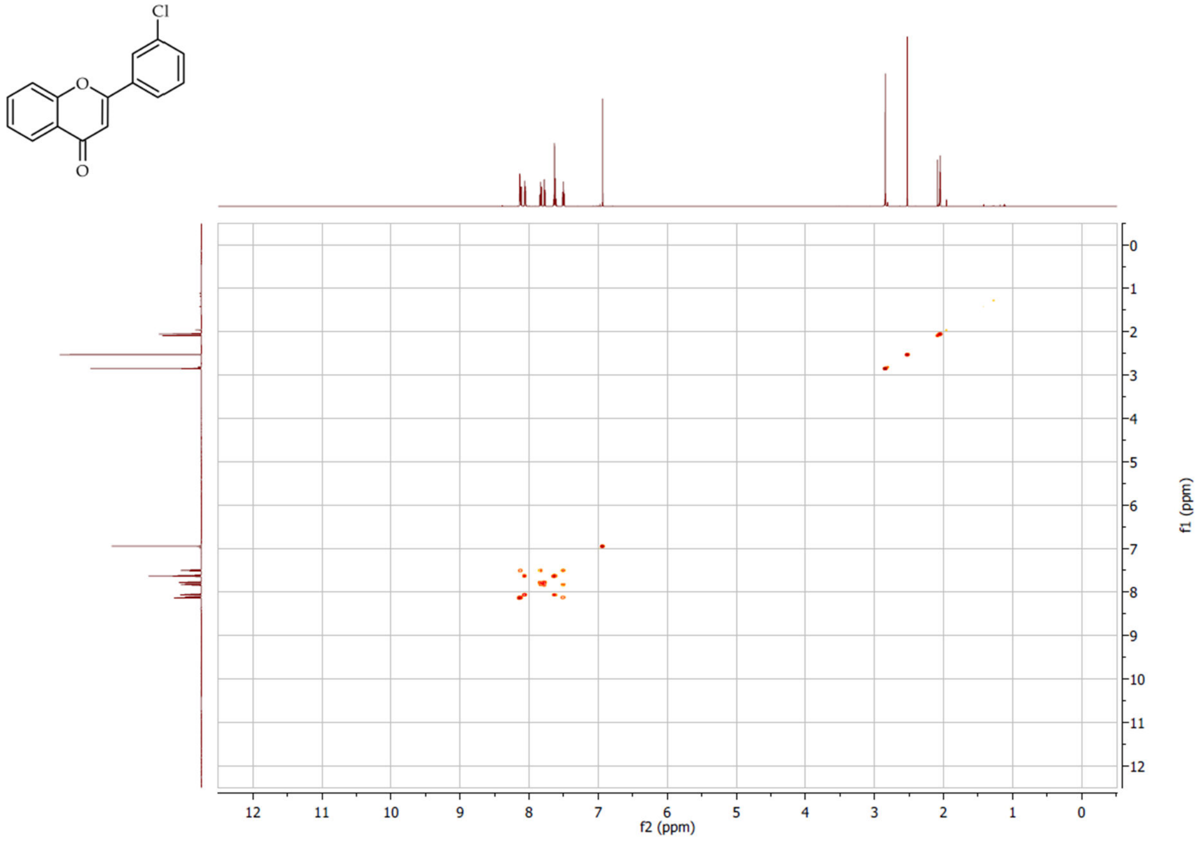

Figure S29. COSY contour map – ^{1}H x ^{1}H of 3'-chloroflavone (**D2**)

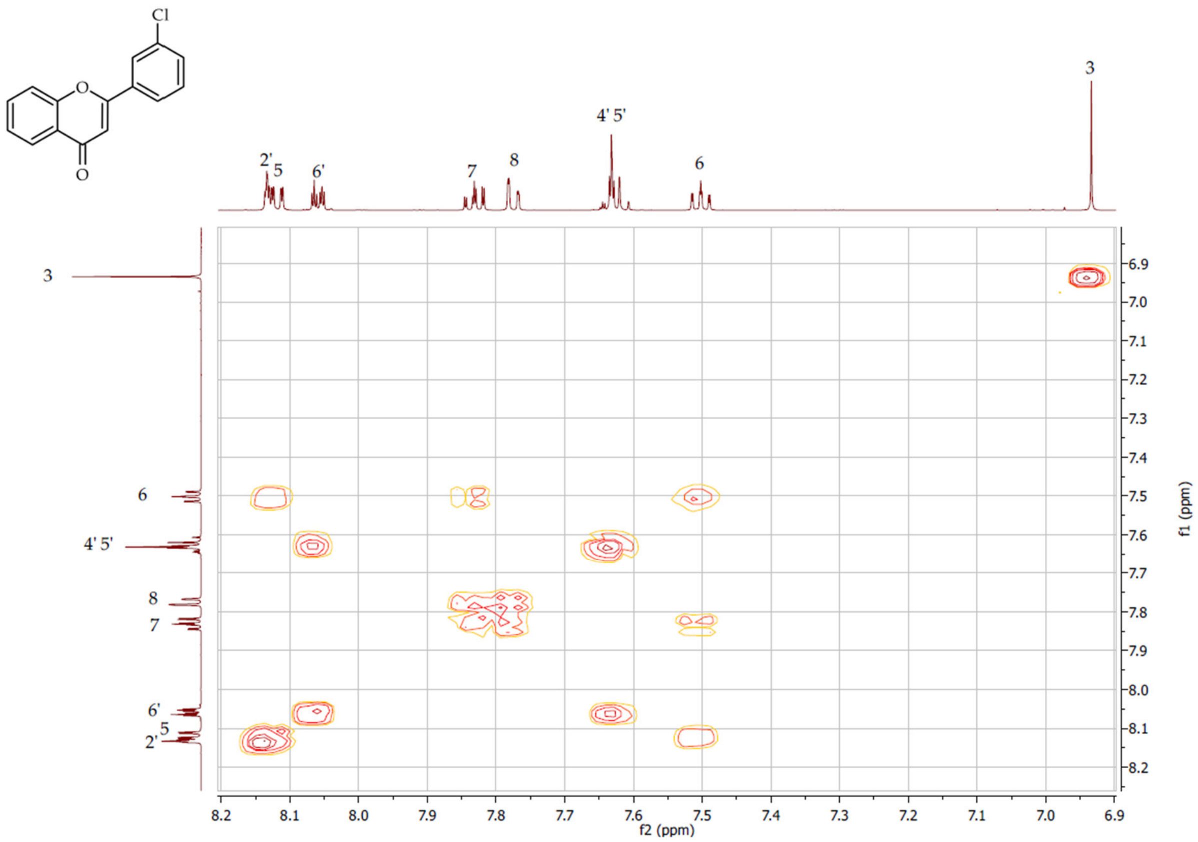

Figure S30. COSY contour map – ^1H x ^1H expansion of 3’-chloroflavone (**D2**)

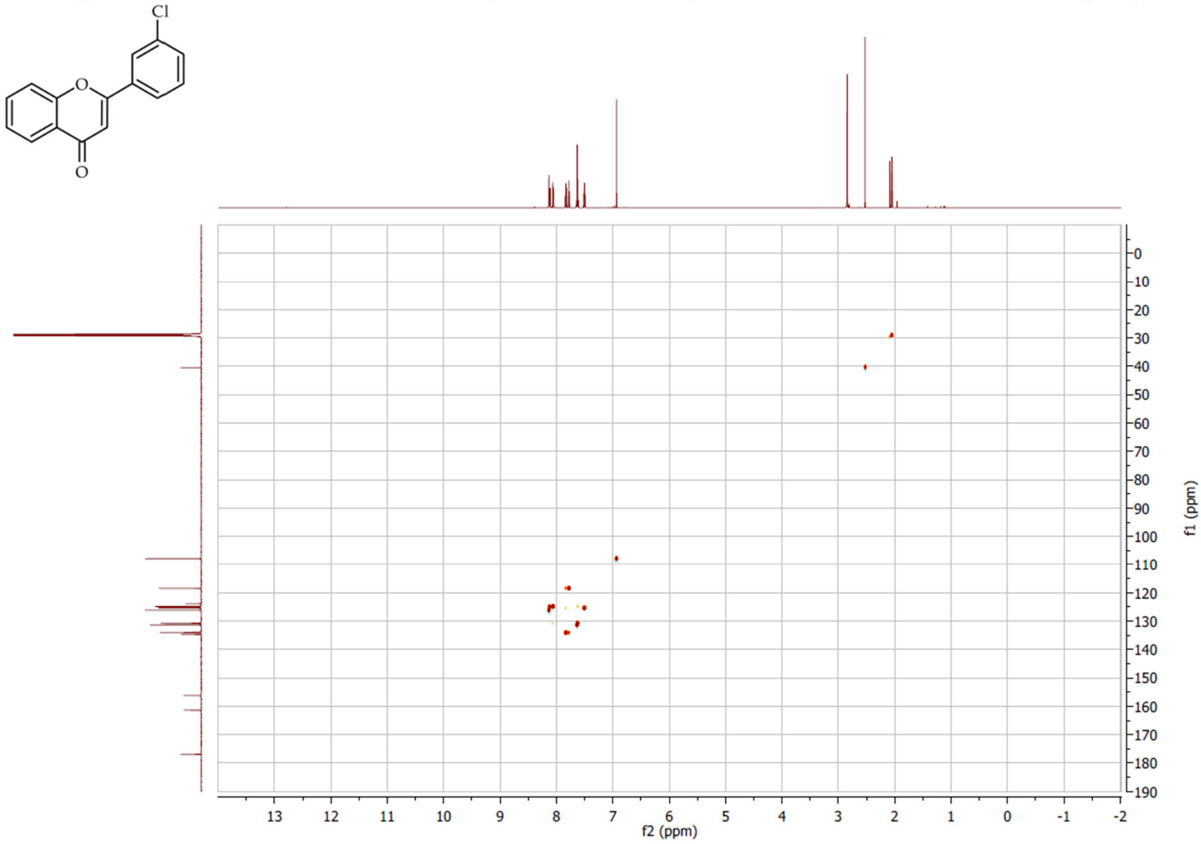

Figure S31. HMQC contour map – ^1H x ^{13}C of 3’-chloroflavone (**D2**)

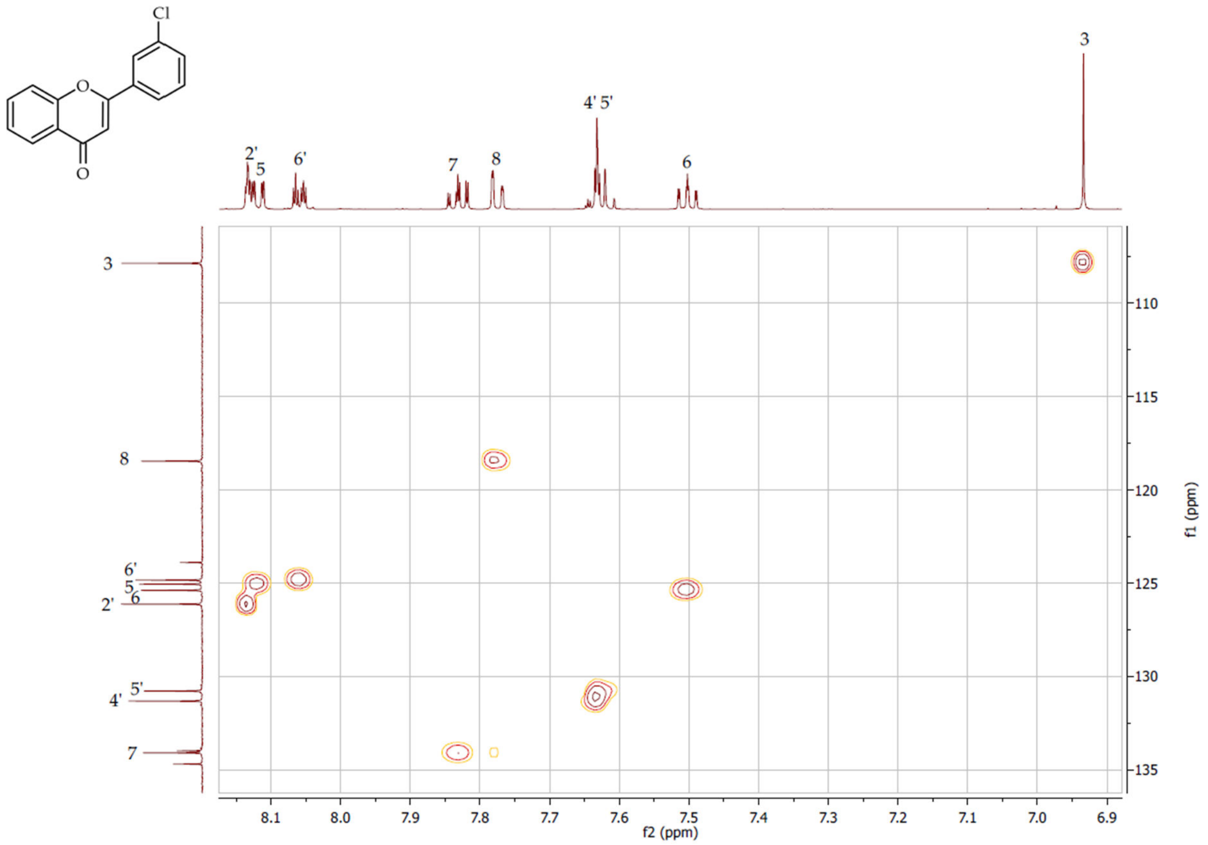

Figure S32. HMQC contour map – ^{1}H x ^{13}C expansion of 3'-chloroflavone (**D2**)

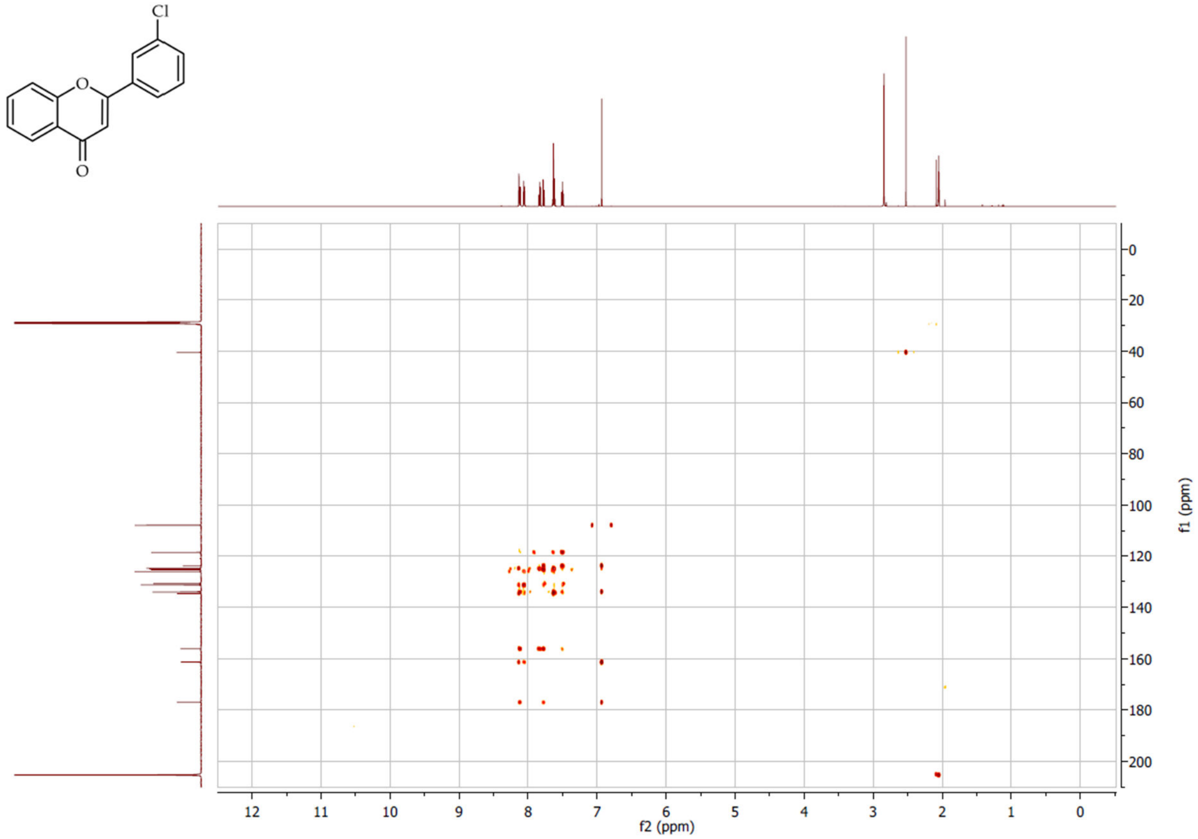

Figure S33. HMBC contour map – ^{1}H x ^{13}C of 3'-chloroflavone (**D2**)

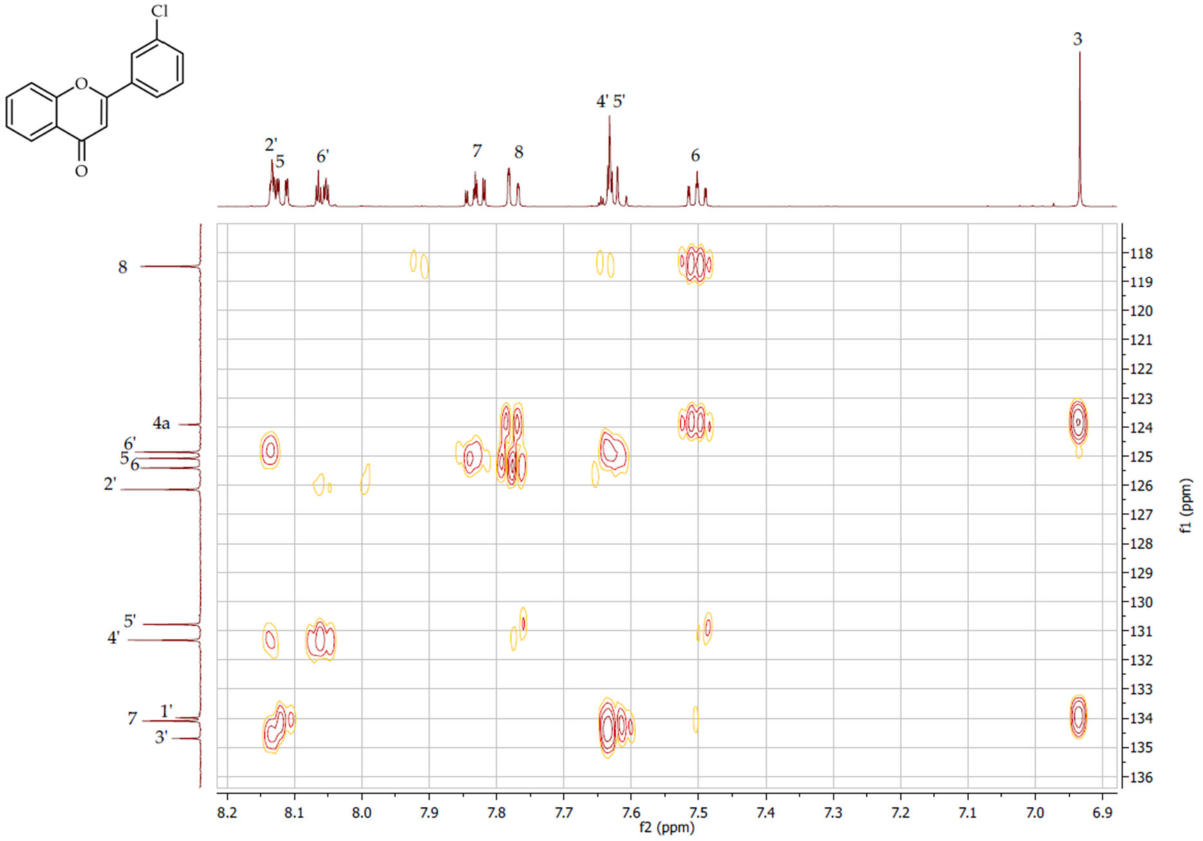

Figure S34. HMBC contour map – ^{1}H x ^{13}C expansion of 3’-chloroflavone (**D2**)

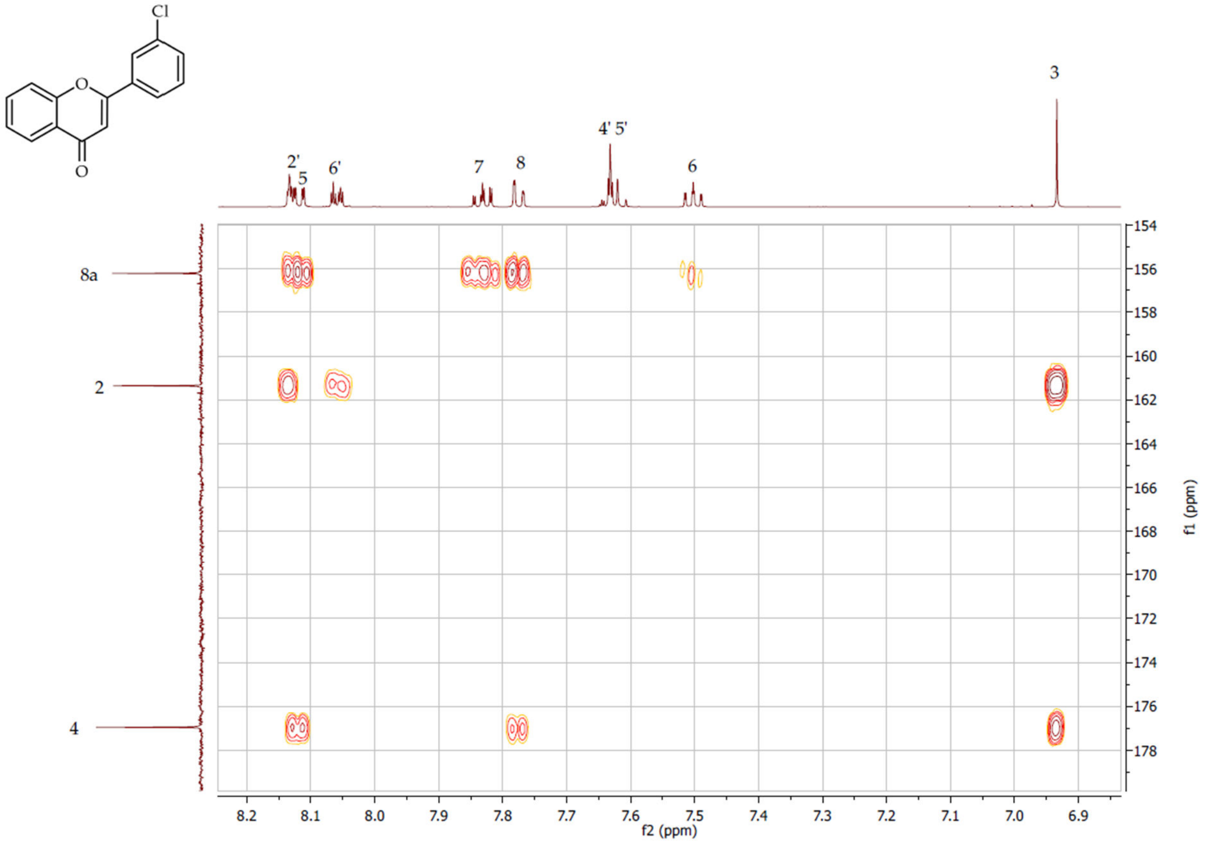

Figure S35. HMBC contour map – ^{1}H x ^{13}C expansion of 3’-chloroflavone (**D2**)

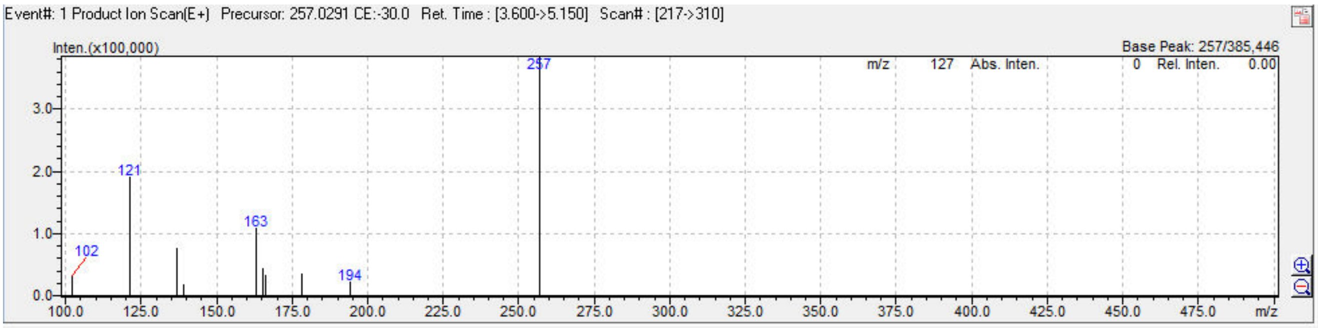

Figure S36. MS analysis of 4'-chloroflavone (**D3**)

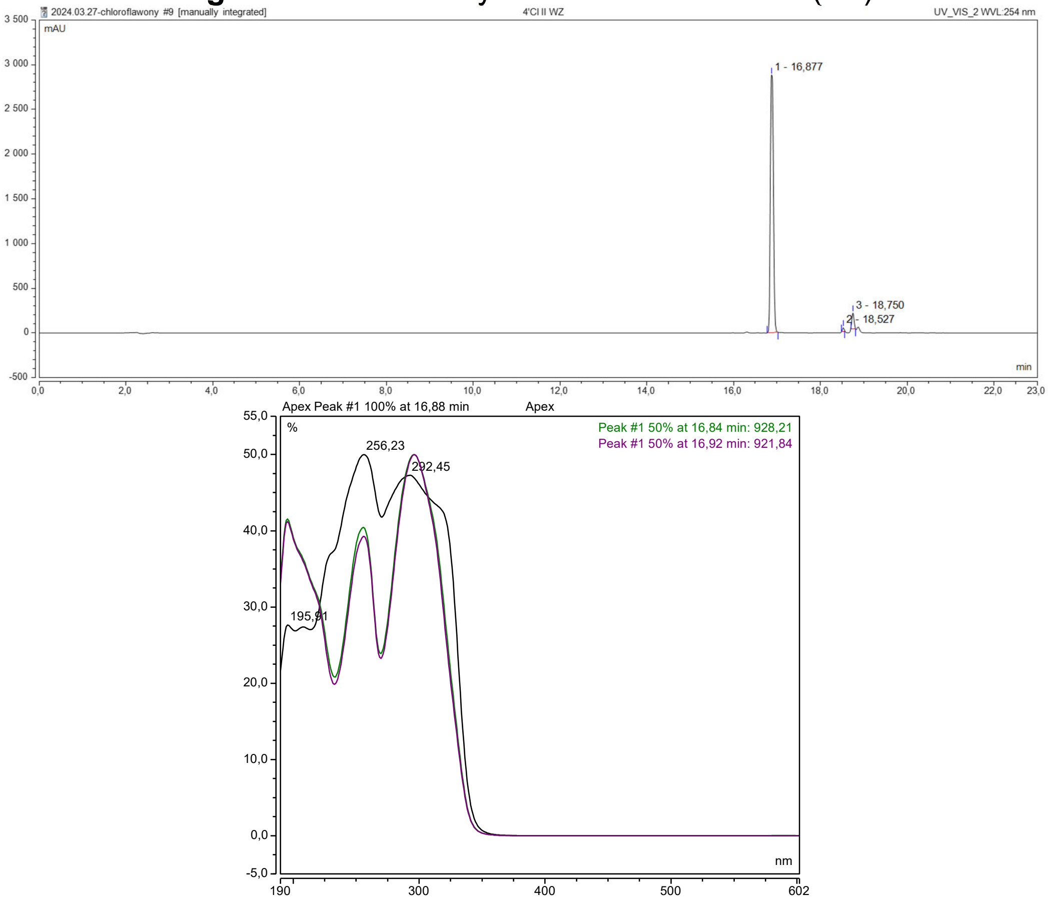

Peak	Peak Name	Ret.Time	Amount	Rel.Area	Area	Height	Type	Width (50%)	Asym.	Resol.	Plates
No.		min	n.a.	%	mAU*min	mAU		min	EP	EP	EP
UV_VIS_2	UV_VIS_2	UV_VIS_2	UV_VIS_2	UV_VIS_2	UV_VIS_2	UV_VIS_2	UV_VIS_2	UV_VIS_2	UV_VIS_2	UV_VIS_2	UV_VIS_2
1		16,877	n.a.	95,16	237,6980	2883,30	BMB*	0,078	1,19	15,79	256705
2		18,527	n.a.	0,74	1,8555	42,03	BMB*	0,045	1,04	2,58	944409
3		18,750	n.a.	4,10	10,2463	178,70	BMB*	0,057	1,05	n.a.	597065
Maximum			0,0000	95,16	237,6980	2883,30		0,078	1,19	15,79	944409
Minimum			0,0000	0,74	1,8555	42,03		0,045	1,04	2,58	256705
Sum			0,0000	100,00	249,7998	3104,02					

Figure S37. HPLC analysis of 4'-chloroflavone (**D3**)

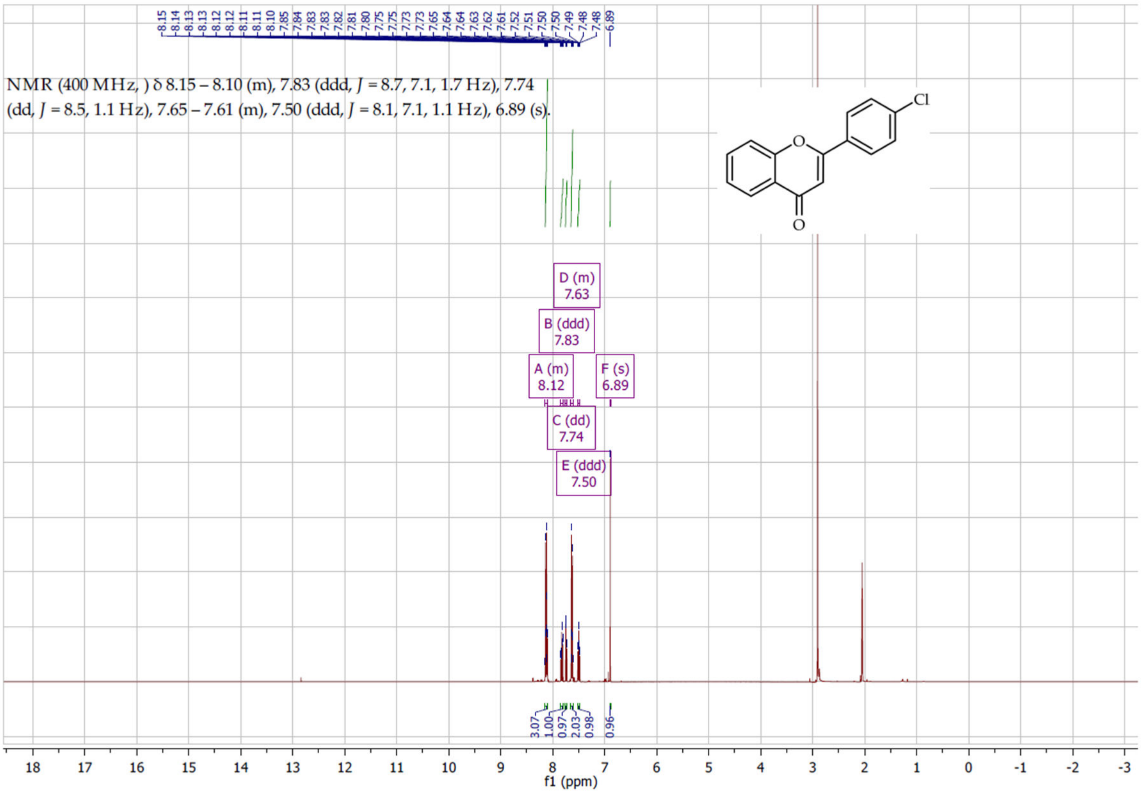

Figure S38. ^{1}H NMR spectrum (δ, acetone-d6, 600 MHz) of 4'-chloroflavone (**D3**)

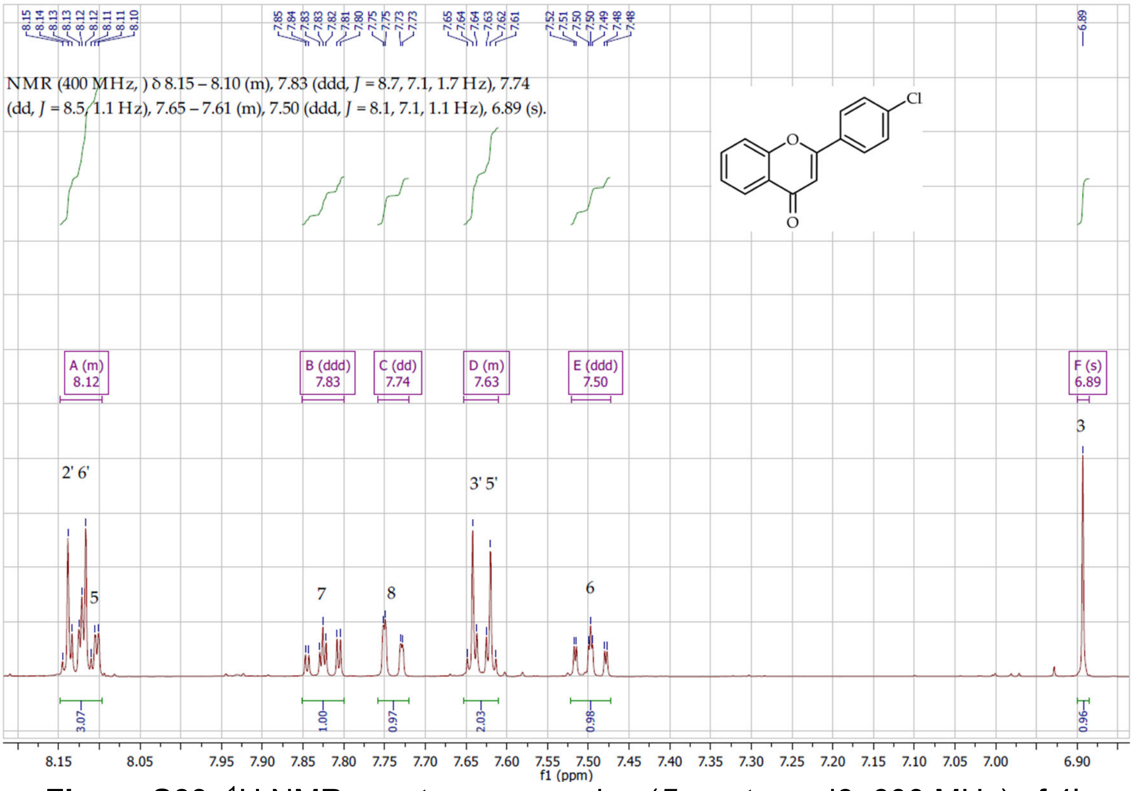

Figure S39. ^{1}H NMR spectrum expansion (δ, acetone-d6, 600 MHz) of 4'-chloroflavone (**D3**)

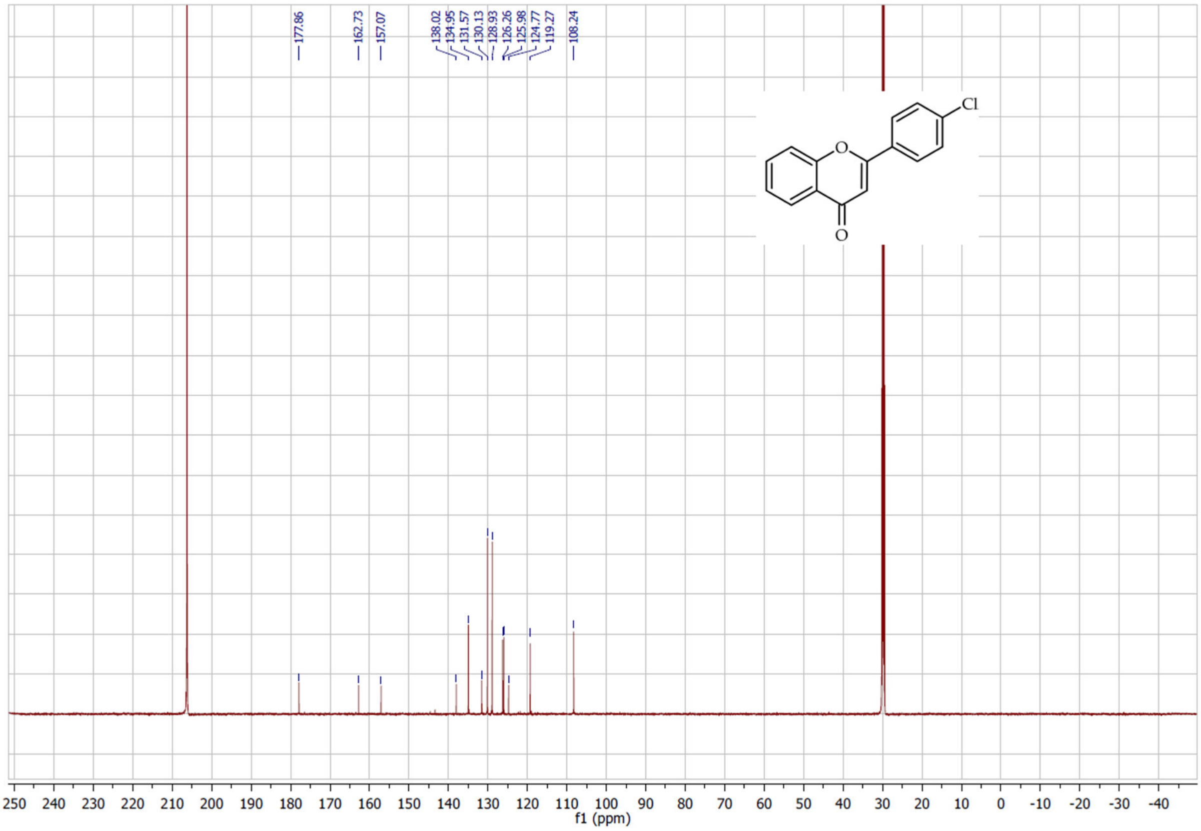

Figure S40. ^{13}C NMR spectrum (*δ,* acetone-d6, 151 MHz) of 4'-chloroflavone (**D3**)

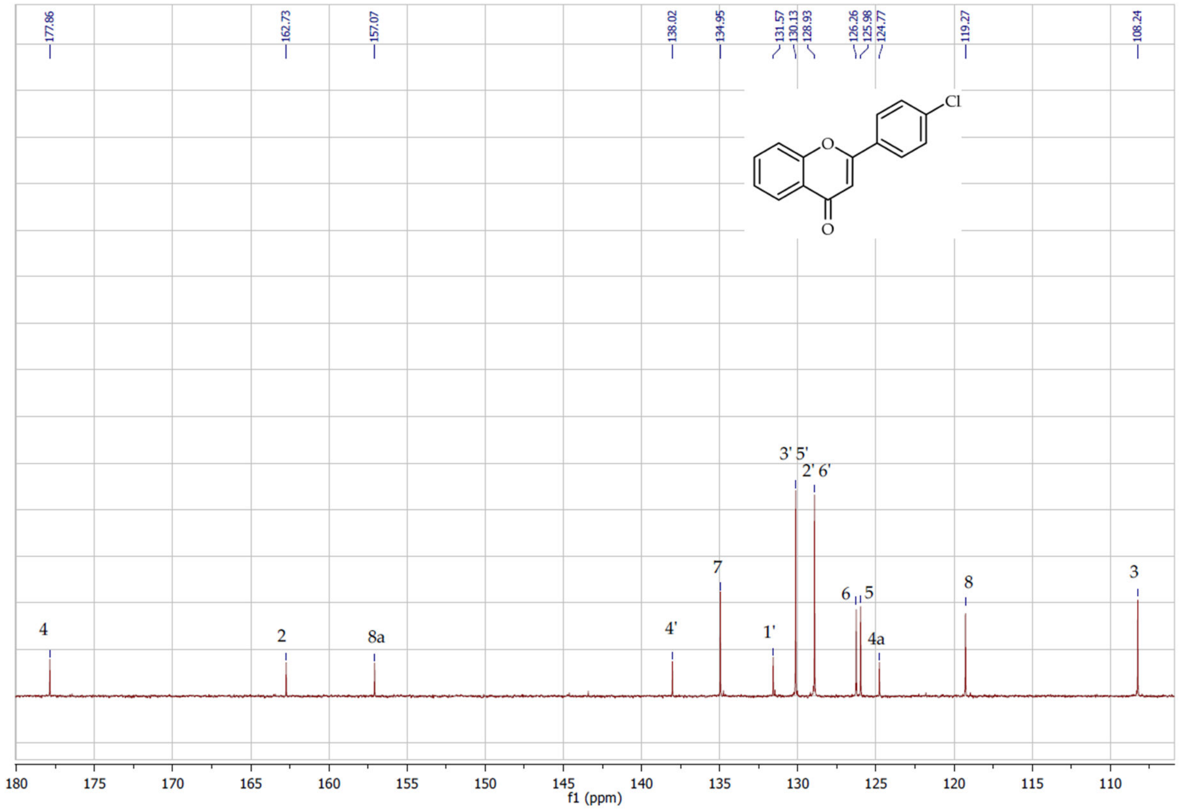

Figure S41. ^{13}C NMR spectrum expansion (*δ,* acetone-d6, 151 MHz) of 4'-chloroflavone (**D3**)

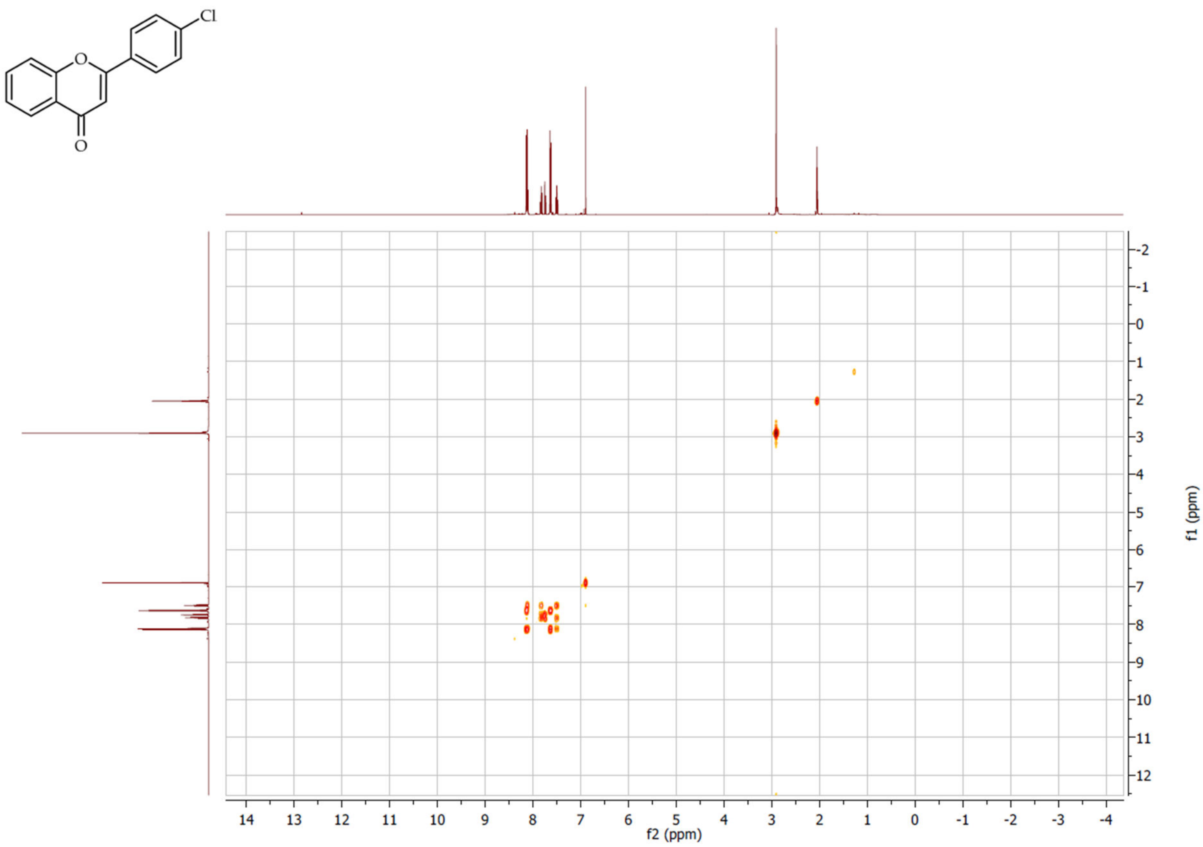

Figure S42. COSY contour map – ^1H x ^1H of 4'-chloroflavone (**D3**)

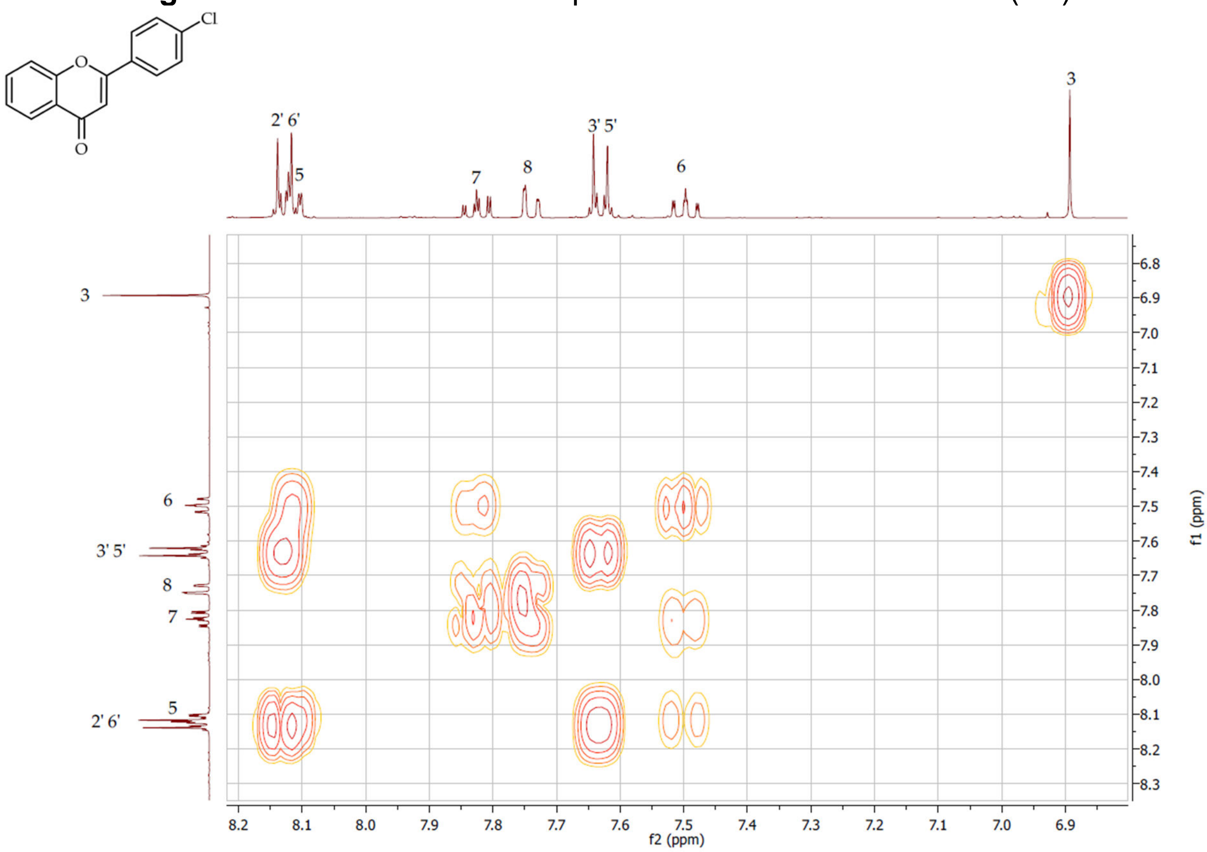

Figure S43. COSY contour map – ^1H x ^1H expansion of 4'-chloroflavone (**D3**)

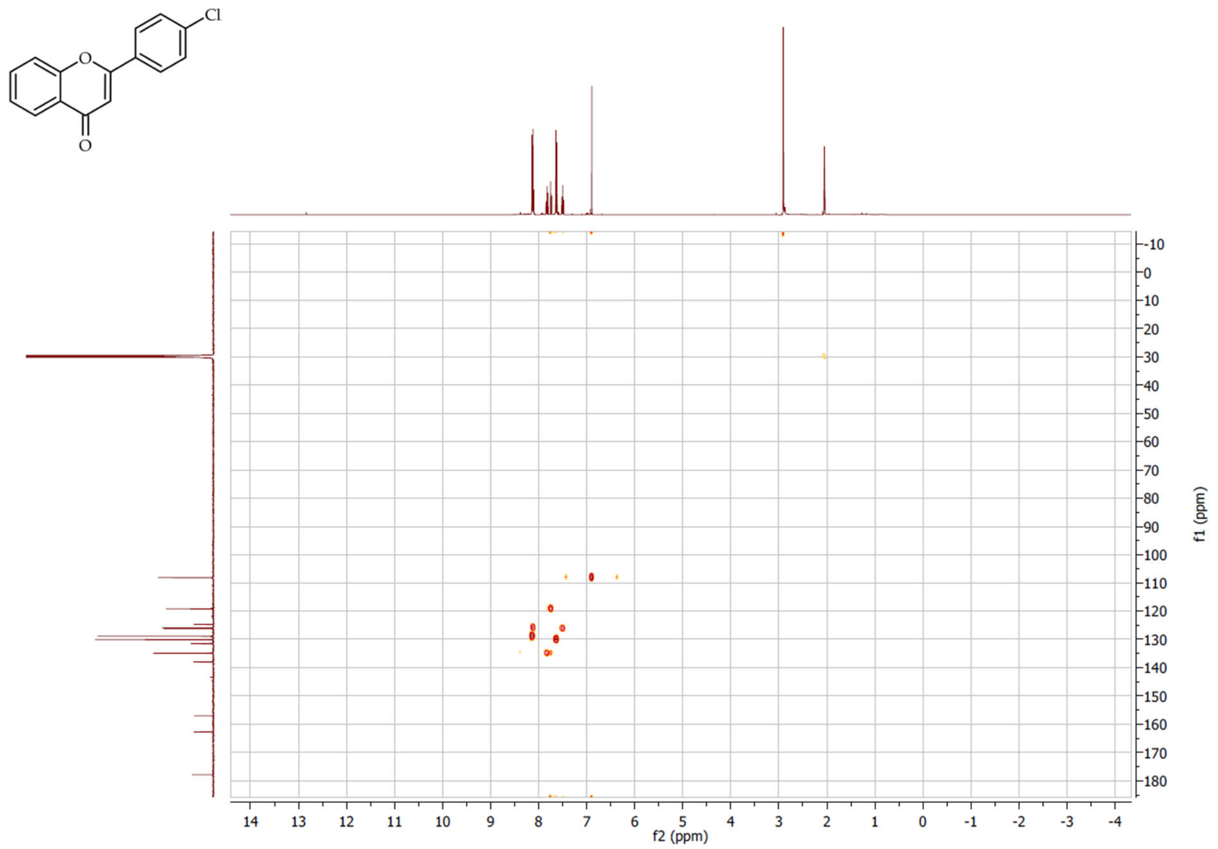

Figure S44. HMQC contour map – ^{1}H x ^{13}C of 4'-chloroflavone (**D3**)

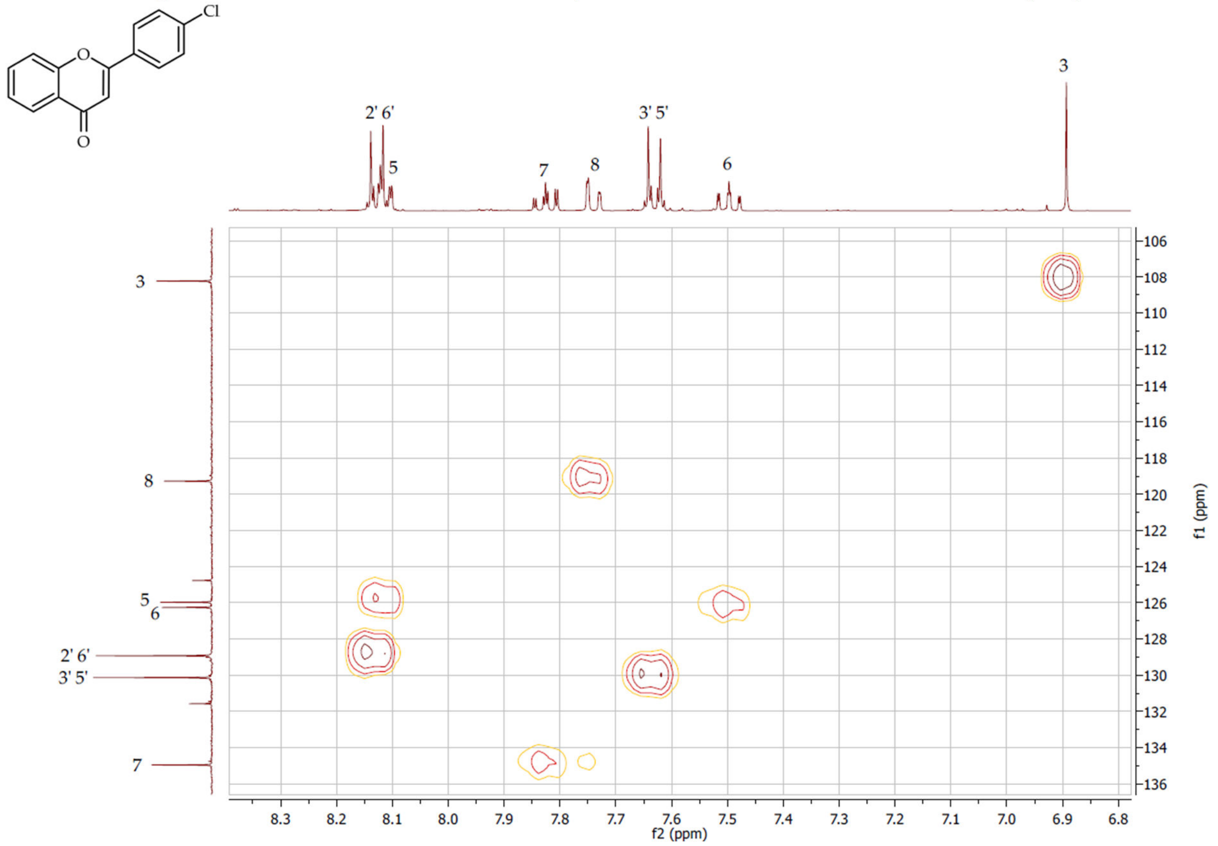

Figure S45. HMQC contour map – ^{1}H x ^{13}C expansion of 4'-chloroflavone (**D3**)

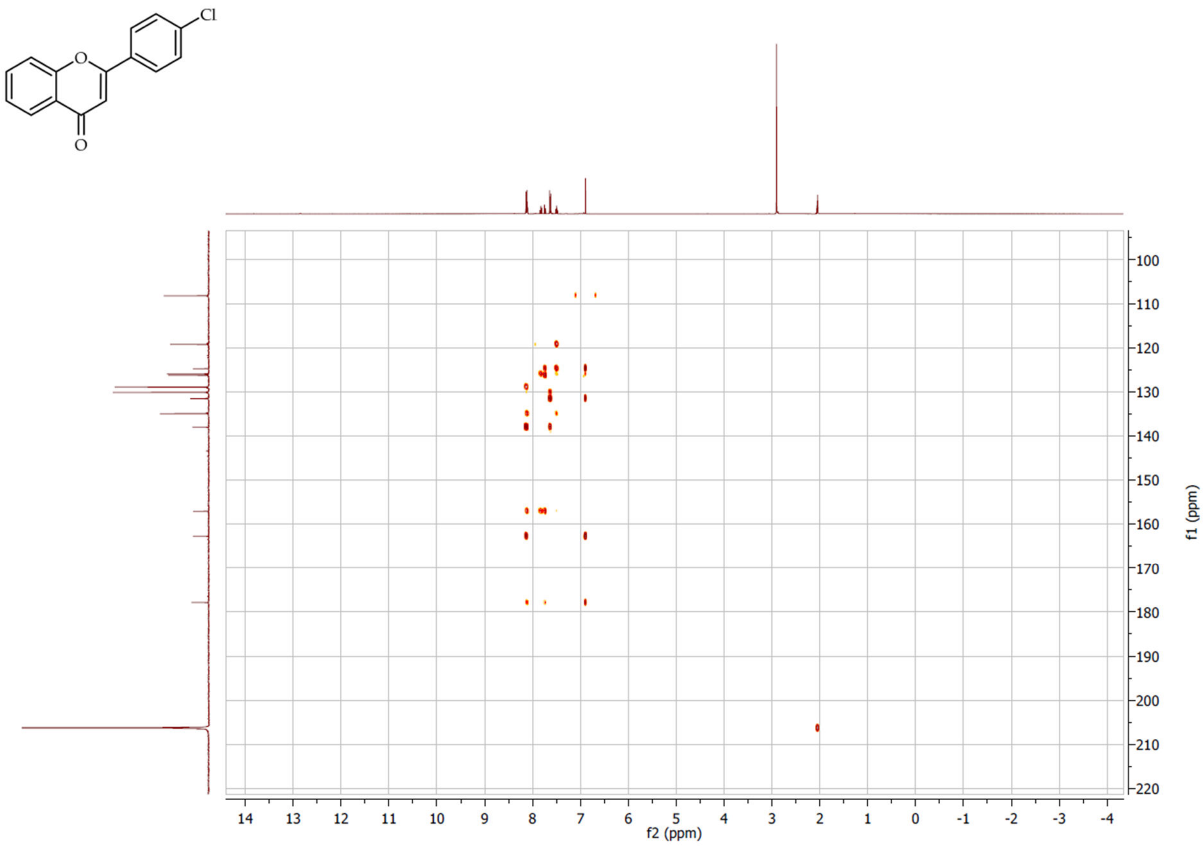

Figure S46. HMBC contour map – ^{1}H x ^{13}C of 4’-chloroflavone (**D3**)

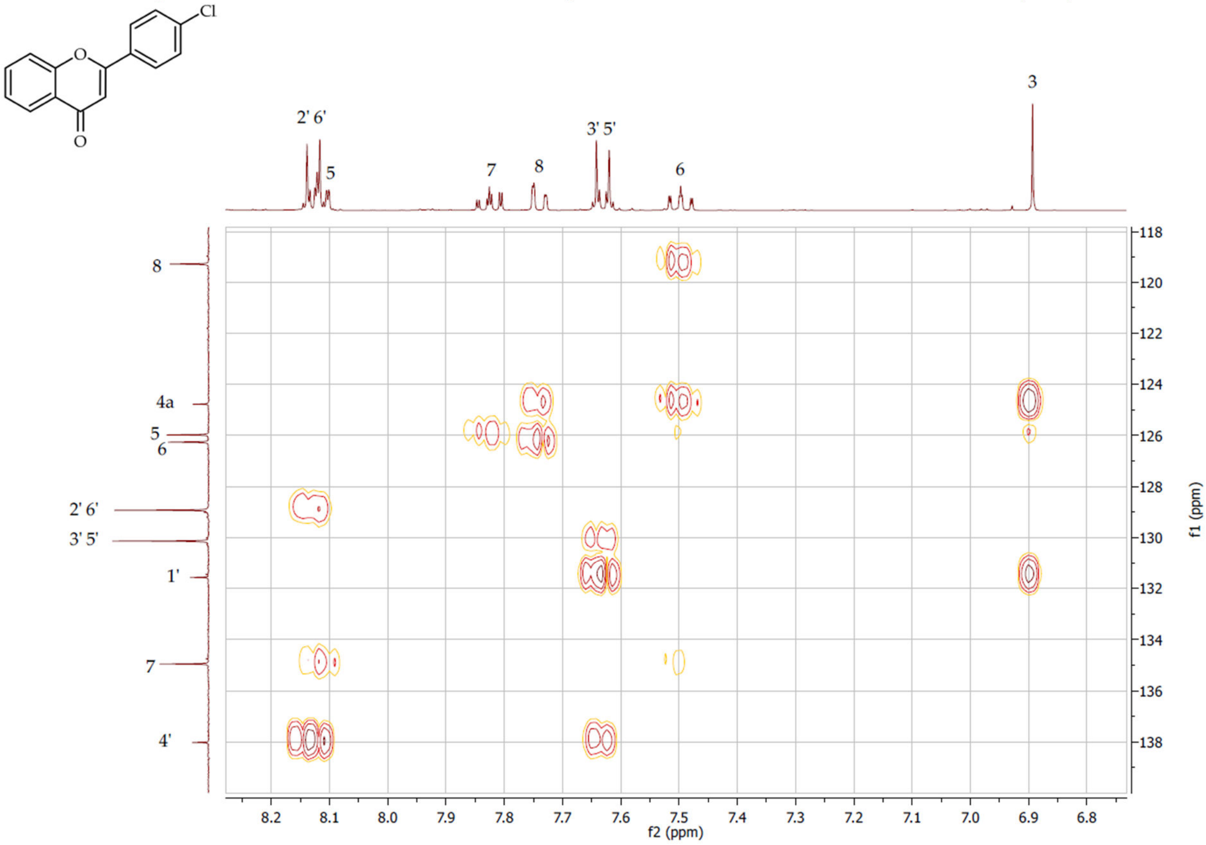

Figure S47. HMBC contour map – ^{1}H x ^{13}C expansion of 4’-chloroflavone (**D3**)

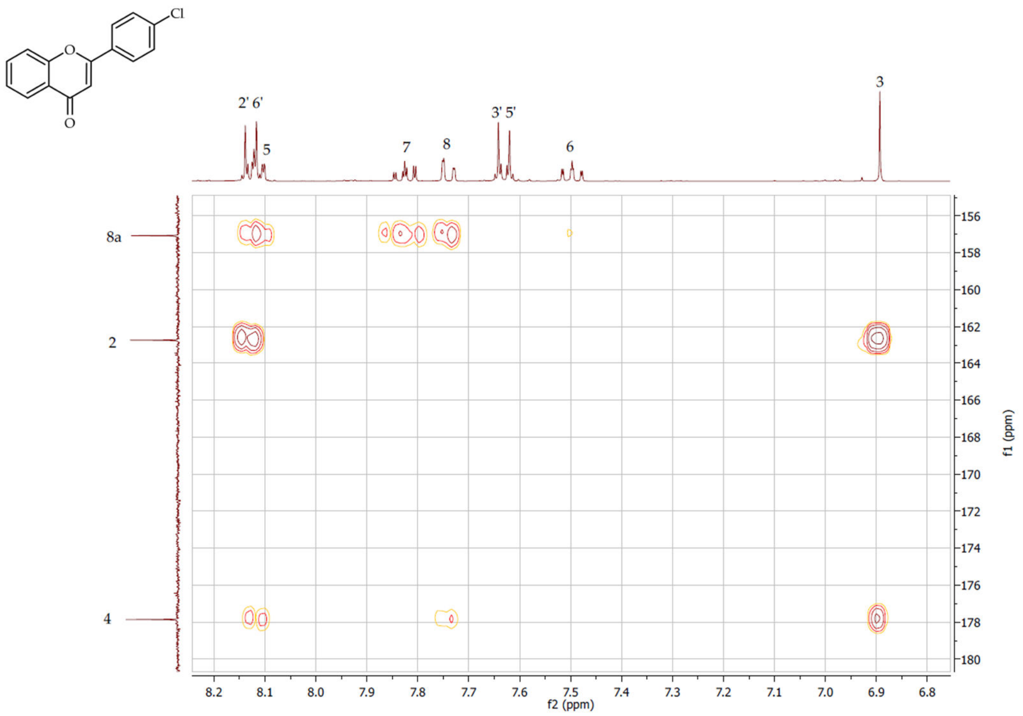

Figure S48. HMBC contour map – ^{1}H x ^{13}C expansion of 4'-chloroflavone (**D3**)

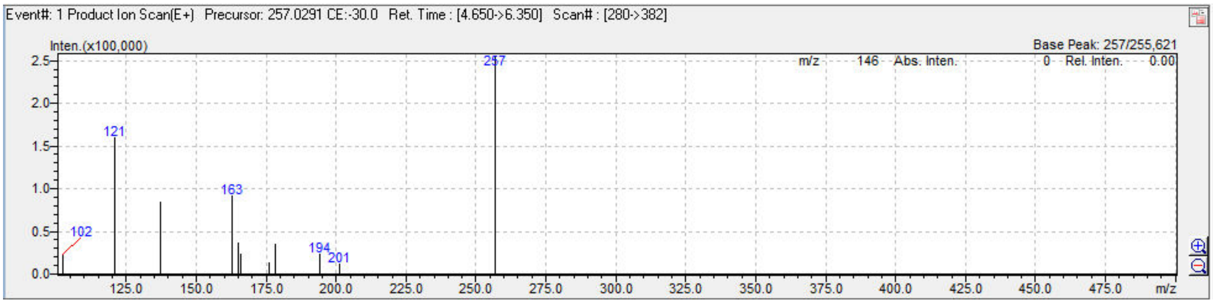

Figure S49. MS analysis of 6-chloroflavone (**D4**)

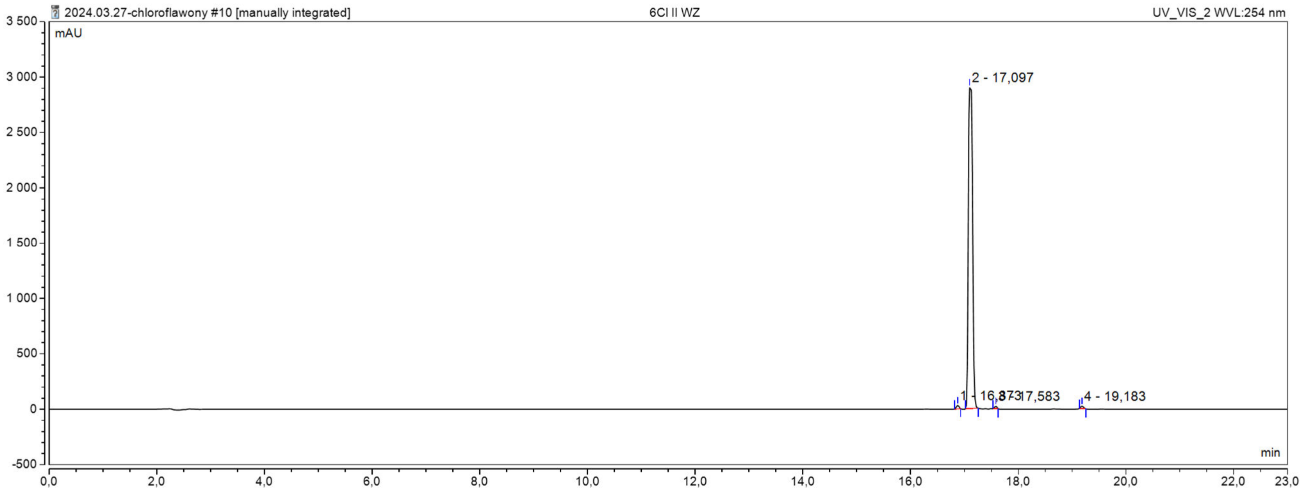

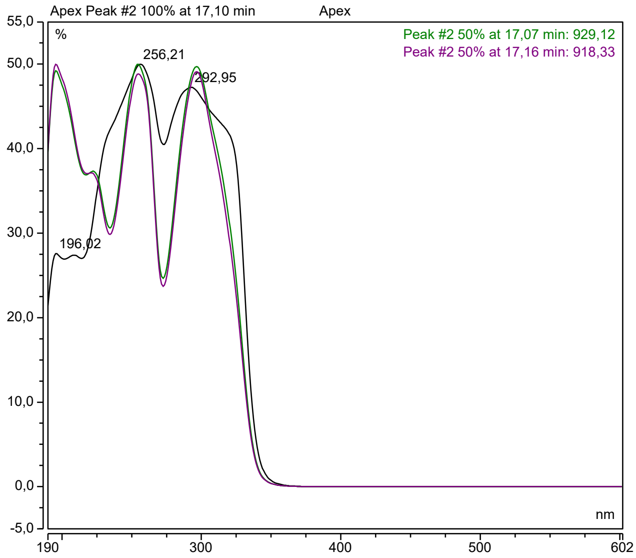

Peak	Peak Name	Ret.Time	Amount	Rel.Area	Area	Height	Type	Width (50%)	Asym.	Resol.	Plates
No.		min	n.a.	%	mAU*min	mAU		min	EP	EP	EP
UV_VIS_2	UV_VIS_2	UV_VIS_2	UV_VIS_2	UV_VIS_2	UV_VIS_2	UV_VIS_2	UV_VIS_2	UV_VIS_2	UV_VIS_2	UV_VIS_2	UV_VIS_2
1		16,873	n.a.	0,69	1,8396	31,90	BMB*	0,056	1,00	1,84	497509
2		17,097	n.a.	98,33	262,5413	2896,19	BMB*	0,087	1,40	4,30	214332
3		17,583	n.a.	0,44	1,1847	24,69	BMB*	0,047	0,98	17,14	784489
4		19,183	n.a.	0,53	1,4244	22,32	BMB*	0,063	1,17	n.a.	506607
Maximum			0,0000	98,33	262,5413	2896,19		0,087	1,40	17,14	784489
Minimum			0,0000	0,44	1,1847	22,32		0,047	0,98	1,84	214332
Sum			0,0000	100,00	266,9900	2975,10					

Figure S50. HPLC analysis of 6-chloroflavone (**D4**)

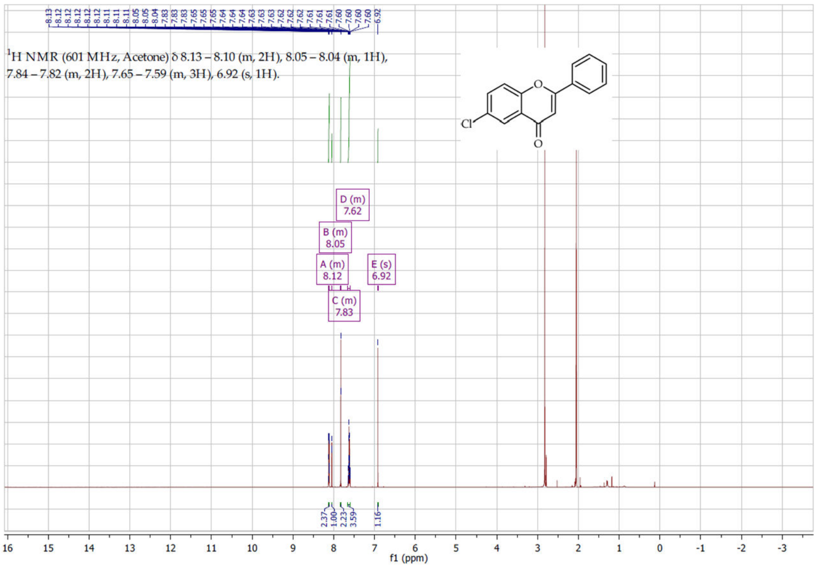

Figure S51. ^{1}H NMR spectrum (*δ,* acetone-d6, 600 MHz) of 6-chloroflavone (**D4**)

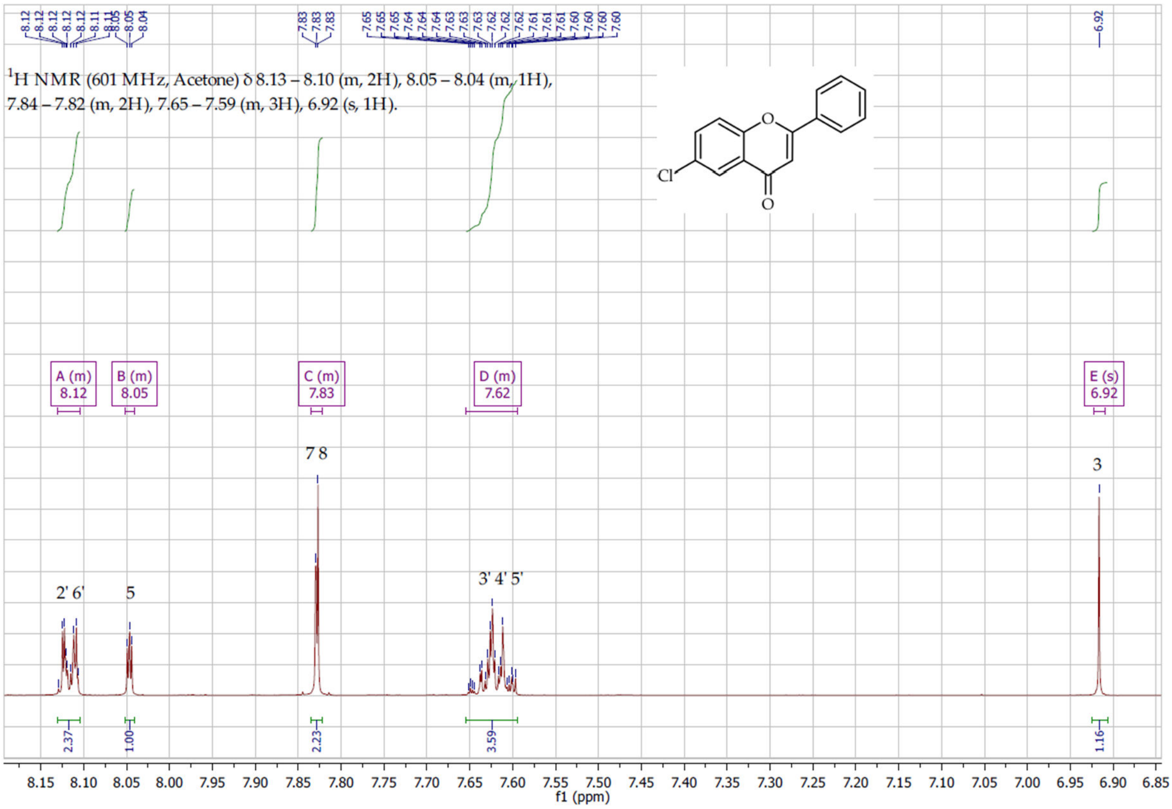

Figure S52. ^{1}H NMR spectrum expansion (*δ,* acetone-d6, 600 MHz) of 6-chloroflavone (**D4**)

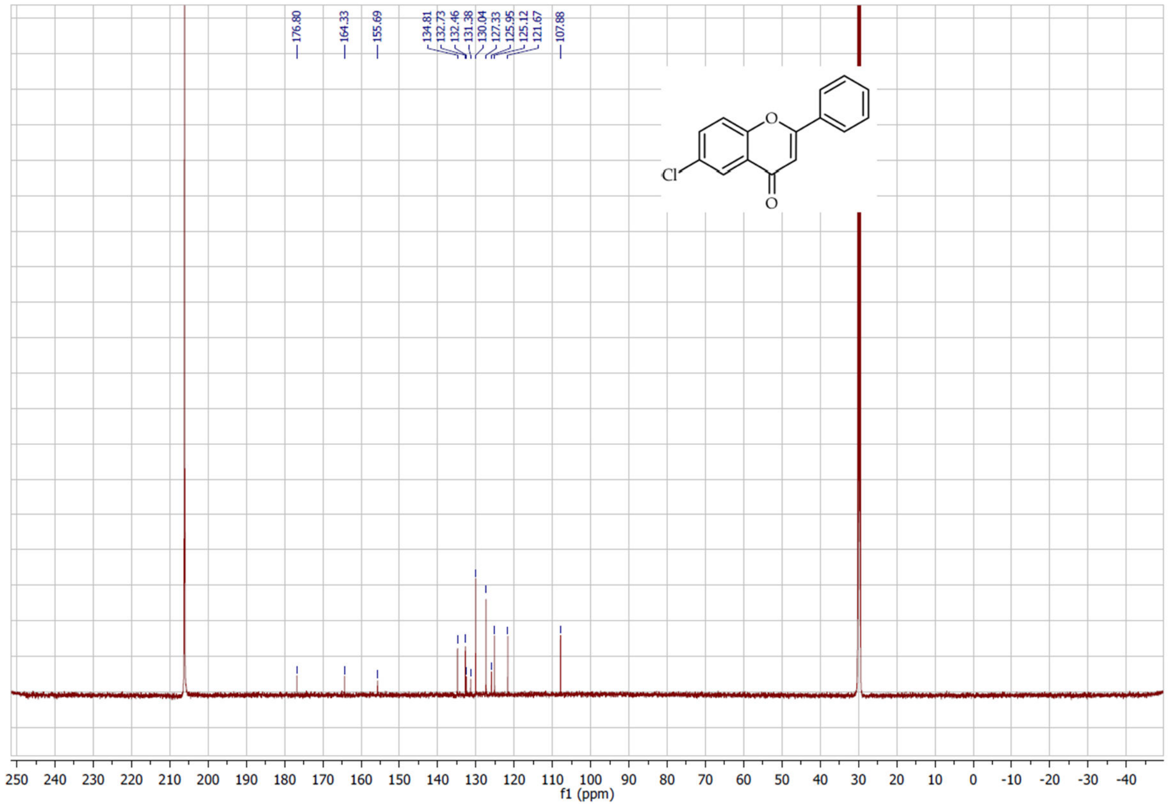

Figure S53. ^{13}C NMR spectrum (*δ,* acetone-d6, 151 MHz) of 6-chloroflavone (**D4**)

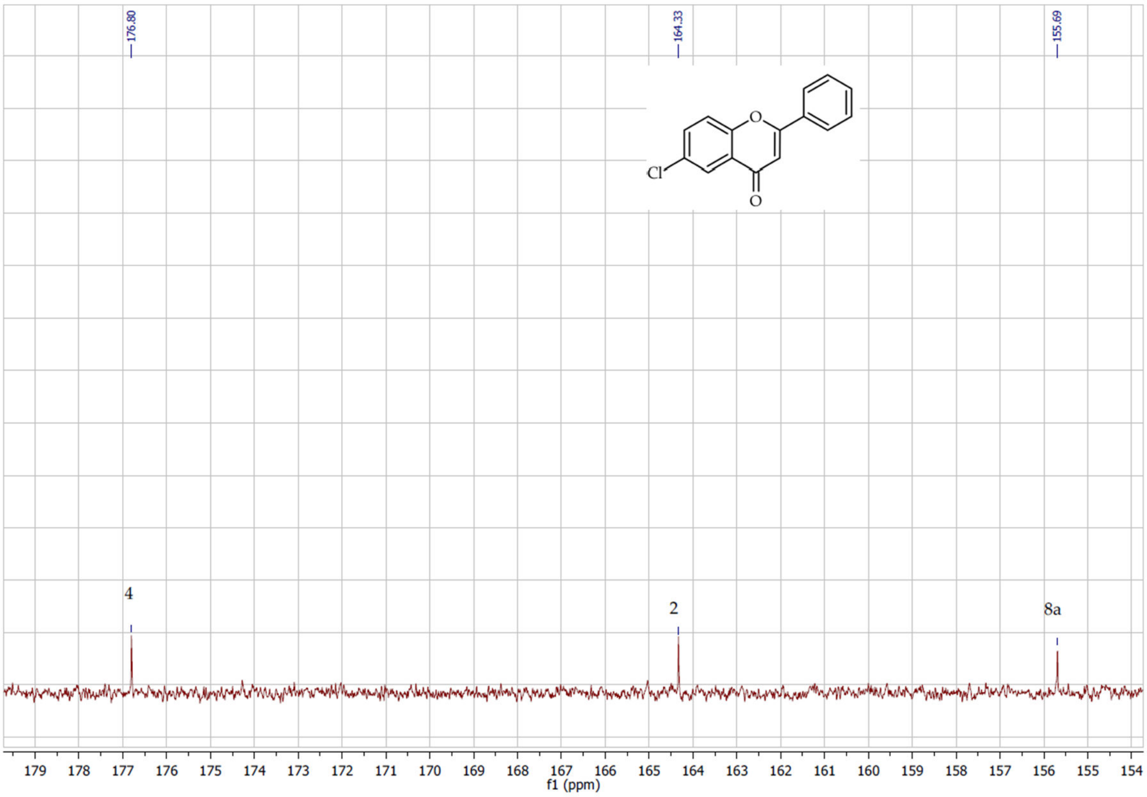

Figure S54. ^{13}C NMR spectrum expansion (*δ,* acetone-d6, 151 MHz) of 6-chloroflavone (**D4**)

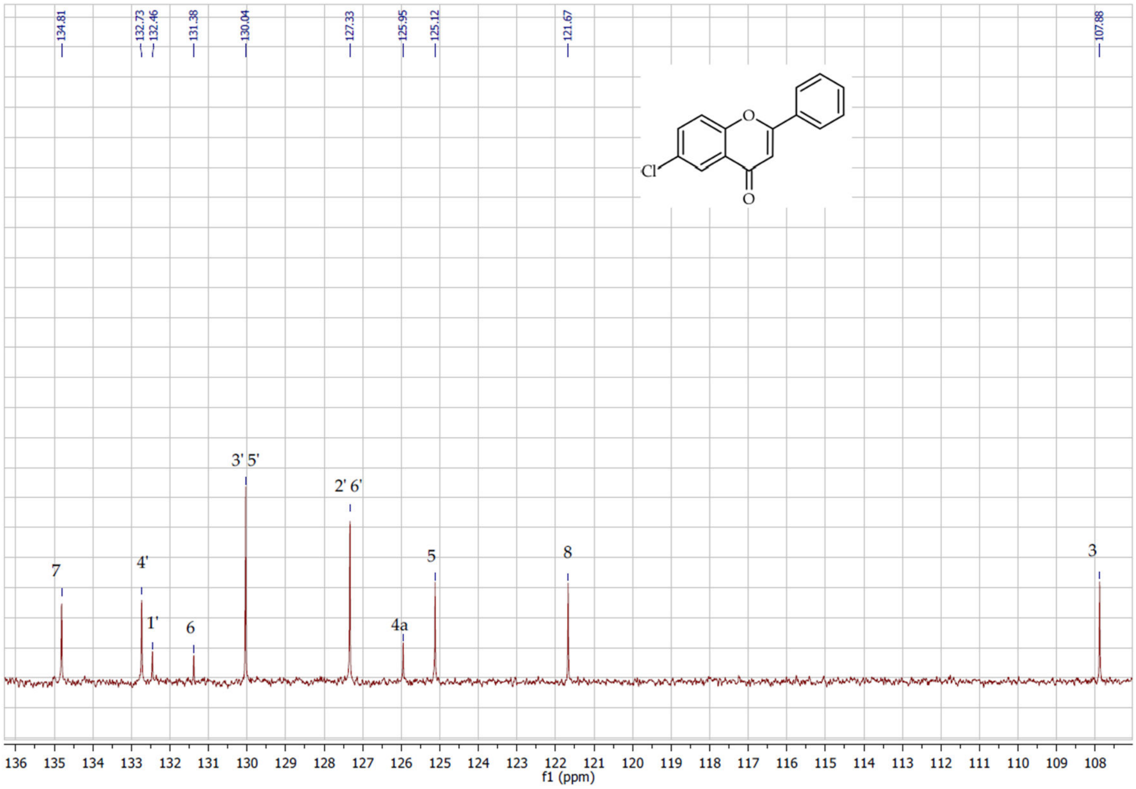

Figure S55. ^{13}C NMR spectrum expansion (*δ,* acetone-d6, 151 MHz) of 6-chloroflavone (**D4**)

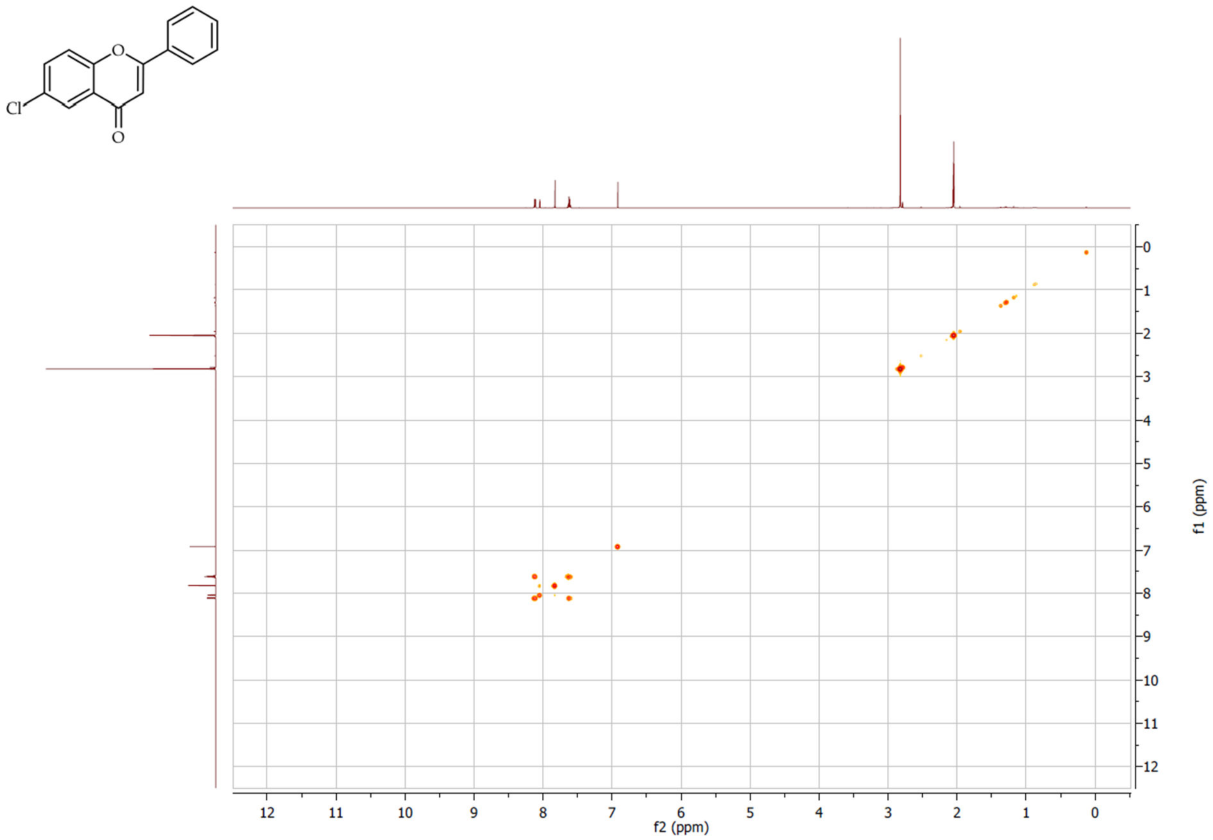

Figure S56. COSY contour map – ^{1}H x ^{1}H of 6-chloroflavone (**D4**)

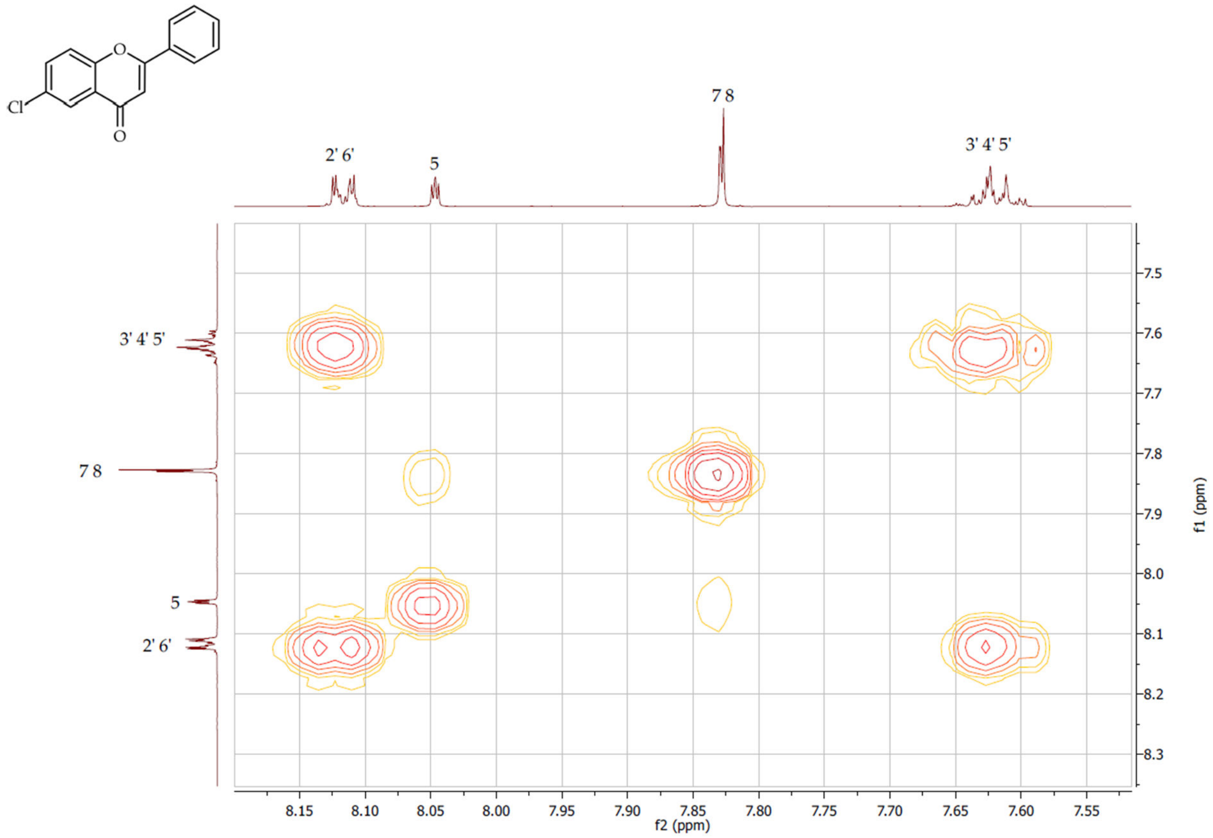

Figure S57. COSY contour map – ^{1}H x ^{1}H expansion of 6-chloroflavone (**D4**)

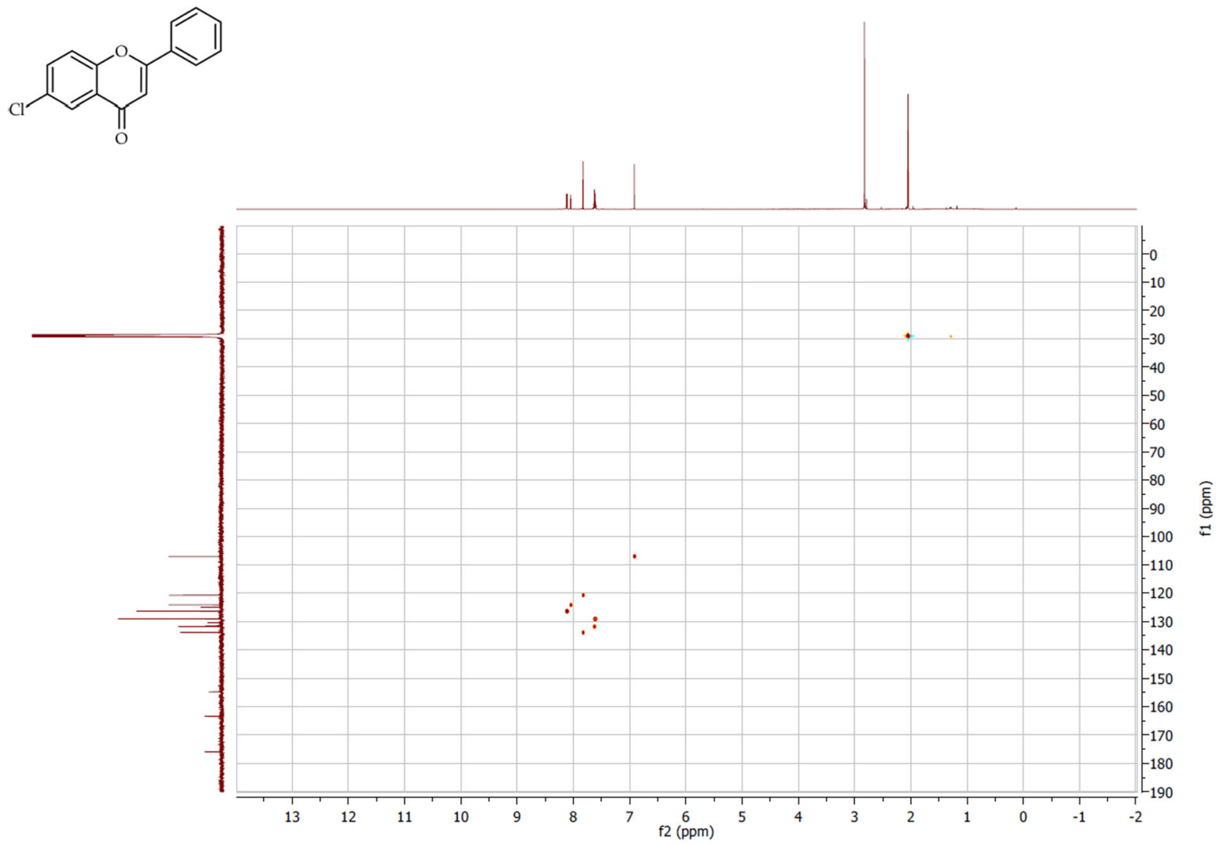

Figure S58. HMQC contour map – ^1H x ^{13}C of 6-chloroflavone (**D4**)

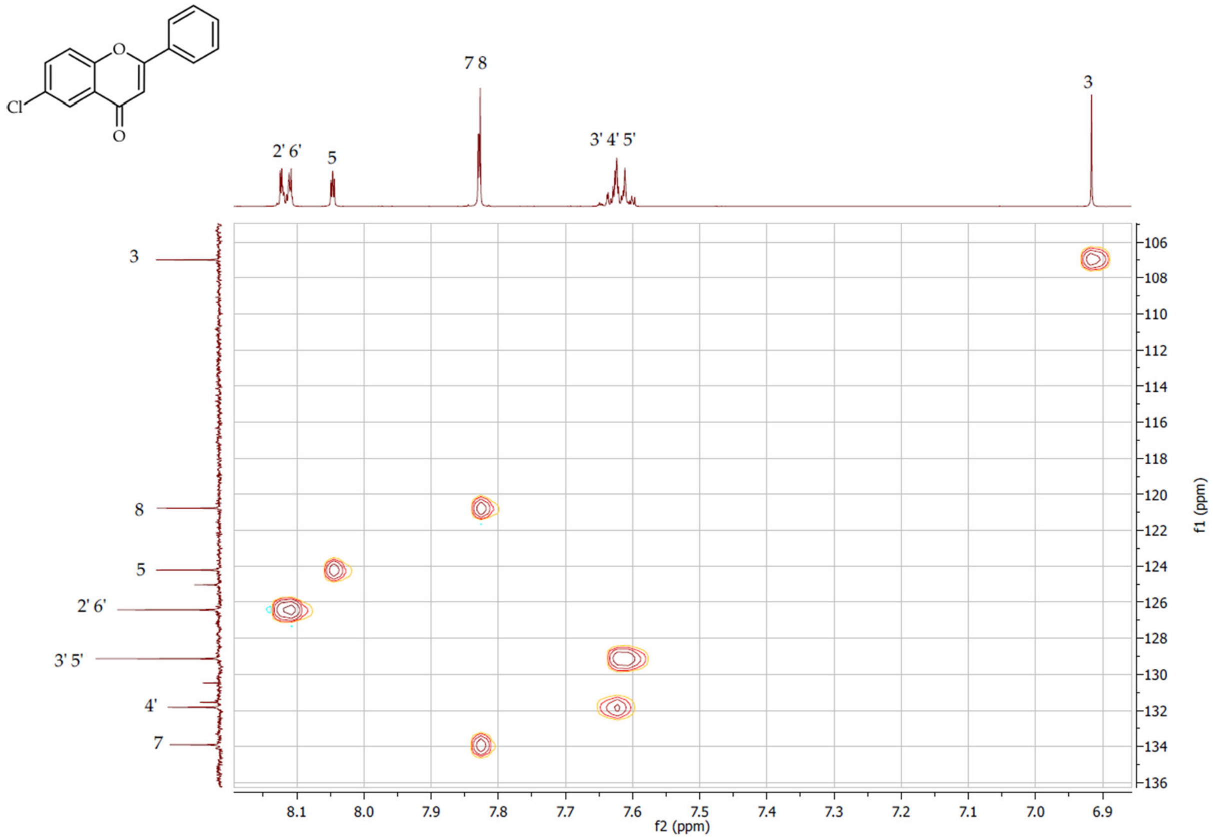

Figure S59. HMQC contour map – ^1H x ^{13}C expansion of 6-chloroflavone (**D4**)

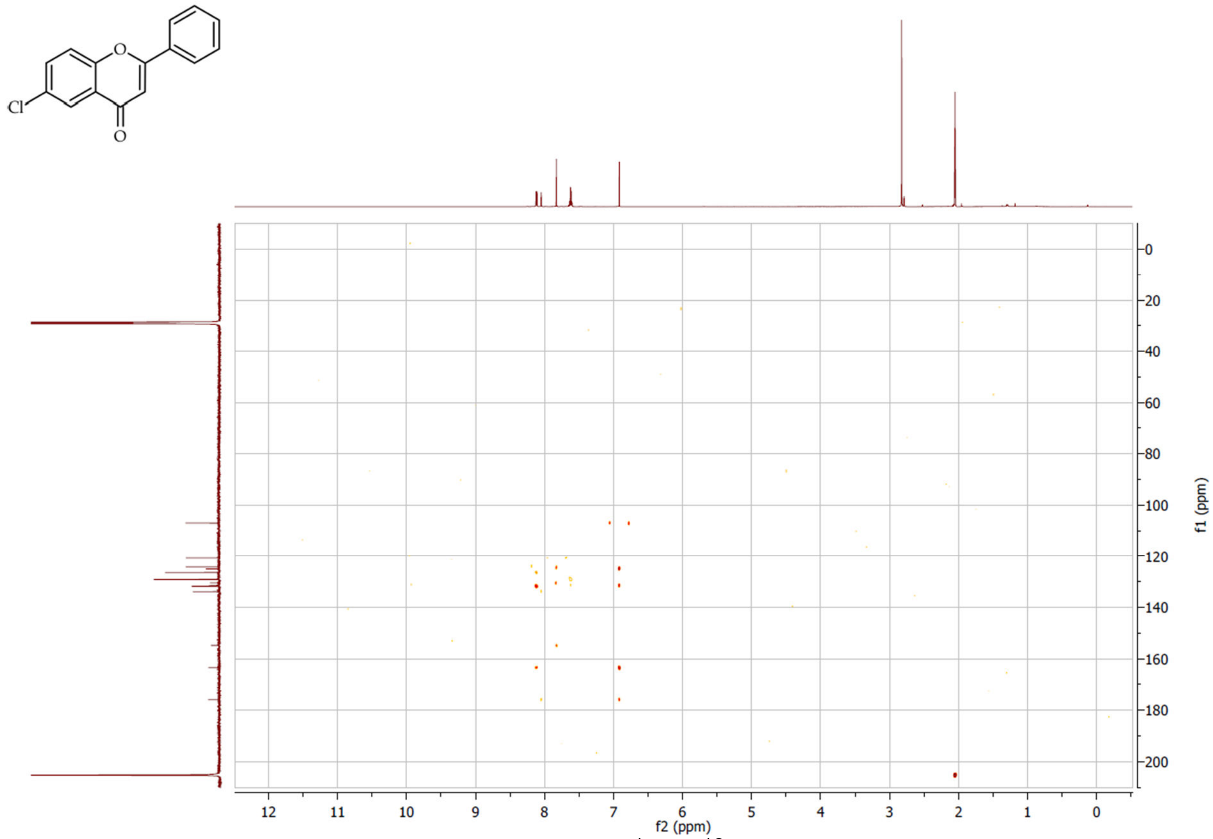

Figure S60. HMBC contour map – ^{1}H x ^{13}C of 6-chloroflavone (**D4**)

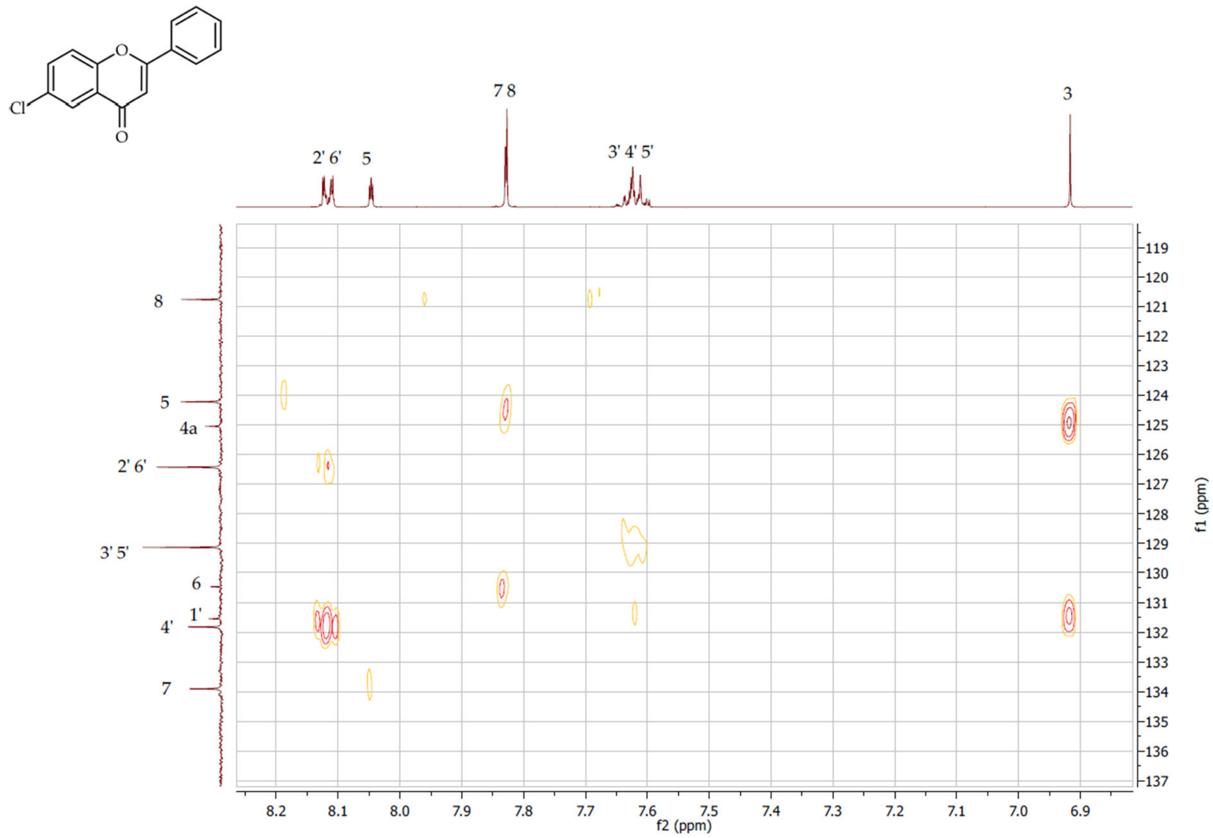

Figure S61. HMBC contour map – ^{1}H x ^{13}C expansion of 6-chloroflavone (**D4**)

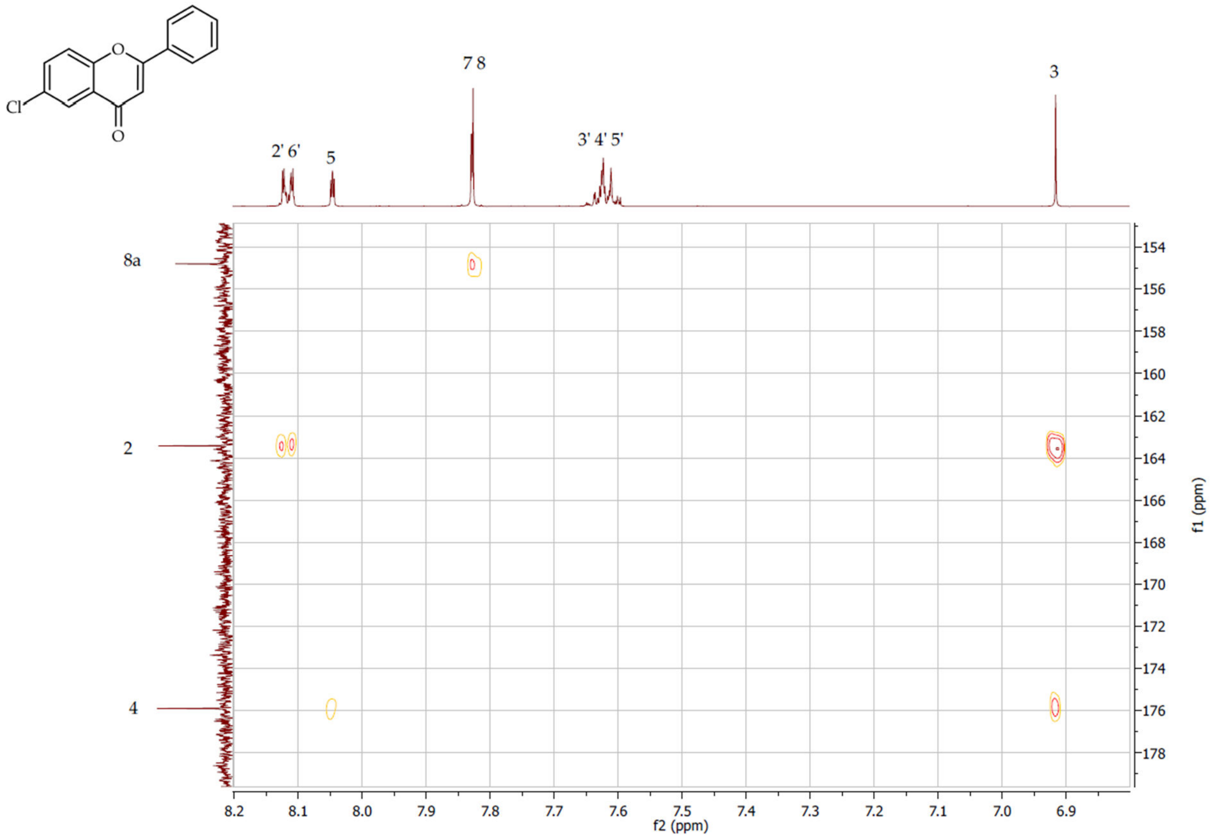

Figure S62. HMBC contour map – $^{1}H \times {}^{13}C$ expansion of 6-chloroflavone (**D4**)

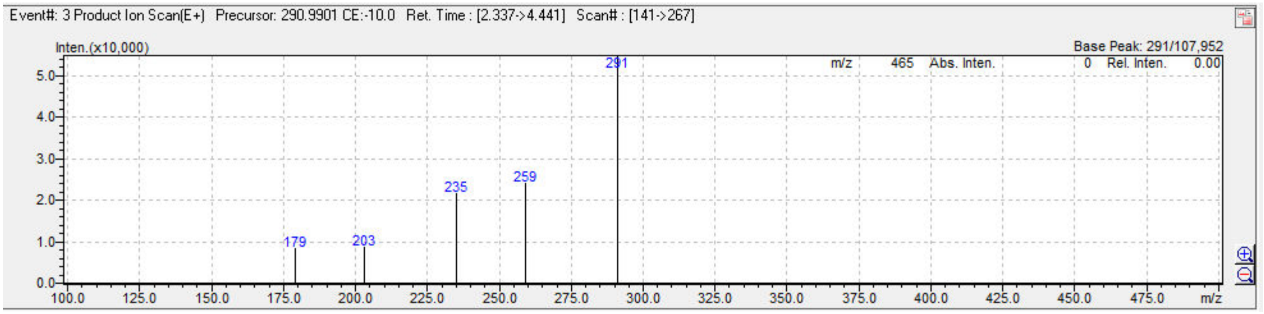

Figure S63. MS analysis of 6,8-dichloroflavone (**D5**)

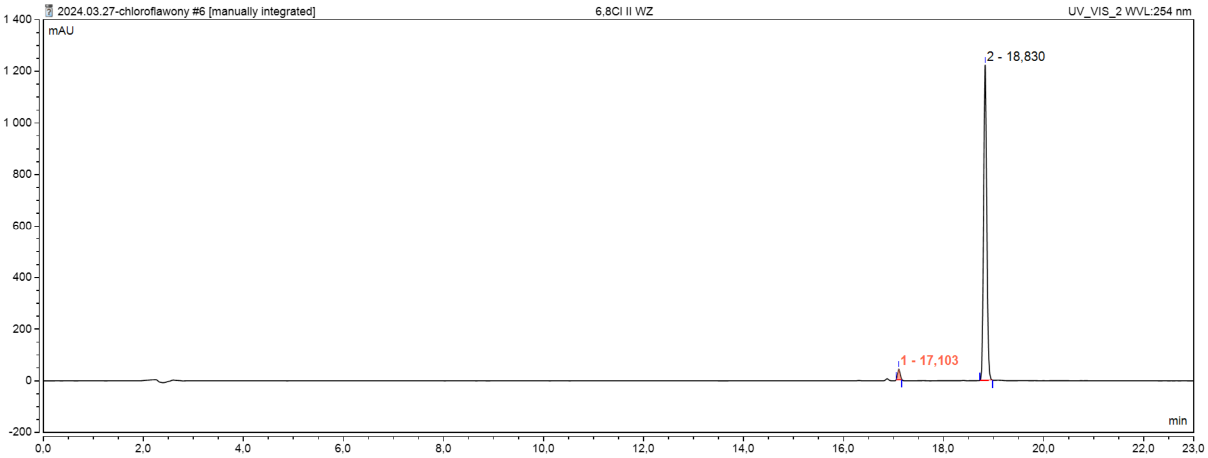

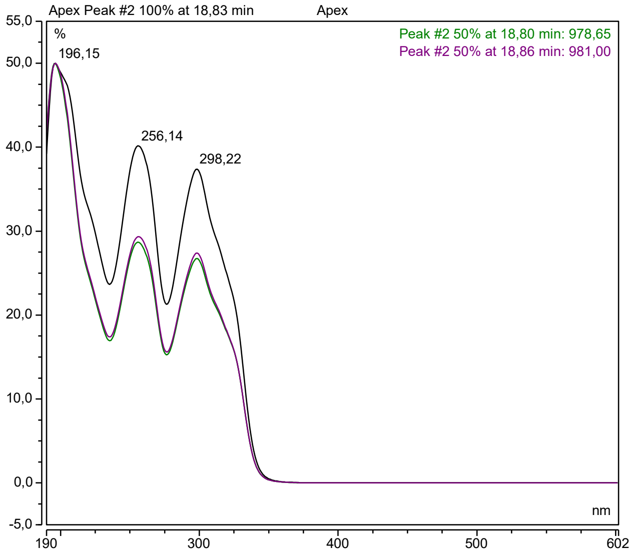

Peak	Peak Name	Ret.Time	Amount	Rel.Area	Area	Height	Type	Width (50%)	Asym.	Resol.	Plates
No.		min	n.a.	%	mAU*min	mAU		min	EP	EP	EP
UV_VIS_2	UV_VIS_2	UV_VIS_2	UV_VIS_2	UV_VIS_2	UV_VIS_2	UV_VIS_2	UV_VIS_2	UV_VIS_2	UV_VIS_2	UV_VIS_2	UV_VIS_2
1		17,103	n.a.	2,57	2,3493	42,18	BMB*	0,054	1,07	16,77	552962
2		18,830	n.a.	97,43	88,8888	1221,90	BMB*	0,067	1,08	n.a.	433042
Maximum			0,0000	97,43	88,8888	1221,90		0,067	1,08	16,77	552962
Minimum			0,0000	2,57	2,3493	42,18		0,054	1,07	16,77	433042
Sum			0,0000	100,00	91,2381	1264,08					

Figure S64. HPLC analysis of 6,8-dichloroflavone (**D5**)

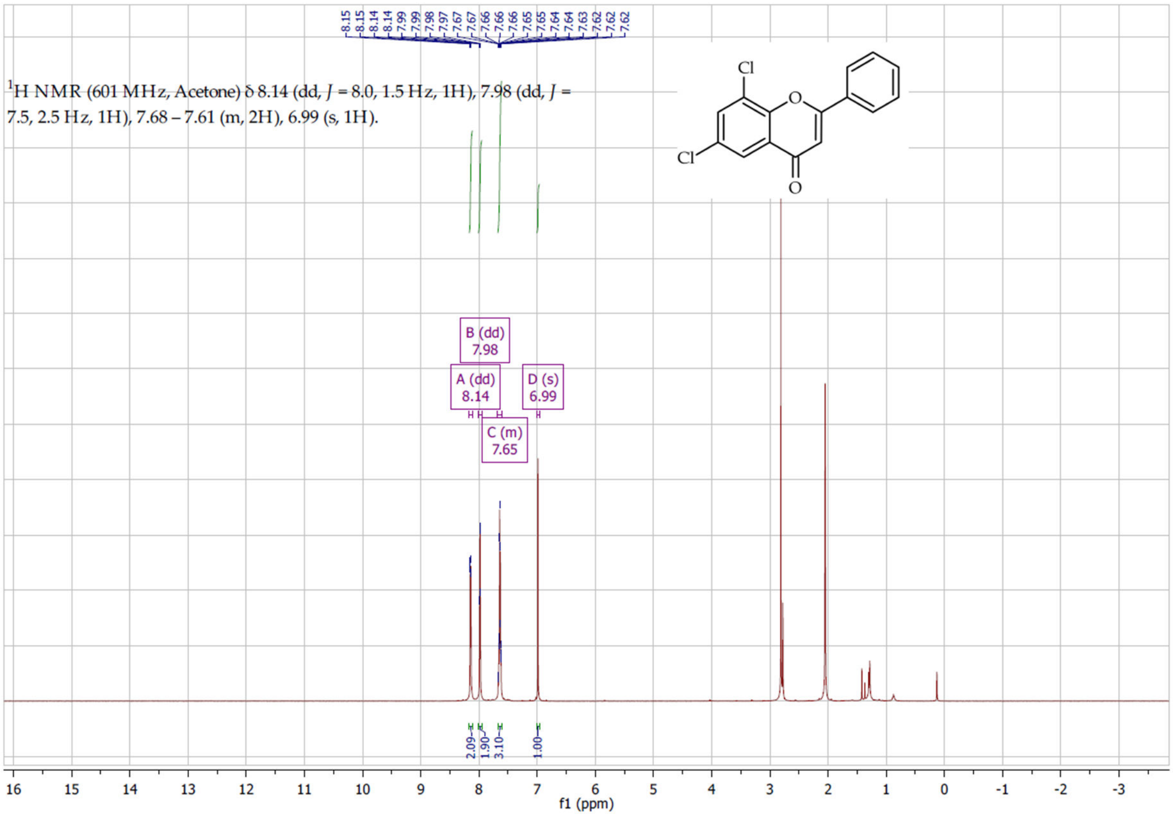

Figure S65. ^{1}H NMR spectrum (*δ,* acetone-d6, 600 MHz) of 6,8-dichloroflavone (**D5**)

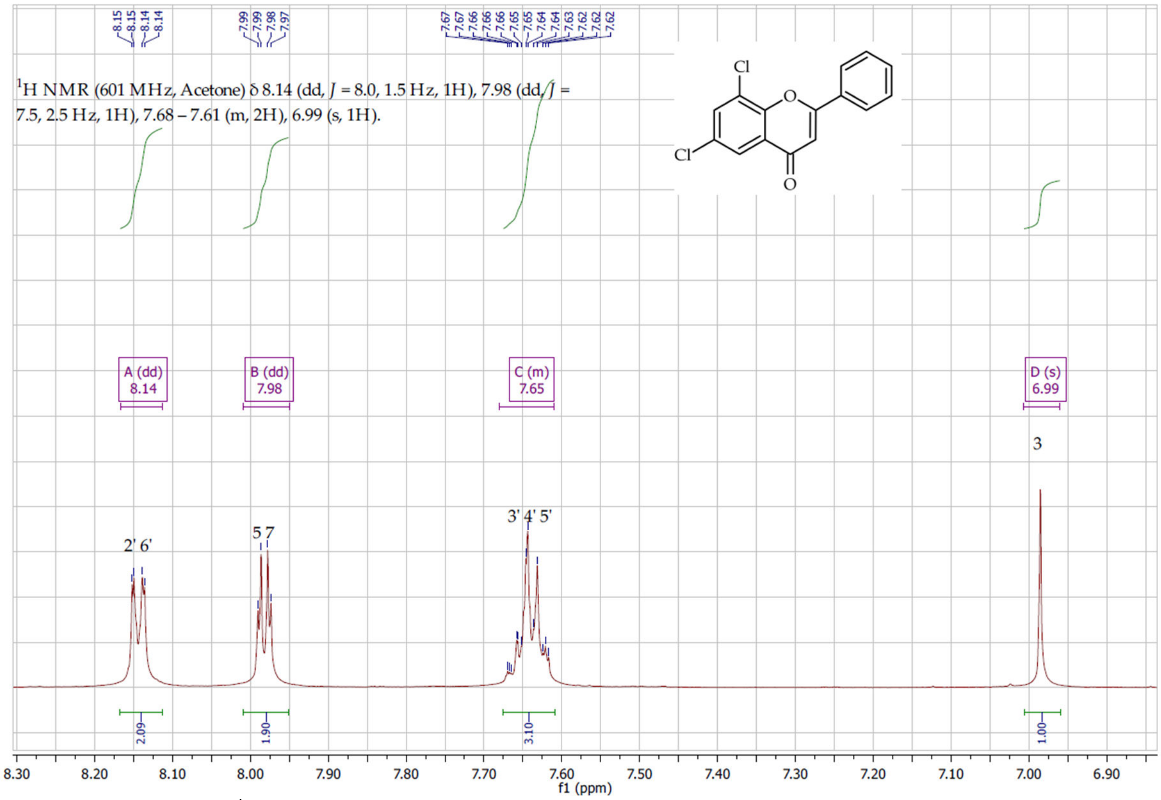

Figure S66. ^{1}H NMR spectrum expansion (*δ,* acetone-d6, 600 MHz) of 6,8-dichloroflavone (**D5**)

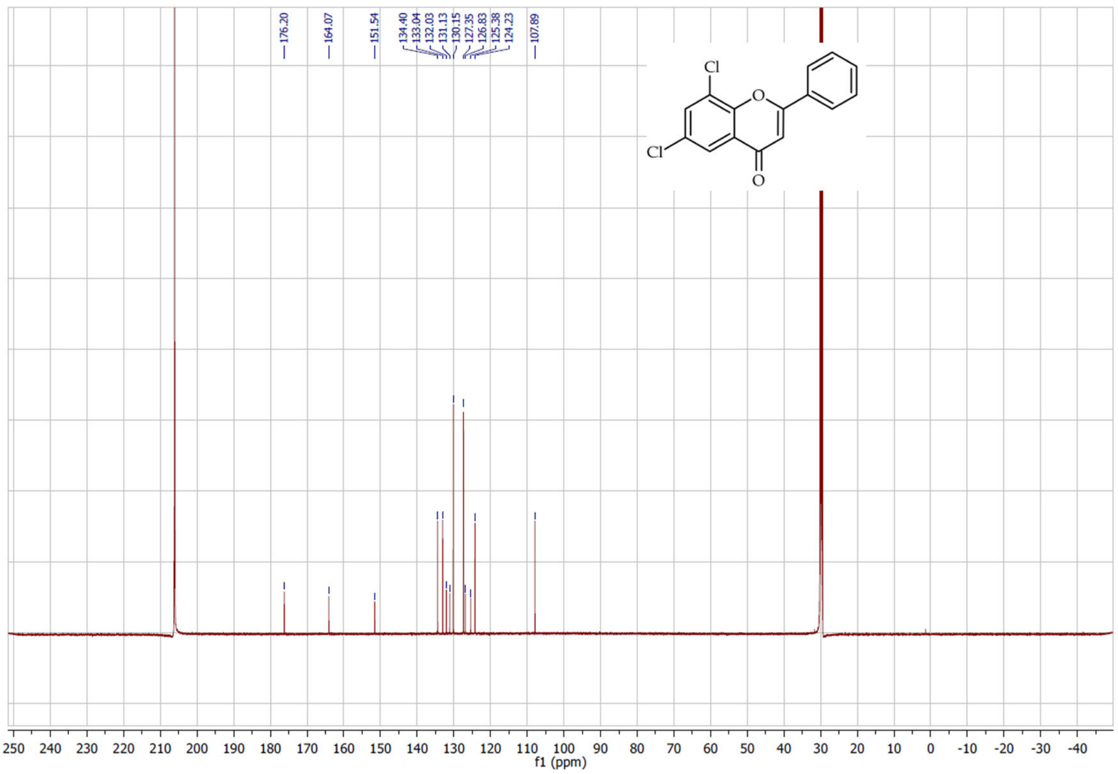

Figure S67. ^{13}C NMR spectrum (*δ,* acetone-d6, 151 MHz) of 6,8-dichloroflavone (**D5**)

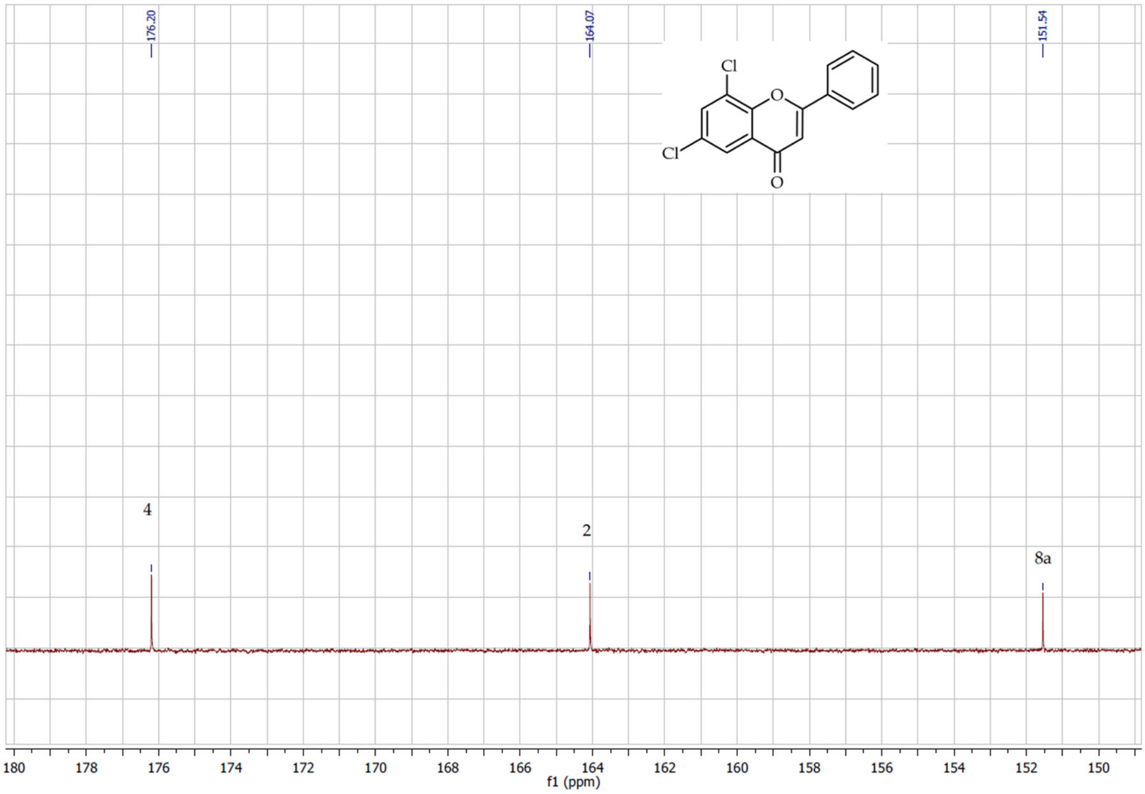

Figure S68. ^{13}C NMR spectrum expansion (*δ,* acetone-d6, 151 MHz) of 6,8-dichloroflavone (**D5**)

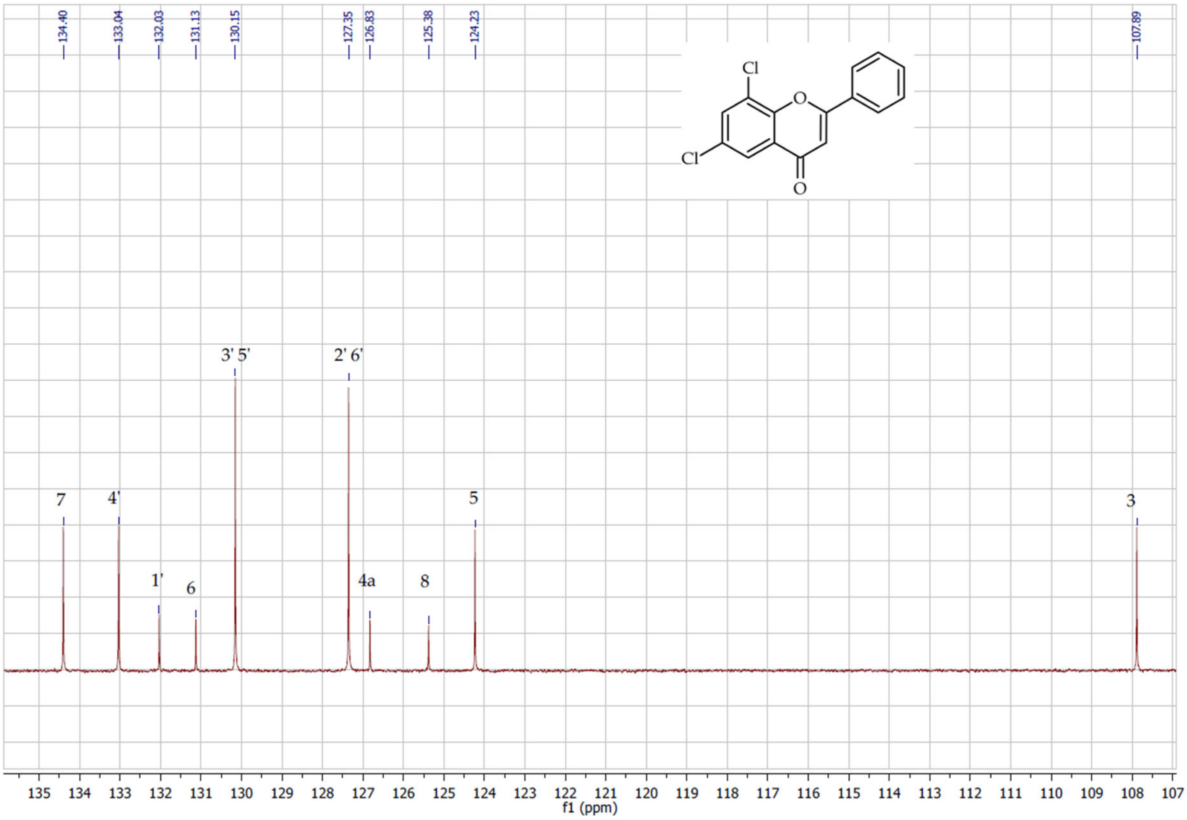

Figure S69. ^{13}C NMR spectrum expansion (*δ,* acetone-d6, 151 MHz) of 6-dichloroflavone (**D5**)

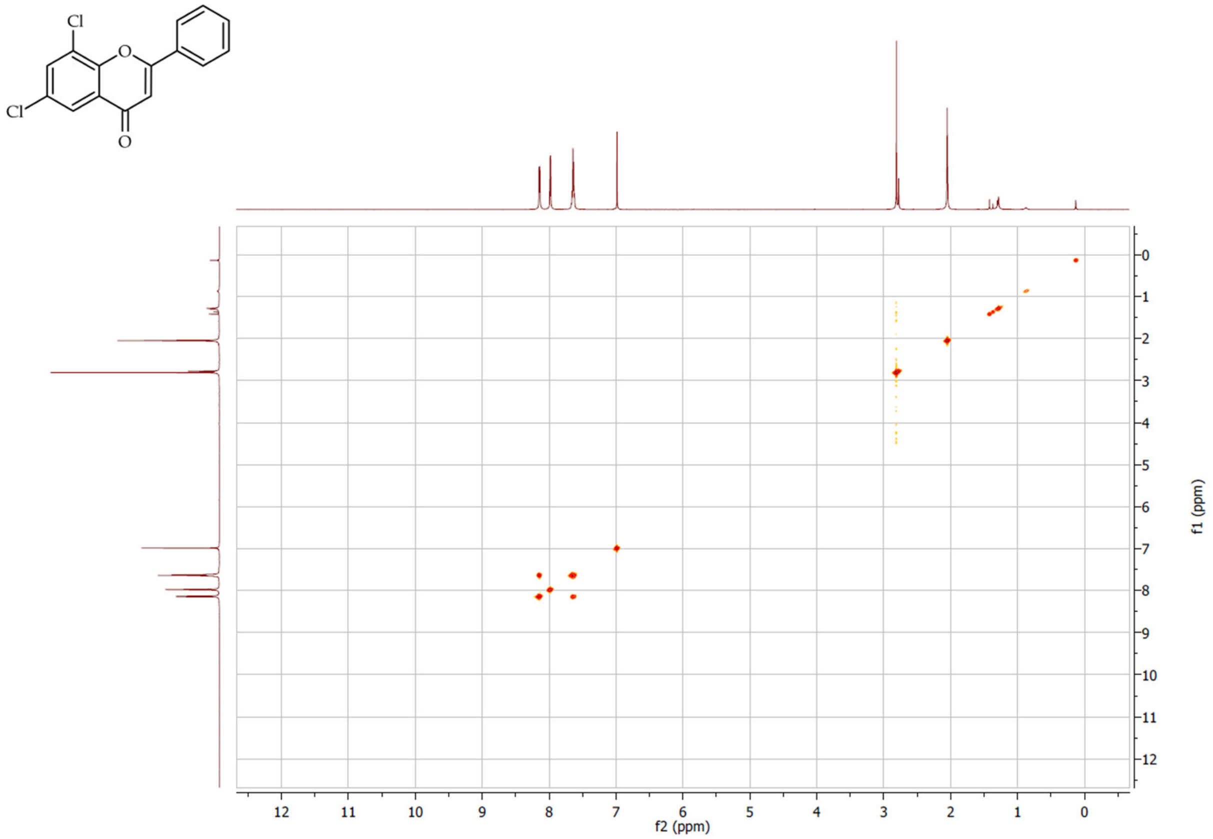

Figure S70. COSY contour map – ^{1}H x ^{1}H of 6,8-dichloroflavone (**D5**)

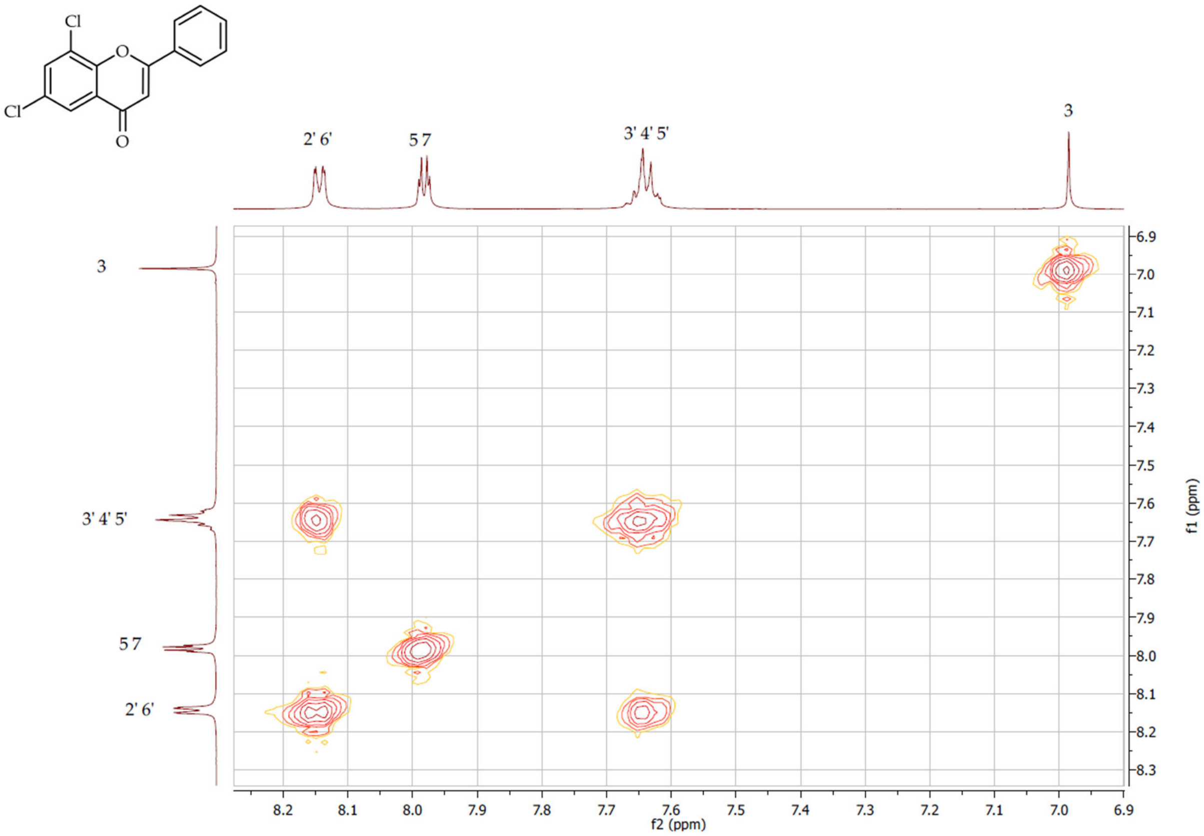

Figure S71. COSY contour map – ^{1}H x ^{1}H expansion of 6,8-dichloroflavone (**D5**)

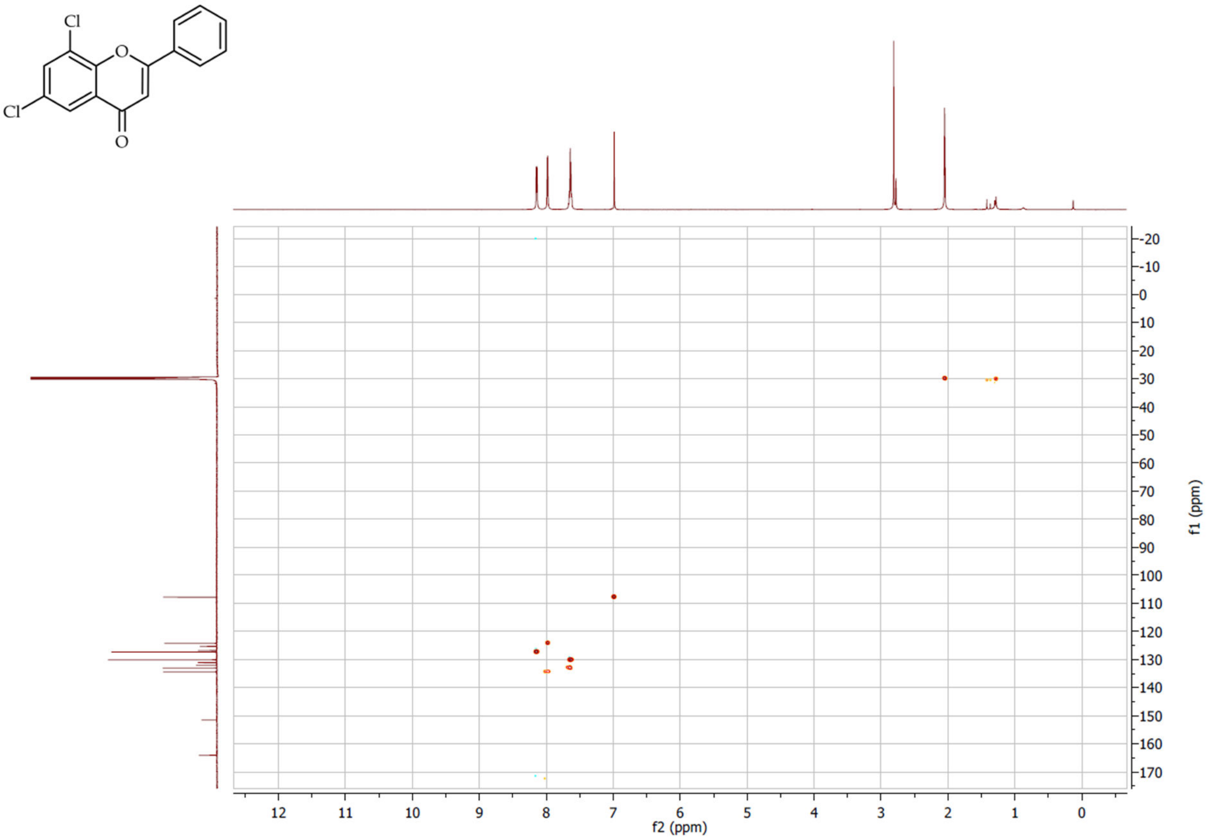

Figure S72. HMQC contour map – ^{1}H x ^{13}C of 6,8-dichloroflavone (**D5**)

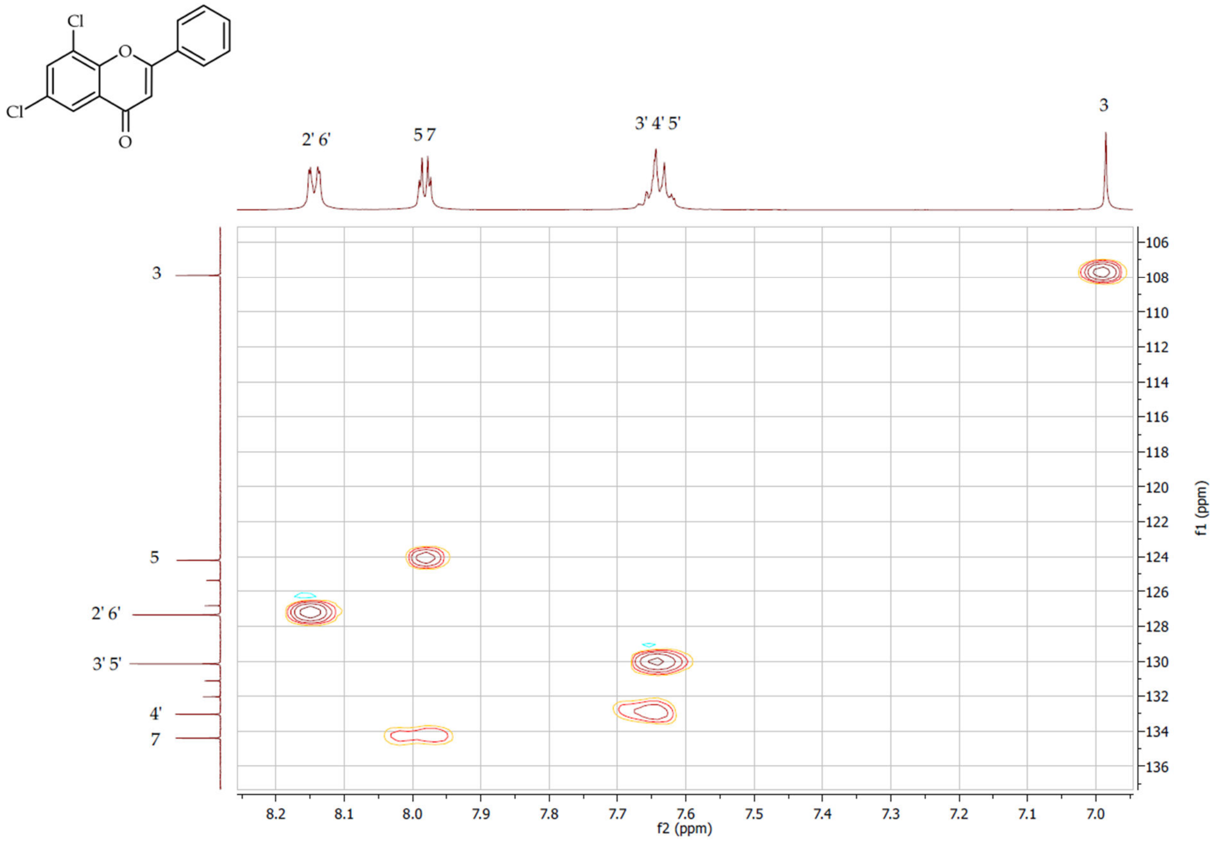

Figure S73. HMQC contour map – ^{1}H x ^{13}C expansion of 6,8-dichloroflavone (**D5**)

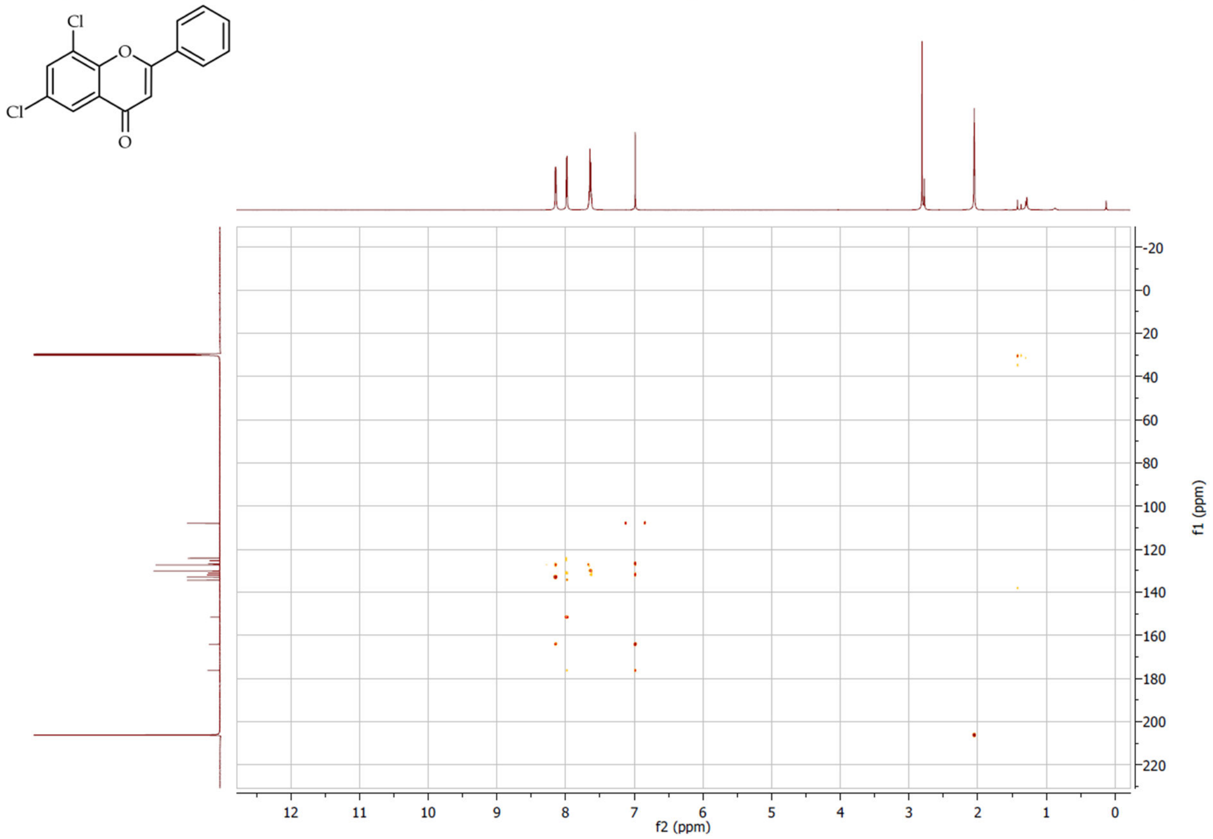

Figure S74. HMBC contour map – ^{1}H x ^{13}C of 6,8-dichloroflavone (**D5**)

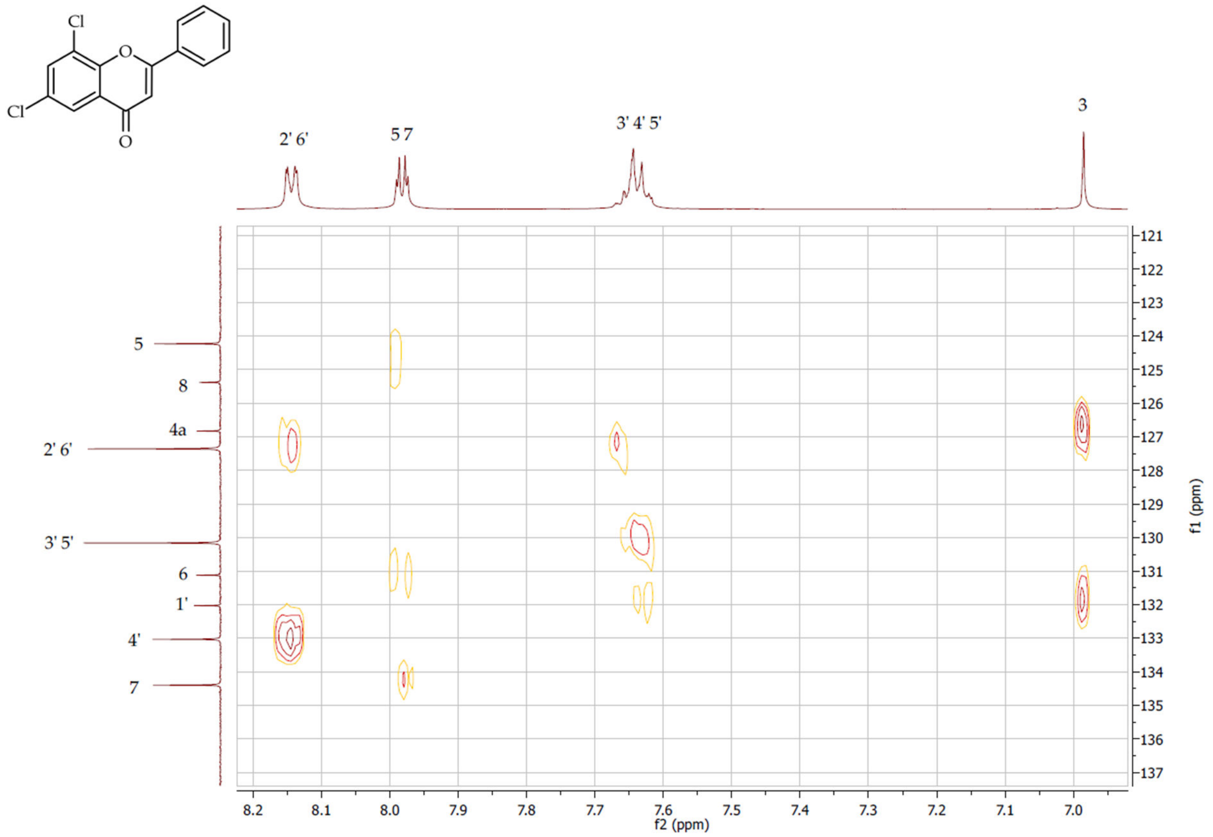

Figure S75. HMBC contour map – ^{1}H x ^{13}C expansion of 6,8-dichloroflavone (**D5**)

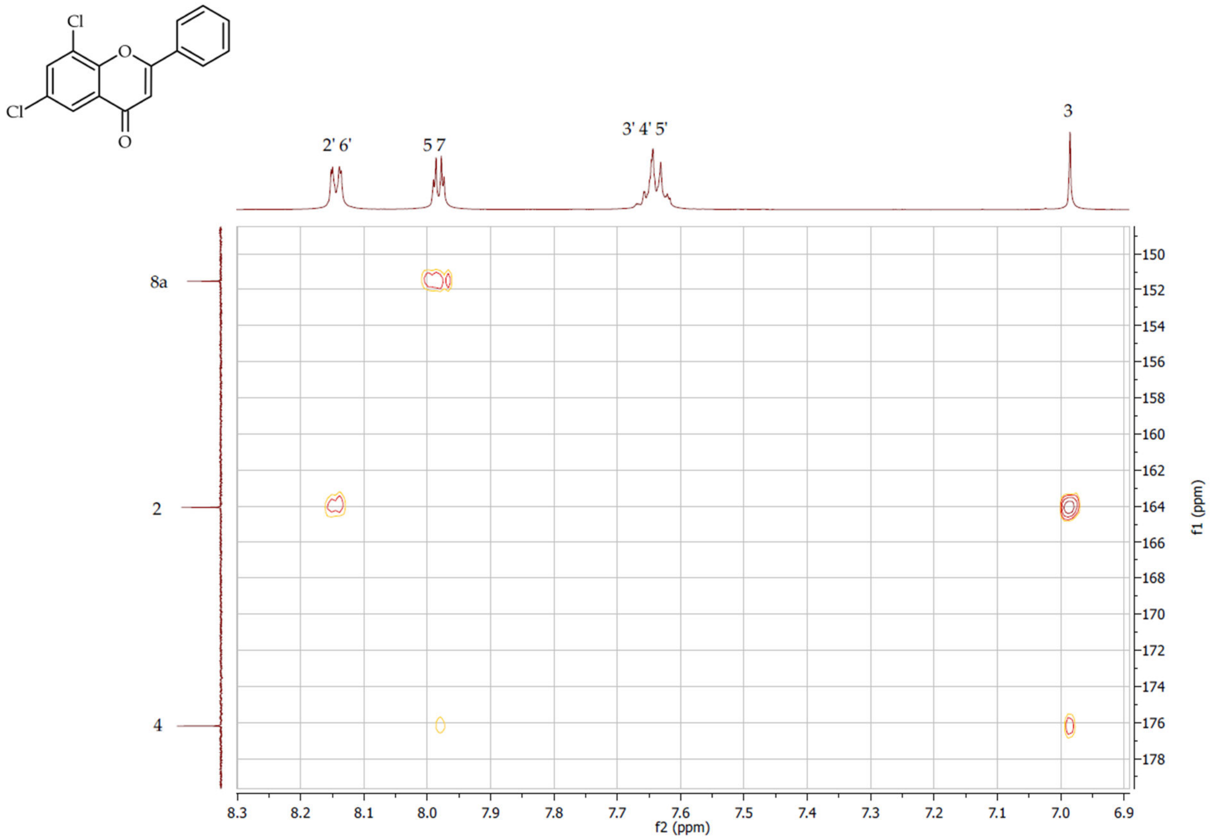

Figure S76. HMBC contour map – ^{1}H x ^{13}C expansion of 6,8-dichloroflavone (**D5**)
